# Photochemical Wolff Rearrangement Initiated Generation and Subsequent α-Chlorination of C1 Ammonium Enolates

**DOI:** 10.1021/acs.orglett.3c00986

**Published:** 2023-04-25

**Authors:** David Weinzierl, Magdalena Piringer, Paul Zebrowski, Lotte Stockhammer, Mario Waser

**Affiliations:** † Institute of Organic Chemistry, Johannes Kepler University Linz, Altenbergerstr. 69, 4040 Linz, Austria

## Abstract

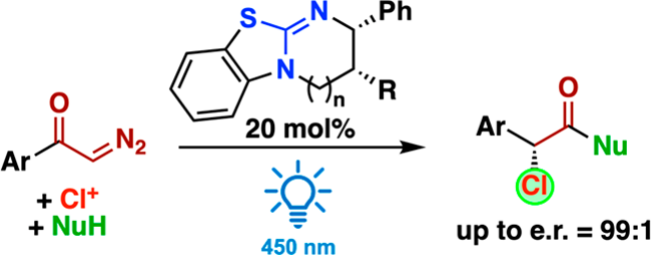

The enantioselective synthesis of α-chlorinated carboxylic acid esters with er up to 99:1 and yields up to 82% was achieved via a one-pot multistep protocol starting from α-diazoketones. This process proceeds via a photochemical Wolff rearrangement, trapping of the generated ketene with a chiral Lewis base catalyst, subsequent enantioselective α-chlorination, and a final nucleophilic displacement of the bound catalyst. The obtained products were successfully utilized for stereospecific nucleophilic displacement reactions with *N*- and *S*-nucleophiles.

Catalytic enantioselective α-halogenation reactions of prochiral enolate precursors are of high importance to access valuable chiral building blocks and target molecules.^1,2^ Asymmetric organocatalysis^3,4^ has contributed significantly to the advancement of the field, and a variety of powerful methods to facilitate asymmetric α-halogenations of important compound classes, such as amino acid derivatives,^5^ β-ketoesters,^6^ oxindoles,^7^ or other (acyclic) carbonyl derivatives,^2^ have been reported. In general, the asymmetric α-halogenation of cyclic pronucleophiles using noncovalent organocatalytic activation modes, like ion-pairing catalysis^8^ and the α-halogenation of acyclic aldehydes and ketones using enamine catalysis,^9^ have been very impressively developed over the last two decades.^1,2,5−7^ The catalytic α-halogenation of simple acyclic carboxylic acid derivatives has been a more challenging task, however. The use of chiral Lewis bases as nucleophilic organocatalysts has emerged as a powerful concept to activate and control transformations of simple acyclic pronucleophiles via in situ formation of C1 ammonium enolates.^10−13^ The most valuable catalysts of choice, therefore, are usually chiral tertiary amines (i.e., Cinchona alkaloids), *N*-heterocyclic carbenes (NHCs), pyridine derivatives, and isothioureas (ITUs).^10−13^ These readily available catalysts react with ketenes or activated carboxylic acid derivatives (aryl esters, chlorides, or anhydrides) to form chiral C1 ammonium enolates, which can then react with different electrophiles followed by a final release of the catalyst upon attack of a nucleophile (Scheme 1A).^10−13^

**Scheme 1:**
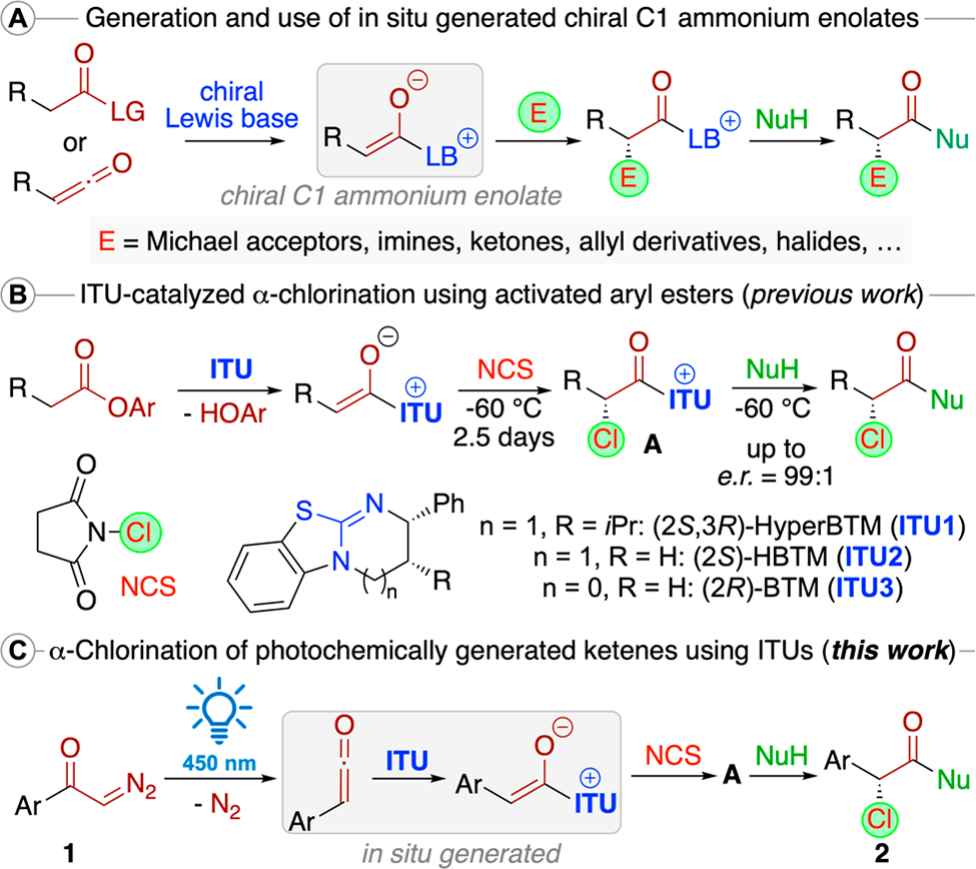
Chiral C1 Ammonium Enolates, Our Previous α-Chlorination Protocol, and the Herein Investigated α-Chlorination Using Photochemically Generated Ketenes

This powerful catalysis concept has mainly been very successfully exploited for asymmetric C–C bond forming reactions, and easily accessible chiral ITUs emerged as an especially promising class of chiral Lewis base catalysts within this field.^11−15^ In contrast to the broad variety of reported C–C bond forming approaches, the asymmetric α-heterofunctionalizations of C1 ammonium enolates generated in situ have received less attention,^16−19^ although early reports by the groups of Lectka,^16,17^ Fu,^18^ and Smith^19^ impressively outlined the potential of chiral Lewis base catalysis to access chiral α-halogenated carboxylic acid derivatives.

Very recently, Zheng and co-workers reported an enantioselective ITU-catalyzed α-fluorination strategy,^20^ and our group introduced a conceptually similar ITU-catalyzed α-chlorination protocol (Scheme 1B).^21^ While Zheng et al. started from free carboxylic acids, which were activated in situ with TsCl,^20^ we utilized aryl esters as pronucleophiles.^21^ Although reliant on different starting materials, both approaches proceed via in situ formation of a chiral C1 ammonium enolate. In general, ITUs are mainly used upon employing (in situ) generated anhydrides and activated ester starting materials.^11−13^ Ketenes, however, have so far primarily been used as C1 ammonium enolate precursors in combination with Cinchona alkaloids, NHCs, or pyridine derivatives.^10,22^ Ketenes can be accessed by different strategies, with the photoactivated Wolff rearrangement of α-diazoketones being one of the synthetically most appealing.^23^ Impressively, when this photochemical process is carried out in the presence of Lewis bases, like NHCs or Cinchona alkaloids, the direct formation and utilization of (chiral) C1 ammonium enolates thereof is possible.^16c,24^ In one single example, Lectka et al. also proved that such photogenerated ketenes can be utilized for enantioselective α-chlorinations in the presence of Cinchona alkaloids.^16c^ Inspired by the elegance of this strategy and two recent reports describing the trapping and further transformation of photogenerated ketenes with ITUs,^25^ we wondered if it may be possible to utilize α-diazoketones **1** directly under one-pot photochemical conditions in the presence of ITUs, an electrophilic halide source [like *N*-chlorosuccinimide (NCS)], and a nucleophile to access enantioenriched α-chlorinated carboxylic acid derivatives **2** directly (Scheme 1C). This would result in a complementary approach, compared with our recent aryl ester-based strategy (Scheme 1B), by relying on an alternative class of easily available starting materials. In addition, our recent protocol required long reaction times (2.5 days) and a careful temperature control for both the chlorination and MeOH quench (−60 °C) to avoid epimerization of the catalyst-bound α-Cl-intermediate **A**, and we are confident that the herein investigated one-pot strategy will allow us to overcome these operational limitations by directly trapping **A** with the present nucleophile.

We started our investigations by testing the reaction of the α-diazoketone **1a** under blue LED irradiation (450 nm) in the presence of the established chiral catalysts **ITU1**–**3** (Scheme 1B),^14,15^ NCS, and MeOH (Table 1 gives an overview about the most significant screening results). We were glad to see that literally the first screening attempts with HyperBTM (**ITU1**, entries 1 and 2) supported our working hypothesis by yielding the α-chlorinated methylester **2a** with promising initial levels of enantioselectivities. Interestingly, while toluene and CH_2_Cl_2_ allowed for good conversions of **1a**, THF was found to be not suited (entry 3). Further testing of different chiral ITUs allowed us to identify benzotetramisole (BTM) (**ITU3**) as the most selective catalyst when using toluene as the solvent (entry 5; other aromatic solvents were tested, too, but no improvement was possible). Fine tuning of the reaction conditions with **ITU3** showed that temperature control is an important factor (entries 5–7). When the reactions were carried out at rt without any cooling, the reaction temperature usually rose to around 40 °C under the photochemical conditions. These conditions allowed for good enantioselectivities (er = 95:5), but some minor amounts of different not-identified side products were formed. The reaction, however, performed with better selectivity (er = 97:3), cleaner, and higher yielding (>70% isolated yield also on 1 mmol scale) when carried out at 0 °C (entry 6), while lower temperatures were not beneficial anymore (entry 7). Unfortunately, catalyst loadings below 20 mol % were found to be less suited (entry 8), while higher loadings did not improve the outcome any further (entry 9). We, of course, also tested different concentrations, stoichiometric ratios of reagents, and reaction times but, overall, we found the conditions outlined in entry 6 to be the best-suited (also on 1 mmol scale). In addition, the long reaction times and cryogenic conditions that were necessary when using our previous aryl ester strategy (Scheme 1B)^21^ could be successfully avoided. With respect to the observed sense of induction, we rationalize that the reaction proceeds via a well-organized O–S σ-hole-stabilized C1 ammonium enolate^11,12^ where the Si-face is blocked by the Ph ring when using (*R*)-BTM (**ITU3**).

**Table 1:**
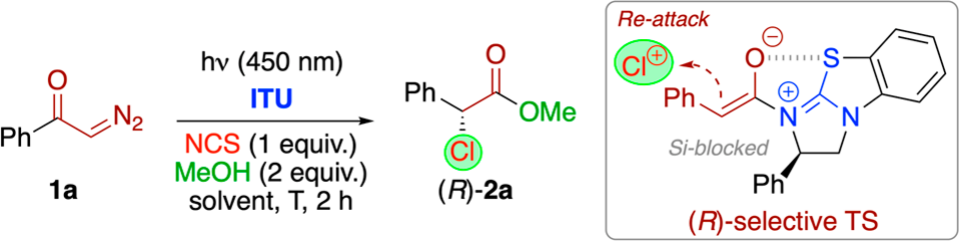
Optimization of Reaction Conditions^a^

entry	**ITU** [mol %]	solvent	*T* [°C]	conversion^b^ [%]	**2a** [%]^c^	er^d^
1	**ITU1** (20%)	CH_2_Cl_2_	25–40^e^	>95	31^IST^	80:20
2	**ITU1** (20%)	toluene	25–40^e^	>95	56^IST^	92:8
3	**ITU1** (20%)	THF	25–40^e^	n.r.		
4	**ITU2** (20%)	toluene	25–40^e^	90	40^IST^	91:9
5	**ITU3** (20%)	toluene	25–40^e^	>95	67	95:5
6	**ITU3** (20%)	toluene	0	>95	79 (72)^f^	97:3 (96:4)^f^
7	**ITU3** (20%)	toluene	–20	>95	68	90:10
8	**ITU3** (10%)	toluene	0	>95	75^IST^	65:35
9	**ITU3** (40%)	toluene	0	>95	80^IST^	96:4

a All reactions were run for 2 h using 0.1 mmol **1** and 0.1 mmol NCS in the given solvent (0.1 M with respect to **1**) unless otherwise stated.

b Conversion of **1a** judged by ^1^H NMR of the crude product.

c Given either as NMR yields (using an internal standard, IST) or as isolated yields.

d Determined by HPLC using a chiral stationary phase. Absolute configuration of the major (*R*)-enantiomer was assigned by comparison of retention time orders and its (−)- rotation with previous reports.^19,26^

e Reactions were run without cooling and warmed from rt to ca. 40 °C over the 2 h reaction period.

f On the 1 mmol scale.

Having identified operationally simple conditions for the one-pot Wolff rearrangement−α-chlorination–esterification strategy utilizing the parent α-diazoketone **1a**, we next investigated the application scope of this protocol (Scheme 2). Variations of the aryl group of **1** were generally well-tolerated (Scheme 2A) and gave methyl esters **2a**–**m** with good yields (conversions of starting materials >90%) and enantioselectivities in most cases. Only the presence of an ortho-substituent, as outlined for **2h**, somewhat lowered the enantioselectivity. Other *O*-nucleophiles were also tolerated (Scheme 2B), but the use of more bulky alcohols led to somewhat lower yields, as outlined for **2o**. The somewhat lower er with those alcohols may be explained by a slower addition to the intermediate chlorinated-catalyst-bound species **A**, which we recently identified being prone to epimerization.^21^ It is of note that even the use of 2 equiv of H_2_O worked satisfyingly (giving the free carboxylic acid **2p**). Unfortunately, the presence of amines, which would give α-Cl-amides like compound **2q**, was found to be not tolerated because the intermediate ketene was directly trapped by the amine (giving *N*-benzyl phenylacetamide) before any catalyst addition–chlorination took place.

**Scheme 2:**
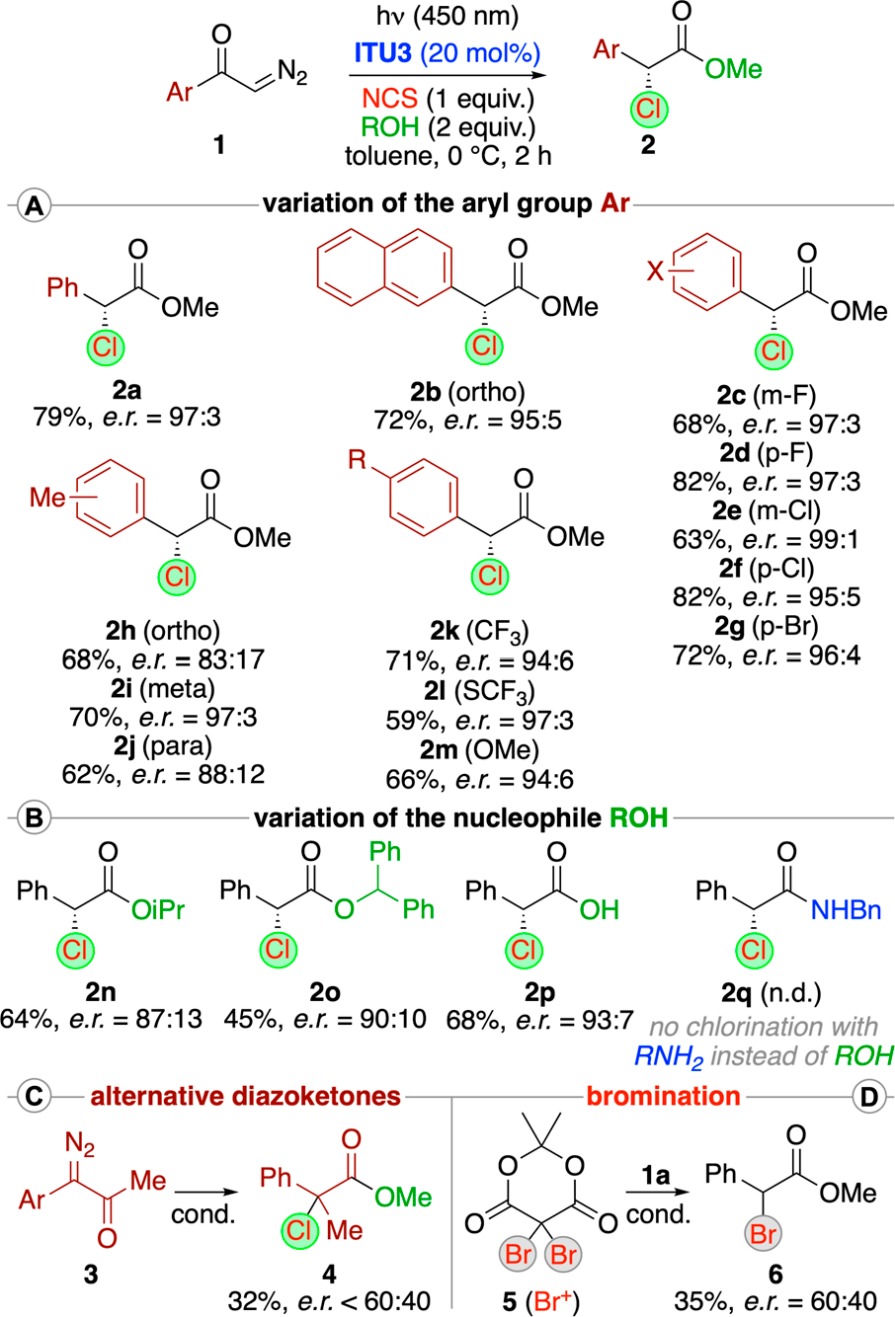
Application Scope

Additionally, we also employed the diazoketone **3**, which could be used under the standard conditions to access the α-chloro ester **4**, albeit enantioselectivity and yield were low for such conditions (Scheme 2C). Furthermore, we also tested the analogous photochemical α-bromination protocol (Scheme 2D). While NBS did not lead to any product formation, the dibromo-Meldrum’s acid **5**(27) allowed us to access product **6** (starting from **1a** under otherwise identical conditions), albeit with low enantioselectivity and yield. Although a few further attempts to improve these two reactions were not yet satisfactory, we are confident that these results represent a promising basis for future investigations to expand the scope of this methodology.

Finally, we also wanted to explore the suitability of products **2** for further manipulations. As outlined in Scheme 3, the stereospecific substitution of Cl was possible with *S*- or *N*-based nucleophiles (**2a** with er = 99:1^21^ was used to detect a potential erosion of er as accurately as possible; absolute configurations of the products were assigned upon comparison of their optical rotation with literature values^28^). While thiophenol and sodium azide allowed for the syntheses of the respective products **7** and **9** with high levels of enantiospecificity, the use of the more basic nucleophile **11** led to partial racemization during the substitution reaction (giving product **8**, a simplified analogue of the blockbuster active pharmaceutical ingredient clopidogrel^29^).

**Scheme 3:**
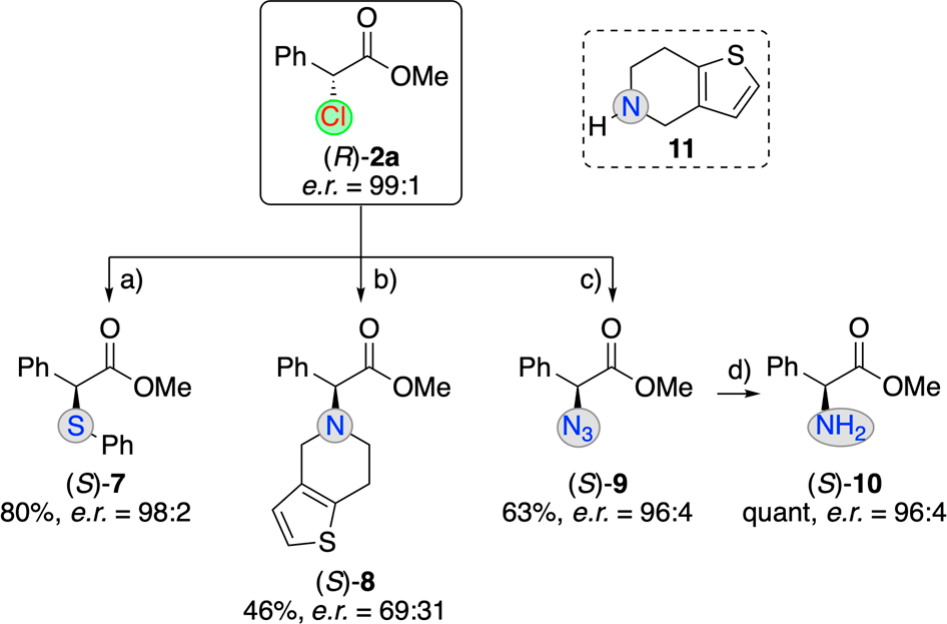
Nucleophilic Cl-Displacement PhSH (1 equiv), Hünig Base (1 equiv), acetone, rt, 3 h. Compound **11** (1 equiv), Hünig Base (1 equiv), acetone, rt, overnight. NaN_3_ (2 equiv portionwise), acetonitrile, rt, overnight. H_2_ (balloon), Pd/C, THF, rt, overnight.

In conclusion, we have introduced a one-pot protocol for the direct utilization of α-diazoketones **1** for the syntheses of enantioenriched α-chlorinated carboxylic acid esters **2**. The whole process proceeds via an initial photochemical Wolff rearrangement, followed by direct trapping of the hereby generated ketene with a chiral Lewis base catalyst, subsequent enantioselective α-chlorination, and a final nucleophilic displacement of the covalently bound catalyst. Among the readily available chiral ITUs that were tested, BTM (**ITU3**) was found to be best-suited and resulted in enantioselectivities up to er = 99:1. In addition, we also showed that the hereby obtained products can successfully be utilized for further stereospecific nucleophilic displacement reactions.

## Data Availability

The data underlying this study are available in the published article and its Supporting Information.

## Supporting Information Available

Experimental and analytic details (including NMR spectra and HPLC traces) (PDF)

## References

[ref1] Fustero S.; Remete A. M.; Kiss L.; Medio-Simon M.; Escorihuela J.; Sedgwick D. M. Asymmetric Carbon-Halogen Bond Forming Reactions (Excluding C-H Activation Processes). In Catalytic Asymmetric Synthesis, 4th ed.; Akiyama T.; Ojima I., Eds.; John Wiley & Sons, 2022; pp 491–529.

[ref2] aOestreich M. Strategies for Catalytic Asymmetric Electrophilic α-Halogenation of Carbonyl Compounds. Angew. Chem., Int. Ed. 2005, 44, 2324–2327. 10.1002/anie.200500478. 15827954

[ref3] aList B.; Maruoka K. Science of Synthesis: Asymmetric Organocatalysis, Vols. 1 and 2; Thieme, 2012.

[ref4] Garcia Mancheño O.; Waser M. Recent Developments and Trends in Asymmetric Organocatalysis. Eur. J. Org. Chem. 2023, 26, e202200950 10.1002/ejoc.202200950. PMC10091998 37065706

[ref5] Eder I.; Haider V.; Zebrowski P.; Waser M. Recent Progress in the Asymmetric Syntheses of α-Heterofunctionalized (Masked) α- and β-Amino Acid Derivatives. Eur. J. Org. Chem. 2021, 2021, 202–219. 10.1002/ejoc.202001077.

[ref6] Govender T.; Arvidsson P. I.; Maguire G. E. M.; Kruger H. G.; Naicker T. Enantioselective Organocatalyzed Transformations of β-Ketoesters. Chem. Rev. 2016, 116, 9375–9437. 10.1021/acs.chemrev.6b00156. 27463615

[ref7] Freckleton M.; Baeza A.; Benavent L.; Chinchilla R. Asymmetric Organocatalytic Electrophilic Heterofunctionalization of Oxindoles. Asian. J. Org. Chem. 2018, 7, 1006–1014. 10.1002/ajoc.201800146.

[ref8] aOtrevel J.; Waser M. Asymmetric Phase-Transfer Catalysis – From Classical Applications to New Concepts. In Asymmetric Organocatalysis: New Strategies, Catalysts, and Opportunities; Albrecht L.; Albrecht A.; Dell’Amico L., Eds.; Wiley-VCH, 2023; pp 71–120.

[ref9] Mukherjee S.; Yang J. W.; Hoffmann S.; List B. Asymmetric Enamine Catalyis. Chem. Rev. 2007, 107, 5471–5569. 10.1021/cr0684016. 18072803

[ref10] aFu G. C. Asymmetric Catalysis with “Planar-Chiral” Derivatives of 4-(Dimethylamino)pyridine. Acc. Chem. Res. 2004, 37, 542–547. 10.1021/ar030051b. 15311953

[ref11] aTaylor J. E.; Bull S. D.; Williams J. M. J. Amidines, isothioureas, and guanidines as nucleophilic catalysts. Chem. Soc. Rev. 2012, 41, 2109–2121. 10.1039/c2cs15288f. 22234578

[ref12] Nimmo A. J.; Young C. M.; Smith A. D. Isothiourea Catalysis – New Opportunities for Asymmetric Synthesis. In Asymmetric Organocatalysis: New Strategies, Catalysts, and Opportunities; Albrecht L.; Albrecht A.; Dell’Amico L., Eds.; Wiley-VCH, 2023; pp 151–202.

[ref13] Hartley W. C.; O’Riordan T. J. C.; Smith A. D. Aryloxide-Promoted Catalyst Turnover in Lewis Base Organocatalysis. Synthesis 2017, 49, 3303–3310. 10.1055/s-0036-1589047.

[ref14] aBirman V. B.; Li X. Benzotetramisole: A Remarkably Enantioselective Acyl Transfer Catalyst. Org. Lett. 2006, 8, 1351–1354. 10.1021/ol060065s. 16562889

[ref15] Maji B.; Joannesse C.; Nigst T. A.; Smith A. D.; Mayr H. Nucleophilicities and Lewis Basicities of Isothiourea Derivatives. J. Org. Chem. 2011, 76, 5104–5112. 10.1021/jo200803x. 21568333

[ref16] aWack H.; Taggi A. E.; Hafez A. M.; Drury W. J.; Lectka T. Catalytic, Asymmetric α-Halogenation. J. Am. Chem. Soc. 2001, 123, 1531–1532. 10.1021/ja005791j. 11456741

[ref17] aPaull D. H.; Scerba M. T.; Alden-Danforth E.; Widger L. R.; Lectka T. Catalytic, Asymmetric α-Fluorination of Acid Chlorides: Dual Metal-Ketene Enolate Activation. J. Am. Chem. Soc. 2008, 130, 17260–17261. 10.1021/ja807792c. 19049284 PMC2651145

[ref18] aLee E. C.; McCauley K. M.; Fu G. C. Catalytic Asymmetric Synthesis of Tertiary Alkyl Chlorides. Angew. Chem., Int. Ed. 2007, 46, 977–979. 10.1002/anie.200604312. 17211906

[ref19] Douglas J.; Ling K. B.; Concellon C.; Churchill G.; Slawin A. M. Z.; Smith A. D. NHC-Mediated Chlorination of Unsymmetrical Ketenes: Catalysis and Asymmetry. Eur. J. Org. Chem. 2010, 2010, 5863–5869. 10.1002/ejoc.201000864.

[ref20] Yuan S.; Liao C.; Zheng W.-H. [2.2]Paracyclophane-Based Isothiourea-Catalyzed Highly Enantioselective α-Fluorination of Carboxylic Acids. Org. Lett. 2021, 23, 4142–4146. 10.1021/acs.orglett.1c01046. 33988375

[ref21] Stockhammer L.; Weinzierl D.; Bögl T.; Waser M. Enantioselective α-Chlorination Reactions of in situ Generated C1 Ammonium Enolates under Base-Free Conditions. Org. Lett. 2021, 23, 6143–6147. 10.1021/acs.orglett.1c02256. 34319102 PMC8353620

[ref22] aTidwell T. T. Ketenes, 2nd ed.; John Wiley & Sons, 2006.

[ref23] aYe T.; McKervey A. Organic Synthesis with α-Diazocarbonyl Compounds. Chem. Rev. 1994, 94, 1091–1160. 10.1021/cr00028a010. 26284754

[ref24] aTaggi A. E.; Hafez A. M.; Wack H.; Young B.; Ferraris D.; Lectka T. The Development of the First Catalyzed Reaction of Ketenes and Imines: Catalytic, Asymmetric Synthesis of β-Lactams. J. Am. Chem. Soc. 2002, 124, 6626–6635. 10.1021/ja0258226. 12047183

[ref25] aFan T.; Zhang Z.-J.; Zhang Y.-C.; Song J. Org. Lett. 2019, 21, 7897–7901. 10.1021/acs.orglett.9b02892. 31525932

[ref26] aHaughton L.; Williams J. M. J. Enzymatic Hydrolysis and Selective Racemisation Reactions of α-Chloro Esters. Synthesis 2001, 2001, 943–946. 10.1055/s-2001-13395.

[ref27] Nishimura K.; Wang Y.; Ogura Y.; Kumagai J.; Ishihara K. A π–Cu(II)−π Complex as an Extremely Active Catalyst for Enantioselective α-Halogenation of *N*-Acyl-3,5-dimethylpyrazoles. ACS Catal. 2022, 12, 1012–1017. 10.1021/acscatal.1c05500.

[ref28] Further details can be found in the online Supporting Information.

[ref29] Saeed A.; Shahzad D.; Faisal M.; Larik F. A.; El-Seedi H. R.; Channar P. A. Developments in the synthesis of the antiplatelet and antithrombotic drug (S)-clopidogrel. Chirality 2017, 29, 684–707. 10.1002/chir.22742. 28875522

