# Supplementary material for: Photochemical Wolff Rearrangement Initiated Generation and Subsequent α-Chlorination of C1 Ammonium Enolates
Source: Org Lett. 2023 Apr 25;25(17):3126–30. doi: 10.1021/acs.orglett.3c00986 (PMC10167681; doi:10.1021/acs.orglett.3c00986)
Supplement: Supplementary file 1 — ol3c00986_si_001.pdf [file ol3c00986_si_001.pdf]

SUPPORTING INFORMATION

Photochemical Wolff Rearrangement Initiated Generation and Subsequent α-Chlorination of C1 Ammonium Enolates

David Weinzierl[a], Magdalena Piringer[a], Paul Zebrowski[a], Lotte Stockhammer[a], Mario Waser[a,*]

[a]*Institute of Organic Chemistry, Johannes Kepler University Linz, Altenbergerstr. 69, 4040 Linz, Austria*

*Email: mario.waser@jku.at

Contents

1. General Information S-2
2. Photoreactor Setup S-3
3. α-Chlorination/Bromination of Activated Aryl Esters 1 .... S-4
   3.1. Preparation of α-Diazoketones S-4
   3.2. Photochemical chlorination/bromination reaction S-7
   3.3. Characterization data of α-chlorinated/brominated esters S-9
4. Substitution reaction of α-chlorinated esters S-15
5. References S-17
6. NMR Spectra of New Compounds and Selected Spectra of Literature Known Derivatives S-19
7. Copies of HPLC Chromatograms: S-45

1. General Information

^{1}H- and ^{13}C-NMR spectra were recorded on a Bruker Avance III 300 MHz spectrometer with a broad band observe probe and a sample changer for 16 samples and on a Bruker Avance DRX 500 MHz spectrometer which are both property of the Austro Czech NMR Research Center "RERI uasb". The measurements were referenced on the solvent residual peak ($CDCl_3$: δ 7.26 ppm for ^{1}H-NMR and δ 77.16 ppm for ^{13}C-NMR). NMR data are reported as follows: chemical shift (δ ppm), multiplicity (s = singlet, d = doublet, t = triplet, q = quartet, m = multiplet, br = broad), coupling constants (Hz) and integrals. High resolution mass spectra were obtained using an Agilent QTOF 6520 with ESI source. EI-MS analysis was done with a Shimadzu GC-MS QP-2020 using He as carrier gas. Optical rotations were measured on a Schmidt+Haensch Unipol L 100 polarimeter ([α]D values are listed in $deg \cdot cm^3 \cdot g^{-1} \cdot dm^{-1}$; concentration c is given in g/100 mL). Preparative column chromatography was carried out using Davisil LC 60A 70– 200 MICRON silica gel. Thin layer chromatography was performed on Macherey-Nagel pre-coated TLC plates (silica gel, 60 F254, 0.20 mm, ALUGRAM® Xtra SIL). TLC plates were visualized under 254 nm UV lamp. Enantiomeric ratios (e.r.) were determined by HPLC analysis using a Dionex Summit HPLC system with a CHIRALPAK AD-H, OD-H, CHIRAL ART Amylose-SA, Cellulose-SB, or Cellulose-SZ (250 × 4.6 mm, 5 µm) chiral stationary phase.

All chemicals were purchased from commercial suppliers and used without further purification unless otherwise stated.

As we already reported previously[1], ionization of the (mostly literature known) chlorinated esters **2** could not be achieved employing an ESI source and thus high resolution mass spectrometry could not be carried with our equipment. On this account, low resolution EI ionization was performed to confirm product formation of the methyl esters via mass spectrometry. The strategy, where HRMS appropriate derivatives could be obtained by quenching the reaction with morpholine to form the corresponding morpholine amides, which was carried out in our previous report[1], proofed to be not feasible for the reaction reported in this paper (please also refer to the results given in Scheme 2 of the main manuscript for product **2q**) as the amine directly traps the ketene (preventing any catalyst addition – chlorination) and thus only gives non-chlorinated phenyl acetic acid-based amides.

2. Photoreactor Setup

Photochemical reactions were carried out using a photoreactor equipped with 6x5W blue LEDs (Emission maximum: 445 – 450 nm, as seen in ***Figure S-2***) which was built according to literature[2] (Dimensions: 8.9 cm × 8.9 cm × 9.6 cm). Regular screw cap vials (2 mL) were used as reaction vessels except for bigger scales (1 mmol) or lower temperature reactions (< 0°C) where a Schlenk flask was used instead. Cooling was achieved by submerging the reaction flask into ice water or an Acetone/liquid Nitrogen bath to roughly half of its height. The temperature was measured in a dummy vial via an electric thermometer. Without cooling the reaction vessel reached temperatures up to 40°C.

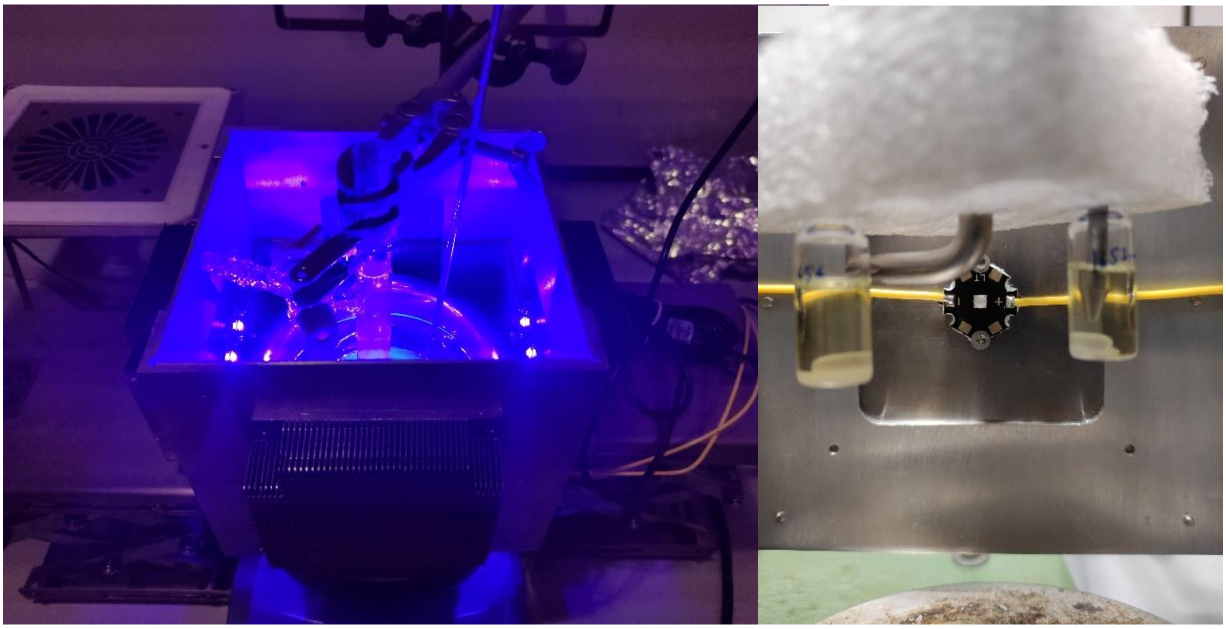

Figure S-1: Setup of the photoreactor (6x5W blue LEDs)

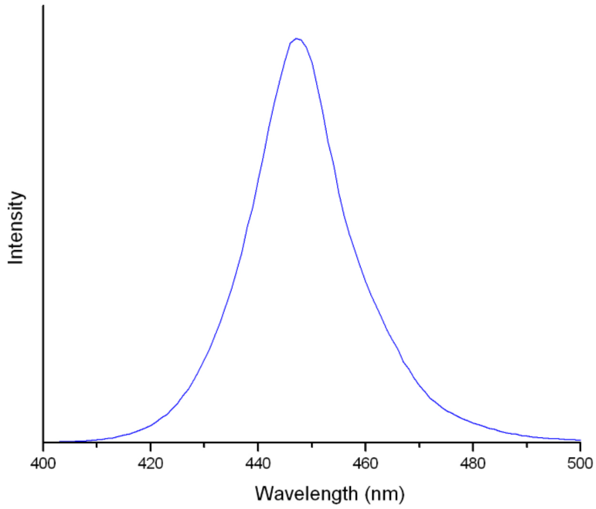

Figure S-2: Emission spectrum of the blue LED light source

3. α-Chlorination/Bromination of Activated Aryl Esters 1

3.1. Preparation of α-Diazoketones

CAUTION: Diazo compounds are toxic and potentially explosive and should be handled with care in a well-ventilated fume hood[3].

Preparation of diazoacetophenone (1a)

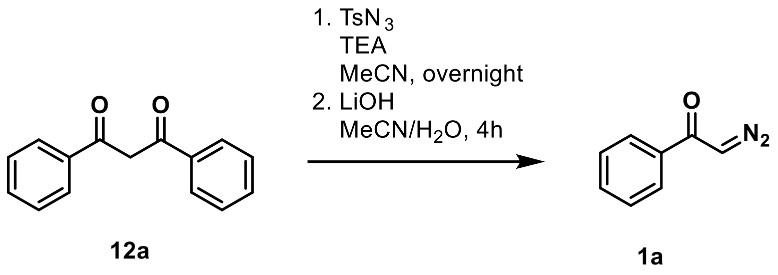

Diazoacetophenone (**1a)** was prepared according to literature[4] via the adapted procedure described below. To a solution of 1,3-diphenylpropanedione **12a** (3.0 g, 13.4 mmol) and Et_3N (2.0 mL, 14.1 mmol, 1.05 eq) in MeCN (40 mL), a solution of TsN_3 (3.3 g, 17.4 mmol, 1.25 eq) in 40 mL MeCN was added slowly and continued stirring at room temperature overnight. The intermediate diazocompound was not isolated und used directly without purification. Therefore, approximately 50% of the overall MeCN volume was distilled off on the rotary evaporator and subsequently aqueous LiOH solution (0.8 g, 33 mmol, 2.5 eq LiOH in 40 mL water) was added and stirred at room temperature for 4 h. MeCN was then fully removed on the rotary evaporator and the product was extracted with DCM and dried over Na_2SO_4. The compound was purified by column chromatography (silica gel) using Heptanes/DCM 1:1 to give **1a** as a yellow solid (yield: 80%). **^{1}H NMR** (300 MHz, Chloroform-*d*) δ 7.76 (d, *J* = 6.7 Hz, 2H), 7.55 (t, *J* = 6.7 Hz, 1H), 7.45 (t, *J* = 7.3 Hz, 2H), 5.90 (s, 1H).

Preparation of diazoacetophenone derivatives (1b-m)

Important Safety Note: *Diazomethane is potentially explosive, cancerogenic and toxic. Therefore, preparation and handling of diazomethane should be done in a well-ventilated fume hood, using an additional blast shield. The use of glass apparatus with ground joints and sharp surfaces should be avoided and Pasteur pipettes should be smoothed under a flame.*

Diazoacetophenone derivatives **1b-m** were prepared according to a procedure developed by De Kimpe et al.[5] where CaO is used as an acid scavenger in order to decrease to necessary amount of diazomethane. For the generation of diazomethane *N*-Nitroso-*N*-methylurea **12b** was used, which was prepared according to literature[6].

General Procedure for the Synthesis of α-Diazoketones

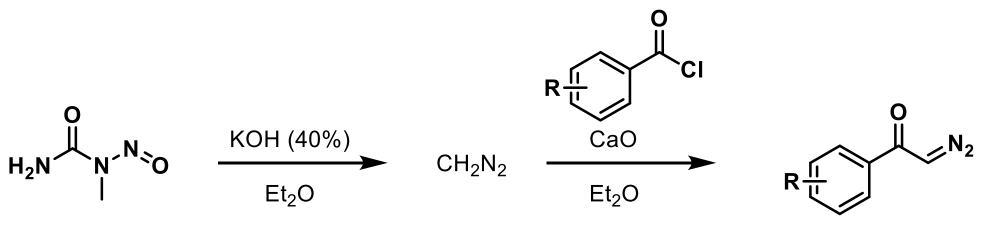

In a 40 mL test tube, 2 mL of KOH (40%) and 4 mL Et_2O were cooled to 0°C in an ice bath and stirred slowly and 0.3 g (2.9 mmol) *N*-Nitroso-*N*-methylurea **12b** was added in portions. After 30 min the stirring was stopped and the obtained yellow ethereal solution of diazomethane is separated by carefully transferring the ether phase into a second 40 mL test tube using a smoothed Pasteur pipette. The solution was again cooled to 0°C and 0.18 g (3.2 mmol, 1.1 eq) of CaO is added. The corresponding benzoyl chloride derivative was dissolved in 1 mL Et_2O and added dropwise to the stirred solution upon which gas formation was observed. After 4 h the reaction mixture had lost the intense yellow color and the CaO was filtered over a crucible (por 4). Diazoacetophenone derivatives **1b-m** were purified by column chromatography (Heptanes/EtOAc: gradient 20:1 – 5:1) and obtained as pale-yellow solids. All α-diazoketones are known to literature[4] except for **1l** where the characterization data is provided below.

Characterization data of 1l

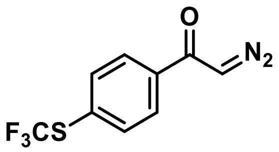

2-diazo-1-(4-((trifluoromethyl)thio)phenyl)ethan-1-one: was obtained as pale yellow solid in a yield of 65%. **M$_P$** = 90.2 - 91.9 °C. **^{1}H-NMR** (300 MHz, Chloroform-*d*) δ 7.78 (d, *J* = 8.7 Hz, 2H), 7.70 (d, *J* = 8.4 Hz, 2H), 5.94 (s, 1H). **^{19}F-NMR** (282 MHz, Chloroform-*d*) δ -41.9. **^{13}C NMR** (126 MHz, Chloroform-*d*) δ 185.1, 138.4, 136.0, 129.5 (q, *J* = 2.1 Hz), 129.4 (q, *J* = 308.4 Hz), 127.7, 55.0.

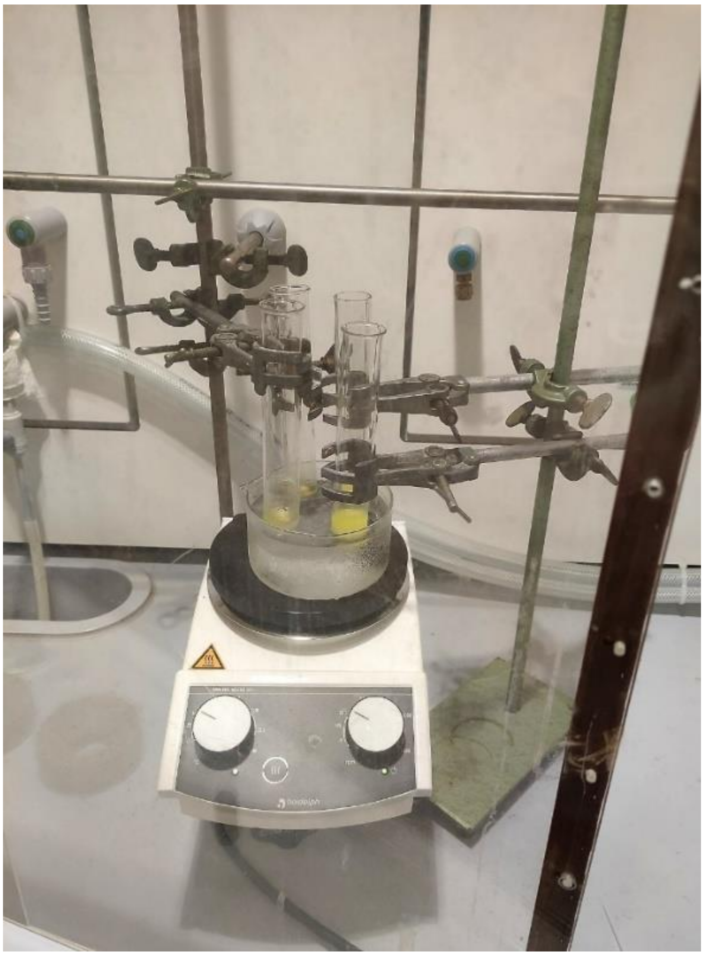

Figure S-3: Reaction setup for the generation of Diazomethane

Preparation of 1-diazo-1-phenylpropan-2-one (3)

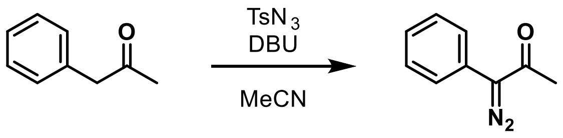

13 **3**

1-diazo-1-phenylpropan-2-one (3) was prepared according to literature[7] via the adapted procedure described below. To a solution of Phenylacetone **13** (1.0 g, 7.5 mmol) and DBU (1.5 mL, 9.7 mmol, 1.3 eq) in MeCN (20 mL), a solution of TsN_3 (1.6 g, 8.2 mmol, 1.1 eq) in 10 mL MeCN was added slowly and continued stirring at room temperature overnight. After the solvent was removed, water was added and the product was extracted with DCM and dried over Na_2SO_4. The compound was purified by column chromatography (Heptanes/EtOAc: gradient 20:1 – 5:1) to give **3** as an orange oil (yield: 832 mg, 70%). The product has to be stored in the dark at 0°C**.** ^{1}**H NMR** (300 MHz, Chloroform-*d*) δ 7.53 – 7.45 (m, 2H), 7.45 – 7.38 (m, 2H), 7.28 – 7.23 (m, 1H), 2.36 (s, 3H).

3.2. Photochemical chlorination/bromination reaction

Preparation of bromination reagent 5

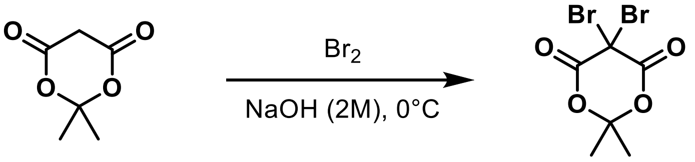

12c **5**

5,5-Dibromo-2,2-dimethyl-1,3-dioxane-4,6-dione (5): In analogy to literature[8], a mixture of 1.5 g (13 mmol) Meldrum's acid **12c** in 10 mL NaOH (2M) was cooled to 0°C in an ice bath and 1.2 mL (26 mmol, 4.2 g, 2 eq) bromine were added dropwise. After a reaction time of 2h, an orange precipitate had formed. The desired product can be obtained via two different work-up procedures yielding the product in two different appearances. Variant a: The orange precipitate is filtered, washed with water and dried in a desiccator over silica gel overnight to yield **5** as an orange powder. Variant b: The whole reaction mixture is extracted with DCM and dried over Na_2SO_4 to obtain **5** as a clear oil. (2.1 g, 67%). Both products give identical proton NMR and show the same reactivity in the bromination reaction. ^{1}**H-NMR** (300 MHz, Chloroform-*d*) δ 1.87 (s, 6H).

General Procedure for the α-chlorination/bromination

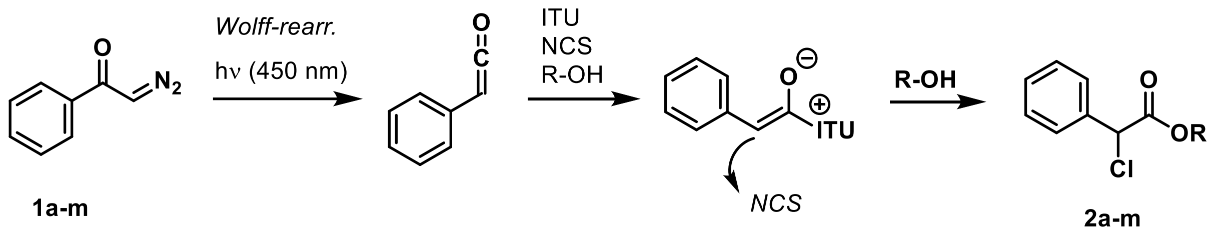

In a 2 mL screwcap vial, the corresponding α-diazocompound **1a-m** (0.1 mmol) as well as the catalyst *(2R)*-BTM **ITU3** (5.2 mg, 0.02 mmol, 20 mol%) and *N*-Chlorosuccinimide (NCS) (13.7 mg, 0.1 mmol, 1eq) for the chlorination or dibromo Meldrum's acid **5** (0.1 mmol, 1eq) for the bromination were dissolved in 1 mL dry Toluene (0.1 M) and 2 equivalents of alcohol added. The vial was submerged halfway into an ice bath and irradiated with blue LEDs (6x5 W, 450nm) for 2 h. After the reaction was finished, the solvent was removed and the product was purified by column chromatography (Heptanes/EtOAc: gradient 20:1 – 5:1).

1 mmol scale procedure for the synthesis of 2a

Diazoacetophenone **1a** (150 mg, 1 mmol) as well as the catalyst *(2R)*-BTM **ITU3** (52 mg, 0.2 mmol, 20 mol%) and *N*-Chlorosuccinimide (NCS) (137 mg, 1 mmol, 1 eq) were dissolved in 10 mL dry Toluene (0.1 M) in a 50 mL Schlenk flask and 83 µL MeOH (2 mmol, 2 eq) were added. The flask was submerged halfway into an ice bath and irradiated with blue LEDs (6x5 W, 450nm) for 2 h. After the reaction was finished, the solvent was removed and the product was purified by column chromatography (Heptanes/EtOAc: gradient 20:1 – 5:1) to afford **2a** as clear oil (136 mg, 72%, ***e.r.*** = 96:4).

Procedure for the Synthesis of α-chlorinated phenylacetic acid esters using H_2O as nucleophile

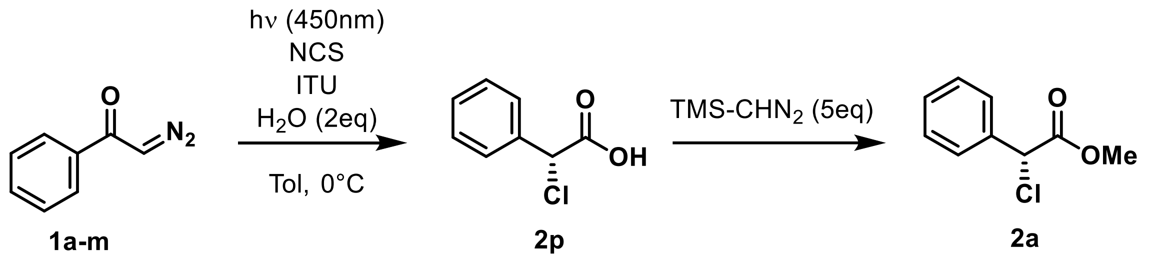

The reaction was carried out analogous to the general procedure described above, where instead of MeOH, 2 equivalents of water were added as the nucleophile. After 2h, the reaction had finished and the reaction vessel was removed from the photoreactor. In order to carry out HPLC analysis the crude mixture of acid **2p** (which is a commercial compound: CAS 4755-72-0) was esterified by adding 5 equivalents of Trimethylsilyldiazomethane (TMS-CHN_2, 2.2 M in hexanes) and the reaction continued to stir for 2h at room temperature without blue light. Subsequently, the solvent was removed and **2a** was purified by column chromatography (Heptanes/EtOAc: gradient 20:1 – 5:1).

3.3. Characterization data of α-chlorinated/brominated esters

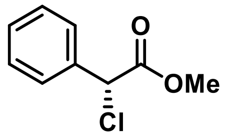

Methyl 2-chloro-2-phenylacetate[1] **(2a)** was obtained as colourless oil in a yield of 79% (14.6 mg) via the standard procedure using 0.1 mmol (14.6 mg) starting material. ***e.r.*** = 97:3. TLC (Heptanes/EtOAc = 10:1): $\boldsymbol{R_f}$ = 0.29 (UV), $[\alpha]_D^{20}$= -61.6 (*c* 1.0, $CHCl_3$, 97:3 *e.r.*), ^{1}**H-NMR** (300 MHz, Chloroform-*d*) δ 7.54 – 7.46 (m, 2H), 7.43 – 7.35 (m, 3H), 5.37 (s, 1H), 3.77 (s, 3H). ^{13}**C-NMR** (75 MHz, Chloroform-*d*) δ 169.0, 135.9, 129.5, 129.0 (2 C), 128.1 (2 C), 59.1, 53.5. **EI-MS**: *m/z* calculated for $C_9H_9ClO_2^{+\cdot}$ 184; Found 184 (15, $M^{+\cdot}$), 125 (100, $[M – COOMe]^+$). *As discussed in the general information section, the formed methyl esters were not directly detectable by ESI-TOF HRMS* **HPLC** (YMC-SB, n-hexane/IPA = 20/1, flow rate = 0.5 mL/min, l = 220 nm): t_R = 11.6 min (major), 12.4 min (minor).

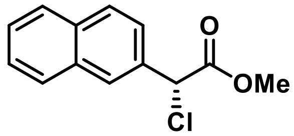

Methyl 2-chloro-2-(naphthalen-2-yl)acetate[a] **(2b)** was obtained as colourless oil in a yield of 72% (16.8 mg) via the standard procedure using 0.1 mmol (19.6 mg) starting material. ***e.r.*** = 95:5. TLC (Heptanes/EtOAc = 10:1): $\boldsymbol{R_f}$ = 0.35 (UV), $[\alpha]_D^{20}$= -64.5 (*c* 1.0, $CHCl_3$, 95:5 *e.r.*), ^{1}**H NMR** (300 MHz, Chloroform-*d*) δ 7.93 (d, *J* = 2.0 Hz, 1H), 7.90 – 7.82 (m, 3H), 7.62 (dd, *J* = 8.6, 2.0 Hz, 1H), 7.56 – 7.48 (m, 2H), 5.54 (s, 1H), 3.79 (s, 3H). ^{13}**C NMR** (75 MHz, Chloroform-*d*) δ 168.9, 133.5, 133.0, 129.0, 128.2, 127.8, 127.6, 127.3, 127.0, 126.7, 124.9, 59.3, 53.4. **EI-MS**: *m/z* calculated for $C_{13}H_{11}ClO_2^{+\cdot}$ 234; Found 234 (23, $M^{+\cdot}$), 175 (100, $[M – COOMe]^+$). **HPLC** (YMC-SA, n-hexane/IPA = 10/1, flow rate = 0.5 mL/min, l = 220 nm): tr = 11.8 min (major), 12.3 min (minor).

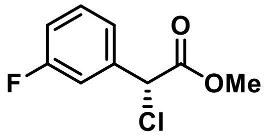

Methyl 2-chloro-2-(3-fluorophenyl)acetate[b] **(2c)** was obtained as colourless oil in a yield of 68% (13.8 mg) via the standard procedure using 0.1 mmol (16.4 mg) starting material. ***e.r.*** = 97:3. TLC (Heptanes/EtOAc = 10:1): $\boldsymbol{R_f}$ = 0.30 (UV), $[\alpha]_D^{20}$= -64.5 (*c* 1.0, $CHCl_3$, 97:3 *e.r.*), ^{1}**H NMR** (300 MHz,

[a] according to Scifinder no literature precedencies exactly for this compound could be found but the compound is available from different commercial suppliers: CAS 1249178-76-4

[b] according to Scifinder no literature precedencies exactly for this compound could be found but the compound is available from different commercial suppliers: CAS 1249616-03-2

Chloroform-*d*) δ 7.42 – 7.30 (m, 1H), 7.30 – 7.19 (m, 2H), 7.07 (tdd, *J* = 8.3, 2.5, 1.2 Hz, 1H), 5.34 (s, 1H), 3.79 (s, 3H). **^{13}C NMR** (75 MHz, Chloroform-*d*) δ 168.5, 162.9 (d, *J* = 247.4 Hz), 138.0 (d, *J* = 7.8 Hz), 130.6 (d, *J* = 8.2 Hz), 123.8 (d, *J* = 3.1 Hz), 116.6 (d, *J* = 21.1 Hz), 115.3 (d, *J* = 23.1 Hz), 58.2 (d, *J* = 2.2 Hz), 53.6. **^{19}F NMR** (282 MHz, Chloroform-*d*) δ -111.6. **EI-MS**: m/z calculated for $C_9H_8ClFO_2^{+\cdot}$ 202; Found 202 (13, $M^{+\cdot}$), 143 (100, $[M – COOMe]^+$). **HPLC** (CHIRALCEL OD-H, n-hexane/IPA = 20/1, flow rate = 0.5 mL/min, l = 220 nm): t_R = 12.5 min (major), 13.0 min (minor).

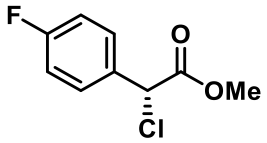

Methyl 2-chloro-2-(4-fluorophenyl)acetate[1] (2d) was obtained as colourless oil in a yield of 82% (16.6 mg) via the standard procedure using 0.1 mmol (16.4 mg) starting material. ***e.r.*** = 97:3. TLC (Heptanes/EtOAc = 10:1): ***R$_f$*** = 0.29 (UV), $[\alpha]_D^{20}$= -64.5 (*c* 1.0, $CHCl_3$, 97:3 *e.r.*), **^{1}H NMR** (300 MHz, Chloroform-*d*) δ 7.60 – 7.38 (m, 2H), 7.14 – 6.98 (m, 2H), 5.34 (s, 1H), 3.78 (s, 3H). **^{13}C NMR** (75 MHz, Chloroform-*d*) δ 168.9, 163.3 (d, *J* = 249.2 Hz), 131.8 (d, *J* = 3.3 Hz, 2C), 130.1 (d, *J* = 8.6 Hz, 2C), 116.1 (d, *J* = 22.0 Hz), 58.2, 53.6. **^{19}F NMR** (282 MHz, Chloroform-*d*) δ -111.7. **EI-MS**: m/z calculated for $C_9H_8ClFO_2^{+\cdot}$ 202; Found 202 (11, $M^{+\cdot}$), 143 (100, $[M – COOMe]^+$). **HPLC** (CHIRALCEL AD-H, n-hexane/IPA = 80/1, flow rate = 0.5 mL/min, l = 220 nm): t_R = 14.2 min (major), 14.8 min (minor).

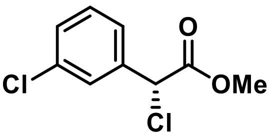

Methyl 2-chloro-2-(3-chlorophenyl)acetate[1] (2e) was obtained as colourless oil in a yield of 63% (13.8 mg) via the standard procedure using 0.1 mmol (18.1 mg) starting material. ***e.r.*** = 99:1. TLC (Heptanes/EtOAc = 10:1): ***R$_f$*** = 0.31 (UV), $[\alpha]_D^{20}$= -22.1 (*c* 1.0, $CHCl_3$, 99:1 *e.r.*), **^{1}H NMR** (300 MHz, Chloroform-*d*) δ 7.50 (t, *J* = 1.8 Hz, 1H), 7.41 – 7.28 (m, 3H), 5.31 (s, 1H), 3.79 (s, 3H). **^{13}C NMR** (75 MHz, Chloroform-*d*) δ 168.5, 137.6, 134.9, 130.3, 129.7, 128.3, 126.3, 58.2, 53.7. **EI-MS** m/z: calculated for $C_9H_8Cl_2O_2^{+\cdot}$ 218; Found 218 (17, $M^{+\cdot}$), 159 (100, $[M – COOMe]^+$). **HPLC** (YMC-SB, n-hexane/IPA = 200/1, flow rate = 0.5 mL/min, l = 220 nm): t_R = 19.0 min (major), 20.0 min (minor).

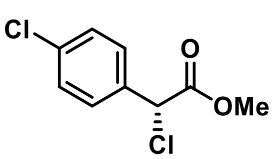

Methyl 2-chloro-2-(4-chlorophenyl)acetate[1] (2f) was obtained as colourless oil in a yield of 82% (18.0 mg) via the standard procedure using 0.1 mmol (18.1 mg) starting material. ***e.r.*** = 95:5. TLC

(Heptanes/EtOAc = 10:1): R_f = 0.29 (UV), $[\alpha]_D^{20}$= -71.4 (*c* 1.0, $CHCl_3$, 95:5 *e.r.*), ^{1}**H NMR** (300 MHz, Chloroform-*d*) δ 7.44 (d, *J* = 8.6 Hz, 2H), 7.36 (d, *J* = 8.6 Hz, 2H), 5.32 (s, 1H), 3.78 (s, 3H). ^{13}**C NMR** (75 MHz, Chloroform-*d*) δ 168.7, 135.6, 134.3, 129.5 (2C), 129.2 (2C), 58.2, 53.6. **EI-MS** m/z: calculated for $C_9H_8Cl_2O_2^{+\cdot}$ 218; Found 218 (16, $M^{+\cdot}$), 159 (100, $[M - COOMe]^+$). **HPLC** (CHIRALCEL OJ-H, n-hexane/IPA = 80/1, flow rate = 1.0 mL/min, l = 220 nm): t_R = 20.4 min (major), 22.4 min (minor).

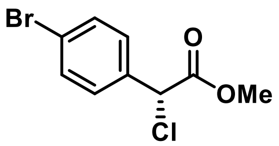

Methyl 2-chloro-2-(4-bromophenyl)acetate[1] (2g) was obtained as colourless oil in a yield of 72% (19.0 mg) via the standard procedure using 0.1 mmol (22.5 mg) starting material. ***e.r.*** = 96:4. TLC (Heptanes/EtOAc = 10:1): R_f = 0.34 (UV), $[\alpha]_D^{20}$= -52.8 (*c* 1.0, $CHCl_3$, 96:4 *e.r.*), ^{1}**H NMR** (300 MHz, Chloroform-*d*) δ 7.52 (d, *J* = 8.6 Hz, 2H), 7.37 (d, *J* = 8.4 Hz, 2H), 5.31 (s, 1H), 3.78 (s, 3H). ^{13}**C NMR** (75 MHz, Chloroform-*d*) δ 168.6, 134.9, 132.2 (2 C), 129.8 (2 C), 123.8, 58.3, 53.6. **EI-MS** m/z: calculated for $C_9H_8ClBrO_2^{+\cdot}$ 262; Found 262 (9, $M^{+\cdot}$), 203 (100, $[M - COOMe]^+$). **HPLC** (CHIRALCEL OJ-H, n-hexane/IPA = 80/1, flow rate = 1.0 mL/min, l = 240 nm): t_R = 21.7 min (major), 24.5 min (minor).

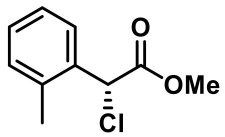

Methyl 2-chloro-2-(o-tolyl)acetate[1] (2h) was obtained as colourless oil in a yield of 68% (13.5 mg) via the standard procedure using 0.1 mmol (16.0 mg) starting material. ***e.r.*** = 83:17. TLC (Heptanes/EtOAc = 10:1): R_f = 0.35 (UV), $[\alpha]_D^{20}$= -45.4 (*c* 1.0, $CHCl_3$, 83:17 *e.r.*), ^{1}**H NMR** (300 MHz, Chloroform-*d*) δ 7.53 – 7.13 (m, 5H), 5.63 (s, 1H), 3.79 (s, 3H), 2.43 (s, 3H). ^{13}**C NMR** (75 MHz, Chloroform-*d*) δ 169.2, 136.3, 134.5, 131.0, 129.4, 128.2, 126.9, 56.4, 53.5, 19.3. **EI-MS**: m/z calculated for $C_{10}H_{11}ClO_2^{+\cdot}$ 198; Found 198 (10, $M^{+\cdot}$), 139 (100, $[M – COOMe]^+$). **HPLC** (YMC-SB, n-hexane/IPA = 250/1, flow rate = 0.5 mL/min, l = 220 nm): t_R = 20.9 min (major), 21.9 min (minor).

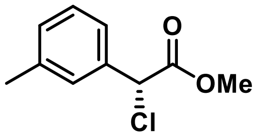

Methyl 2-chloro-2-(m-tolyl)acetate[1] (2i) was obtained as colourless oil in a yield of 70% (13.9 mg) via the standard procedure using 0.1 mmol (16.0 mg) starting material. ***e.r.*** = 97:3. TLC (Heptanes/EtOAc = 10:1): R_f = 0.34 (UV), $[\alpha]_D^{20}$= -64.8 (*c* 1.0, $CHCl_3$, 97:3 *e.r.*), ^{1}**H NMR** (300 MHz, Chloroform-*d*) δ 7.34 – 7.14 (m, 5H), 5.33 (s, 1H), 3.78 (s, 3H), 2.37 (s, 3H). ^{13}**C NMR** (75 MHz, Chloroform-*d*) δ 169.1, 138.9,

135.8, 130.3, 128.9, 128.7, 128.6, 125.1, 59.2, 53.5, 21.5.. **EI-MS**: m/z calculated for $C_{10}H_{11}ClO_2^{+\cdot}$ 198; Found 198 (10, $M^{+\cdot}$), 139 (100, $[M - COOMe]^+$). **HPLC** (YMC-SB, n-hexane/IPA = 20/1, flow rate = 0.5 mL/min, l = 220 nm): t_R = 10.8 min (major), 11.5 min (minor).

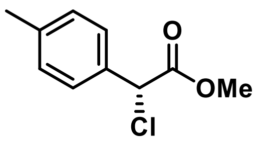

Methyl 2-chloro-2-(p-tolyl)acetate[1] (2j) was obtained as colourless oil in a yield of 62% (12.3 mg) via the standard procedure using 0.1 mmol (16.0 mg) starting material. ***e.r.*** = 88:12. TLC (Heptanes/EtOAc = 10:1): R_f = 0.35 (UV), $[\alpha]_D^{20}$ = -37.8 (*c* 1.0, $CHCl_3$, 88:12 *e.r.*), **^{1}H NMR** (300 MHz, Chloroform-*d*) δ 7.38 (d, *J* = 8.1 Hz, 2H), 7.19 (d, *J* = 7.9 Hz, 2H), 5.34 (s, 1H), 3.77 (s, 3H), 2.36 (s, 3H). **^{13}C NMR** (75 MHz, Chloroform-*d*) δ 169.1, 139.6, 133.0, 129.7 (2 C), 128.0 (2 C), 59.0, 53.4, 21.4. **EI-MS**: m/z calculated for $C_{10}H_{11}ClO_2^{+\cdot}$ 198; Found 198 (14, $M^{+\cdot}$), 139 (100, $[M - COOMe]^+$). **HPLC** (YMC-SB, n-hexane/IPA = 250/1, flow rate = 0.5 mL/min, l = 220 nm): t_R = 8.6 min (major), 9.6 min (minor).

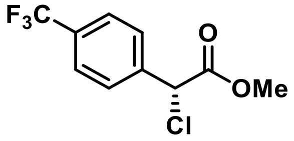

Methyl 2-chloro-2-(4-(trifluoromethyl)phenyl)acetate[c] (2k) was obtained as colourless oil in a yield of 71% (17.9 mg) via the standard procedure using 0.1 mmol (21.4 mg) starting material. ***e.r.*** = 94:6. TLC (Heptanes/EtOAc = 10:1): R_f = 0.33 (UV), $[\alpha]_D^{20}$ = -34.9 (*c* 1.0, $CHCl_3$, 94:6 *e.r.*), **^{1}H NMR** (300 MHz, Chloroform-*d*) δ 7.77 – 7.54 (m, 4H), 5.39 (s, 1H), 3.79 (s, 3H). **^{13}C NMR** (126 MHz, Chloroform-*d*) δ 168.4, 139.6, 131.6 (q, *J* = 32.7 Hz), 128.6 (2C), 126.0 (q, *J* = 3.7 Hz, 2C), 123.9 (q, *J* = 272.4 Hz), 58.2, 53.7. **^{19}F NMR** (282 MHz, Chloroform-*d*) δ -62.9. **EI-MS**: m/z calculated for $C_{10}H_8ClF_3O_2^{+\cdot}$ 252; Found 252 (9, $M^{+\cdot}$), 193 (100, $[M - COOMe]^+$). **HPLC** (YMC-SA, n-hexane/IPA = 19/1, flow rate = 0.5 mL/min, l = 220 nm): t_R = 10.0min (major), 10.7 min (minor).

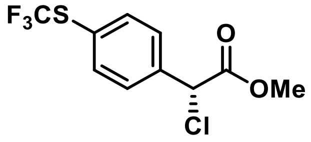

Methyl 2-chloro-2-(4-((trifluoromethyl)thio)phenyl)acetate[d] (2l) was obtained as colourless oil in a yield of 59% (16.8 mg) via the standard procedure using 0.1 mmol (24.6 mg) starting material.

[c] according to Scifinder no literature precedencies exactly for this compound could be found but the compound is available from different commercial suppliers: CAS 1250104-84-7

[d] according to Scifinder no literature precedencies exactly for this compound could be found but the compound is available from different commercial suppliers: CAS 1457016-22-6

e.r. = 97:3. TLC (Heptanes/EtOAc = 10:1): $\boldsymbol{R_f}$ = 0.35 (UV), $[\alpha]_D^{20}$= -49.4 (*c* 1.0, $CHCl_3$, 97:3 *e.r.*), ^{1}**H NMR** (300 MHz, Chloroform-*d*) δ 7.70 (d, *J* = 8.3 Hz, 2H), 7.58 (d, *J* = 8.3 Hz, 2H), 5.39 (s, 1H), 3.82 (s, 3H). ^{13}**C NMR** (126 MHz, Chloroform-d) δ 168.4, 138.6, 136.7 (2C), 129.5 (q, J = 308.2 Hz), 129.2 (2C), 125.9 (q, J = 2.0 Hz), 58.2, 53.7. ^{19}**F NMR** (282 MHz, Chloroform-*d*) δ -42.3. **EI-MS**: m/z calculated for $C_{10}H_8ClF_3O_2S^{+\cdot}$ 284; Found 284 (28, $M^{+\cdot}$), 225 (100, $[M - COOMe]^+$). **HPLC** (CHIRALCEL OD-H, n-hexane/IPA = 20/1, flow rate = 0.5 mL/min, l = 220 nm): t_R = 12.0 min (major), 12.9 min (minor).

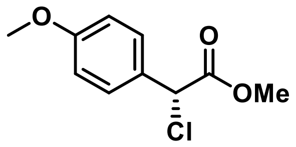

Methyl 2-chloro-2-(4-methoxyphenyl)acetate[1] (2m) was obtained as colourless oil in a yield of 66% (14.2 mg) via the standard procedure using 0.1 mmol (17.6 mg) starting material. ***e.r.*** = 94:6. TLC (Heptanes/EtOAc = 10:1): $\boldsymbol{R_f}$ = 0.35 (UV), $[\alpha]_D^{20}$= -39.8 (*c* 1.0, $CHCl_3$, 94:6 *e.r.*), ^{1}**H NMR** (300 MHz, Chloroform-*d*) δ 7.42 (d, *J* = 8.8 Hz, 2H), 6.90 (d, *J* = 8.8 Hz, 2H), 5.33 (s, 1H), 3.81 (s, 3H), 3.77 (s, 3H). ^{13}**C NMR** (75 MHz, Chloroform-*d*) δ 169.2, 160.5, 129.5 (2 C), 127.9, 114.4(2 C), 58.9, 55.5, 53.4. **EI-MS**: m/z calculated for $C_{10}H_{11}ClO_3^{+\cdot}$ 214; Found 214 (12, $M^{+\cdot}$), 155 (100, $[M - COOMe]^+$). **HPLC** (YMC-SB, n-hexane/IPA = 80/1, flow rate = 0.5 mL/min, l = 220 nm): t_R = 22.4 min (major), 23.7 min (minor).

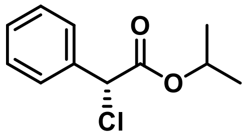

Isopropyl 2-chloro-2-phenylacetate[1] (2n) was obtained as colourless oil in a yield of 64% (13.6 mg) via the standard procedure using 0.1 mmol (14.6 mg) starting material. ***e.r.*** = 87:13. TLC (Heptanes/EtOAc = 10:1): $\boldsymbol{R_f}$ = 0.43 (UV), $[\alpha]_D^{20}$= -28.0 (*c* 1.0, $CHCl_3$, 87:13 *e.r.*), ^{1}**H NMR** (300 MHz, Chloroform-*d*) δ 7.54 – 7.45 (m, 2H), 7.41 – 7.32 (m, 3H), 5.31 (s, 1H), 5.06 (hept, *J* = 6.3 Hz, 1H), 1.27 (d, *J* = 6.3 Hz, 3H), 1.19 (d, *J* = 6.3 Hz, 3H). ^{13}**C NMR** (75 MHz, Chloroform-*d*) δ 168.0, 136.1, 129.3, 128.9 (2 C), 128.0 (2 C), 70.5, 59.5, 21.7, 21.5. **EI-MS** m/z: calculated for $C_{11}H_{13}ClO_2^{+\cdot}$: 212; Found 212 (0.3, $M^{+\cdot}$), 125 (55, $[M - COOiPr]^+$), 43 (100). **HPLC** (YMC-SB, n-hexane/IPA = 500/1, flow rate = 0.5 mL/min, l = 220 nm): t_R = 16.6 min (major), 18.0 min (minor).

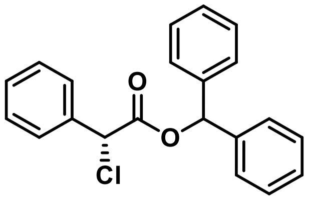

Benzhydryl 2-chloro-2-phenylacetatet (2o) was obtained as colourless oil in a yield of 45% (15.2 mg) via the standard procedure using 0.1 mmol (14.6 mg) starting material. ***e.r.*** = 90:10. TLC (Heptanes/EtOAc = 10:1): R_f = 0.43 (UV), $[\alpha]_D^{20}$= -54.5 (*c* 1.0, $CHCl_3$, 90:10 *e.r.*), ^{1}**H NMR** (300 MHz, Chloroform-*d*) δ 7.50 – 7.43 (m, 2H), 7.39 – 7.35 (m, 3H), 7.34 – 7.27 (m, 5H), 7.26 – 7.23 (m, 3H), 7.18 – 7.09 (m, 2H), 6.89 (s, 1H), 5.47 (s, 1H). ^{13}**C NMR** (75 MHz, $CDCl_3$) δ 167.3, 139.4, 139.3, 135.7, 129.4, 129.0 (2C), 128.7 (2C), 128.6 (2C), 128.4, 128.2 (2C), 128.1, 127.4 (2C), 126.8 (2C), 79.0, 59.5. **EI-MS** m/z: calculated for $C_{21}H_{17}ClO_2^{+\cdot}$: 336; Found 336 (0.7, $M^{+\cdot}$), 125 (31, [M – $COOCHPh_2$]$^+$), 28 (100). **HPLC** (YMC-SZ, n-hexane/IPA = 20/1, flow rate = 0.5 mL/min, l = 220 nm): t_R = 11.8 min (major), 12.5 min (minor).

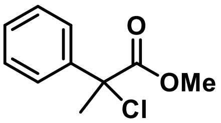

Methyl 2-chloro-2-phenylpropanoate[9] **(4)** was obtained as colourless oil in a yield of 32% (6.4 mg) via the standard procedure using 0.1 mmol (16.0 mg) starting material. ***e.r.*** < 60:40. TLC (Heptanes/EtOAc = 10:1): R_f = 0.37 (UV), ^{1}**H NMR** (300 MHz, Chloroform-*d*) δ 7.58 – 7.48 (m, 2H), 7.44 – 7.27 (m, 4H), 3.78 (s, 3H), 2.14 (s, 3H). ^{13}**C NMR** (75 MHz, $CDCl_3$) δ 171.6, 140.9, 128.7 (2C), 128.6, 126.1 (2C), 69.8, 53.7, 30.3. **EI-MS**: m/z calculated for $C_{10}H_{11}ClO_2^{+\cdot}$ 198; Found 198 (1.5, $M^{+\cdot}$), 105 (100, [M – COOMe – Cl]$^+$). **HPLC** (CHIRALCEL OD-H, n-hexane/IPA = 100/1, flow rate = 0.5 mL/min, l = 220 nm): t_R = 13.4 min (major), 13.9 min (minor).

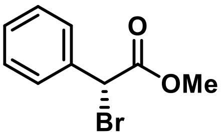

Methyl 2-bromo-2-phenylacetate[10] **(6)** was obtained as colourless oil in a yield of 35% (8.0 mg) via the standard procedure using 0.1 mmol (14.6 mg) starting material. ***e.r.*** = 60:40. TLC (Heptanes/EtOAc = 10:1): R_f = 0.33 (UV), ^{1}**H NMR** (300 MHz, Chloroform-*d*) δ 7.61 – 7.48 (m, 2H), 7.44 – 7.26 (m, 3H), 5.36 (s, 1H), 3.79 (s, 3H). ^{13}**C NMR** (75 MHz, Chloroform-*d*) δ 168.9, 135.9, 129.5, 129.0 (2C), 128.8 (2C), 53.5, 46.7. **EI-MS**: m/z calculated for $C_9H_9BrO_2^{+\cdot}$ 228; Found 228 (1.75, $M^{+\cdot}$), 169 (20, [M – COOMe]$^+$), 121 (100, [PhCHOMe] $^+$ *(MS measurement in MeOH solution: α-Br was replaced by α-OMe in the fragment [M – COOMe]$^+$)*)

4. Substitution reaction of α-chlorinated esters

Synthesis of methyl (S)-2-phenyl-2-(phenylthio)acetate (7)

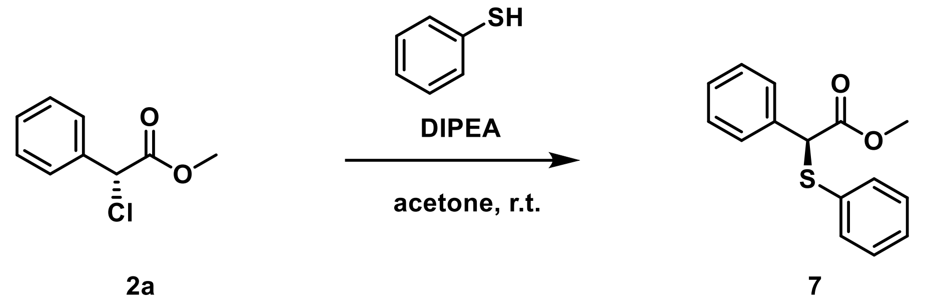

Methyl (S)-2-phenyl-2-(phenylthio)acetate (7): First, thiophenol (5.1 µL, 0.05 mmol, 1 eq.) was dissolved in 1 mL acetone at r.t. Afterwards, DIPEA (8.5 µL, 0.05 mmol, 1 eq.) was added in one portion and the solution was stirred at r.t. for 5 minutes. Subsequently, the respective ester **2a** (9.2 mg, 0.05 mmol, *e.r.* = 99:1) was added and the mixture was stirred at r.t. for further 3 h. After this, the solvent was evaporated and the crude product was purified by column chromatography (heptanes/DCM = 1:1, R_f = 0.48). The desired compound (*S*)-**7** was afforded in high yield as colorless oil (10.3 mg, 80%, *e.r.* = 98:2*, e.s.* = 98%) with analytical data fitting those reported previously[11]. **^{1}H-NMR** (300 MHz, $CDCl_3$, 298.0 K): δ / ppm = 7.37-7.18 (m, 10H Ar-**H**), 4.84 (s, 1H, -C**H**), 3.60 (s, 3H, -C**H$_3$**); **^{13}C-NMR** (75 MHz, $CDCl_3$, 298.0 K): δ / ppm = 171.0 (1C, **C**=O), 135.7 (1C, **C$_{Ar}$**), 133.8 (1C, **C$_{Ar}$**), 132.8 (2C, **C$_{Ar}$**), 129.1 (2C, **C$_{Ar}$**), 128.8 (2C, **C$_{Ar}$**), 128.6 (2C, **C$_{Ar}$**), 128.5 (1C, **C$_{Ar}$**), 128.2 (1C, **C$_{Ar}$**), 56.5 (1C, -**C**H_3), 52.9 (1C, -**C**H); **HRMS** (ESI-TOF) m/z: $[M + H]^+$ Calcd for $C_{15}H_{15}O_2S^+$ 259.0787; Found 259.0787; **HPLC** (YMC-SB, *n*-hexane/IPA = 250/1, flow rate = 1.0 mL/min, l = 220 nm): t_R = 16.8 min (major), 22.4 min (minor). $[\alpha]_D^{22}$ = +158.7 (c = 1, $CHCl_3$, *er* = 98:2).

Synthesis of methyl 2-(6,7-dihydrothieno[3,2-c]pyridin-5(4H)-yl)-2-phenylacetate (8)

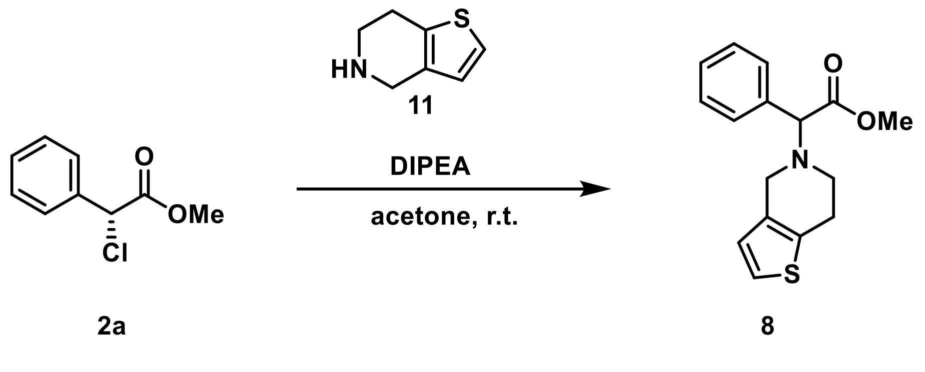

Methyl 2-(6,7-dihydrothieno[3,2-c]pyridin-5(4H)-yl)-2-phenylacetate (8): First, compound **11** (5.1 µL, 0.05 mmol, 1 eq.) was dissolved in 1 mL acetone at r.t. Afterwards, DIPEA (8.5 µL, 0.05 mmol, 1 eq.) was added in one portion and the solution was stirred at r.t. for 5 minutes. Subsequently, the respective ester **2a** (9.2 mg, 0.05 mmol, *e.r.* = 99:1) was added and the mixture was stirred at r.t. for further 24 h. After this, the solvent was evaporated and the crude product was purified by column chromatography (heptanes/EtOAc = 10:1, R_f = 0.23). The desired amine **8** was afforded in 46% (6.6 mg) yield as slightly yellowish oil (*e.r.* = 69:31*,* *e.s.* = 39%) with analytical data fitting those reported previously[12]. **^{1}H-NMR** (300 MHz, $CDCl_3$, 298.0 K): δ / ppm =7.51-7.47 (m, 2H, Ar-**H**), 7.40-7.33 (m, 3H, Ar-**H**), 7.06 (d, *J* = 5.2 Hz, 1H, Ar-**H**), 6.66 (d, *J* = 5.1 Hz, 1H, Ar-**H**), 4.31 (s, 1H, -C**H**), 3.73 (s, 3H, -C**H₃**), 3.68-3.57 (m, 2H, C**H₂**), 2.94-2.77 (m, 4H, C**H₂**); **^{13}C-NMR** (75 MHz, $CDCl_3$, 298.0 K): δ / ppm = 172.0 (1C, **C**=O), 135.7 (1C, $\mathbf{C}_{Ar}$), 133.3 (1C, $\mathbf{C}_{Ar}$), 133.1 (1C, $\mathbf{C}_{Ar}$), 129.0 (2C, $\mathbf{C}_{Ar}$), 128.9 (2C, $\mathbf{C}_{Ar}$), 128.8 (2C, $\mathbf{C}_{Ar}$), 125.4 (1C, $\mathbf{C}_{Ar}$), 123.0 (1C, $\mathbf{C}_{Ar}$), 72.7 (1C, -**C**H), 52.3 (1C, -**C**H_2), 51.0 (1C, -**C**H_3), 48.4 (1C, -**C**H_2), 25.2 (1C, -**C**H_2); **HRMS** (ESI-TOF) m/z: $[M + H]^+$ Calcd for $C_{16}H_{18}NO_2S^+$ 288.1053; Found: 288.1059; **HPLC** (YMC-SB, *n*-hexane/IPA = 200/1, flow rate = 1.0 mL/min, l = 220 nm): t_R = 13.8 min (major), 15.6 min (minor). $[\alpha]_D^{22}$ = +5.9 (c = 1, $CHCl_3$, *e.r.* = 69:31).

Synthesis of methyl (S)-2-azido-2-phenylacetate (9)

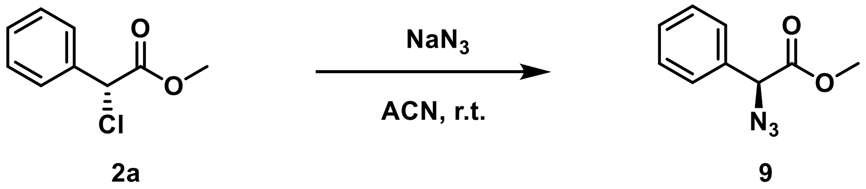

Methyl (S)-2-azido-2-phenylacetate (9): First, ester **2a** (9.2 mg, 0.05 mmol, *e.r.* = 99:1) was dissolved in 0.5 mL anhydrous ACN. Then, the first portion of NaN_3 (2.2 mg, 0.03 mmol) was added to the stirred solution in one portion. Stirring was continued for further 7 h at r.t. Afterwards, the second portion of NaN_3 (2.2 mg, 0.03 mmol) was added and stirring was again continued at r.t. The reaction was aborted after 24 h and purification by preparative TLC (heptanes/EtOAc = 10:1, R_f = 0.21) gave the desired azide (*S*)-**9** in 63% (6.0 mg) yield as a colorless oil (*e.r.* = 96:4, *e.s.* = 94%) with analytical data fitting those reported previously[13]. **^{1}H-NMR** (300 MHz, $CDCl_3$, 298.0 K): δ / ppm = 7.45-7.37 (m, 5H Ar-**H**), 4.98 (s, 1H, -C**H**), 3.78 (s, 3H, -C**H₃**); **^{13}C-NMR** (75 MHz, $CDCl_3$, 298.0 K): δ / ppm = 169.7 (1C, **C**=O), 134.0 (1C, $\mathbf{C}_{Ar}$), 129.5 (1C, $\mathbf{C}_{Ar}$), 129.3 (2C, $\mathbf{C}_{Ar}$), 127.8 (2C, $\mathbf{C}_{Ar}$), 65.5 (1C, -**C**H), 53.1 (1C, -**C**H); **HRMS** (ESI-TOF) m/z: $[M + H]^+$ Calcd for $C_9H_{10}N_3O_2^+$ 192.0768; Found 192.0765; **HPLC** (YMC-SB, *n*-hexane/IPA = 250/1, flow rate = 1.0 mL/min, l = 220 nm): t_R = 12.7 min (major), 13.5 min (minor). $[\alpha]_D^{22}$ = +94.9 (c = 1, $CHCl_3$, *e.r.* = 96:4).

Synthesis of methyl (S)-2-amino-2-phenylacetate (10)

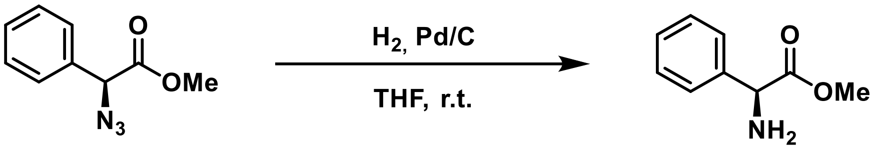

9 10

Methyl (S)-2-amino-2-phenylacetate (10): According to literature[14], ester **9** (15.0 mg, 0.078 mmol, *e.r.* = 96:4) was dissolved in 2 mL anhydrous THF. Then, Pd/C (1.5 mg, 10 wt%) was added to the stirred solution in one portion and the flask was flushed with H_2 (balloon). Stirring of the suspension was continued for further 4 h at r.t. Afterwards, the flask was flushed with argon and filtered over a Celite plug (1 cm). The desired amine **10** was obtained as colorless viscous oil in quantitative yield (12.8 mg, 0.077 mmol, *e.r.* = 96:4). ^{1}**H-NMR** (300 MHz, $CDCl_3$, 298.0 K): δ / ppm = 7.41-7.28 (m, 5H, Ar-**H**), 4.64 (s, 1H, -C**H**), 3.70 (s, 3H, -C$\mathbf{H_3}$), 3.11 (br, 2H, -N$\mathbf{H_2}$); ^{13}**C-NMR** (75 MHz, $CDCl_3$, 298.0 K): δ / ppm = 174.6 (1C, **C**=O), 140.3 (1C, $\mathbf{C_{Ar}}$), 129.0 (1C, $\mathbf{C_{Ar}}$), 128.2 (2C, $\mathbf{C_{Ar}}$), 127.0 (2C, $\mathbf{C_{Ar}}$), 58.9 (1C, -**C**H), 52.6 (1C, -**C**H); **HRMS** (ESI-TOF) m/z: $[M + H]^+$ Calcd for $C_9H_{12}NO_2^+$ 166.0863; Found: 166.0860; **HPLC** (YMC-SB, *n*-hexane/IPA = 5/1, flow rate = 1.0 mL/min, l = 220 nm): t_R = 11.5 min (major), 12.4 min (minor); $[\alpha]_D^{22}$ = +90.5 (c = 1, $CHCl_3$, *e.r.* = 96:4).

5. References

(1) Stockhammer, L.; Weinzierl, D.; Bögl, T.; Waser, M. Enantioselective α-Chlorination Reactions of in Situ Generated C1 Ammonium Enolates under Base-Free Conditions. *Organic letters* **2021**, *23* (15), 6143–6147. DOI: 10.1021/acs.orglett.1c02256

(2) Contreras-Cruz, D. A.; Cantú-Reyes, M.; García-Sánchez, J. M.; Peña-Ortíz, D.; Sánchez-Carmona, M. A.; Miranda, L. D. Shedding Blue Light on the Undergraduate Laboratory: An Easy-to-Assemble LED Photoreactor for Aromatization of a 1,4-Dihydropyridine. *J. Chem. Educ.* **2019**, *96* (9), 2015–2020. DOI: 10.1021/acs.jchemed.8b01026

(3) Green, S. P.; Wheelhouse, K. M.; Payne, A. D.; Hallett, J. P.; Miller, P. W.; Bull, J. A. Thermal Stability and Explosive Hazard Assessment of Diazo Compounds and Diazo Transfer Reagents. *Organic process research & development* **2020**, *24* (1), 67–84. DOI: 10.1021/acs.oprd.9b00422

(4) Liu, Q.; Li, M.; Xiong, R.; Mo, F. Direct Carboxylation of the Diazo Group ipso-C(sp2)-H bond with Carbon Dioxide: Access to Unsymmetrical Diazomalonates and Derivatives. *Organic letters* **2017**, *19* (24), 6756–6759. DOI: 10.1021/acs.orglett.7b03573

(5) Pace, V.; Verniest, G.; Sinisterra, J.-V.; Alcántara, A. R.; Kimpe, N. de. Improved Arndt-Eistert synthesis of alpha-diazoketones requiring minimal diazomethane in the presence of calcium oxide as acid scavenger. *The Journal of organic chemistry* **2010**, *75* (16), 5760–5763. DOI: 10.1021/jo101105g

(6) Shields, S. W. J.; Rosales, C. A.; Roberts, J. A.; Pallister, P. J.; Wasslen, K. V.; Manthorpe, J. M.; Smith, J. C. iTrEnDi: In Situ Trimethylation Enhancement Using Diazomethane: Improved and Expanded Glycerophospholipid and Sphingolipid Analyses via a Microscale Autonomous

Derivatization Platform. *Analytical chemistry* **2021**, *93* (2), 1084–1091. DOI: 10.1021/acs.analchem.0c04088

(7) Xu, B.; Zhu, S.-F.; Zuo, X.-D.; Zhang, Z.-C.; Zhou, Q.-L. Enantioselective N-H insertion reaction of α-aryl α-diazoketones: an efficient route to chiral α-aminoketones. *Angewandte Chemie (International ed. in English)* **2014**, *53* (15), 3913–3916. DOI: 10.1002/anie.201400236

(8) Kuhn, N.; Al-Sheikh, A.; Schwarz, S.; Steimann, M. Zur Reaktion von 2,3-Dihydro-1,3-diisopropy1-4,5-imidazol-2-yliden mit Bromderivaten der Meldrumsäure [1, 2] / On the Reaction of 2,3-Dihydro-1,3-diisopropy1-4,5-dimethylimidazol-2-ylidene with Bromine Derivatives of Meldrum's Acid [1, 2]. *Zeitschrift für Naturforschung B* **2004**, *59* (2), 129–133. DOI: 10.1515/znb-2004-0202

(9) Pitta, B. R.; Fleming, F. F. Metalated nitrile and enolate chlorinations. *Organic letters* **2010**, *12* (12), 2810–2813. DOI: 10.1021/ol100897y

(10) Tayama, E.; Sato, R.; Takedachi, K.; Iwamoto, H.; Hasegawa, E. A formal method for the de-N,N-dialkylation of Sommelet–Hauser rearrangement products. *Tetrahedron* **2012**, *68* (24), 4710–4718. DOI: 10.1016/j.tet.2012.04.015

(11) Nakamura, S.; Nakagawa, R.; Watanabe, Y.; Toru, T. Highly Enantioselective Reactions of Configurationally Labile α-Thioorganolithiums Using Chiral Bis(oxazoline)s via Two Different Enantiodetermining Steps. *J. Am. Chem. Soc.* **2000**, *122* (46), 11340–11347. DOI: 10.1021/ja0025191

(12) Arredondo, V.; Hiew, S. C.; Gutman, E. S.; Premachandra, I. D. U. A.; van Vranken, D. L. Enantioselective Palladium-Catalyzed Carbene Insertion into the N-H Bonds of Aromatic Heterocycles. *Angewandte Chemie (International ed. in English)* **2017**, *56* (15), 4156–4159. DOI: 10.1002/anie.201611845

(13) a) Paul, A.; Bittermann, H.; Gmeiner, P. Triazolopeptides: chirospecific synthesis and cis/trans prolyl ratios of structural isomers. *Tetrahedron* **2006**, *62* (38), 8919–8927. DOI: 10.1016/j.tet.2006.07.007#; b) Maurice Caron/Paul R. Carlier/K. Barry Sharpless. Regioselective azide opening of 2,3-epoxy alcohols by [Ti(O-i-Pr)2(N3)2]: synthesis of .alpha.-amino acids;

(14) Ishihara, K.; Hamamoto, H.; Matsugi, M.; Shioiri, T. SN2 displacement at the quaternary carbon center: a novel entry to the synthesis of α,α-disubstituted α-amino acids. *Tetrahedron Letters* **2015**, *56* (23), 3169–3171. DOI: 10.1016/j.tetlet.2015.01.041

6. NMR Spectra of New Compounds and Selected Spectra of Literature Known Derivatives

^{1}H-NMR of **1l** (300 MHz, $CDCl_3$, 298 K):

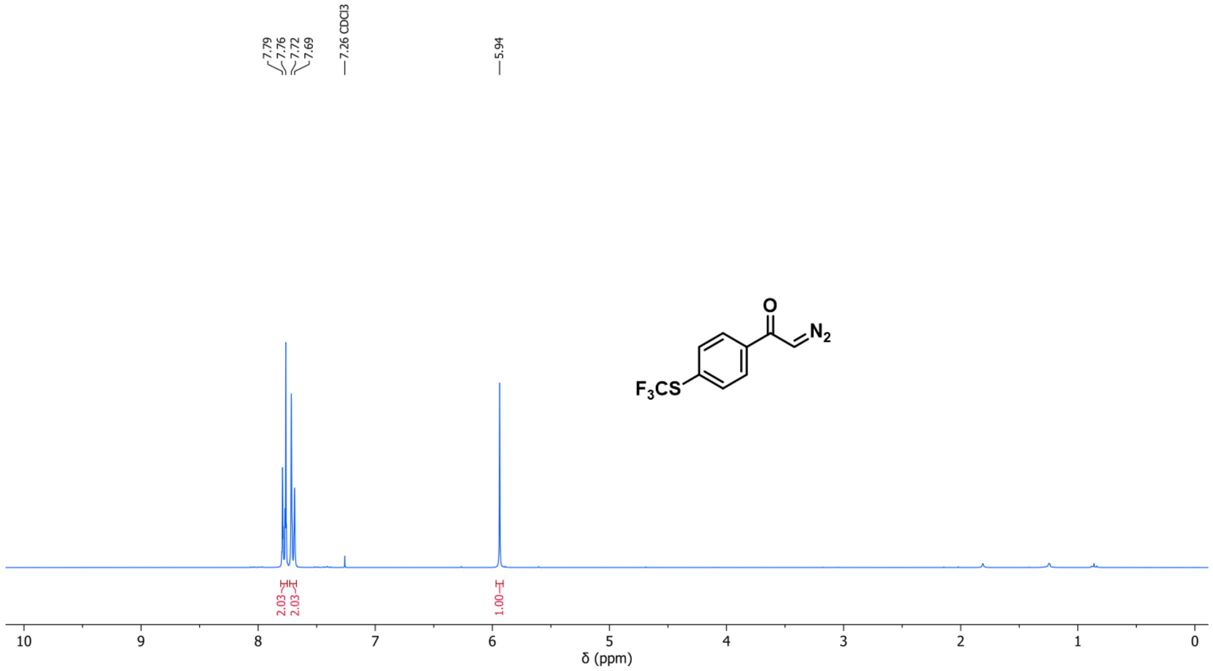

^{19}F-NMR of **1l** (282 MHz, $CDCl_3$, 298 K):

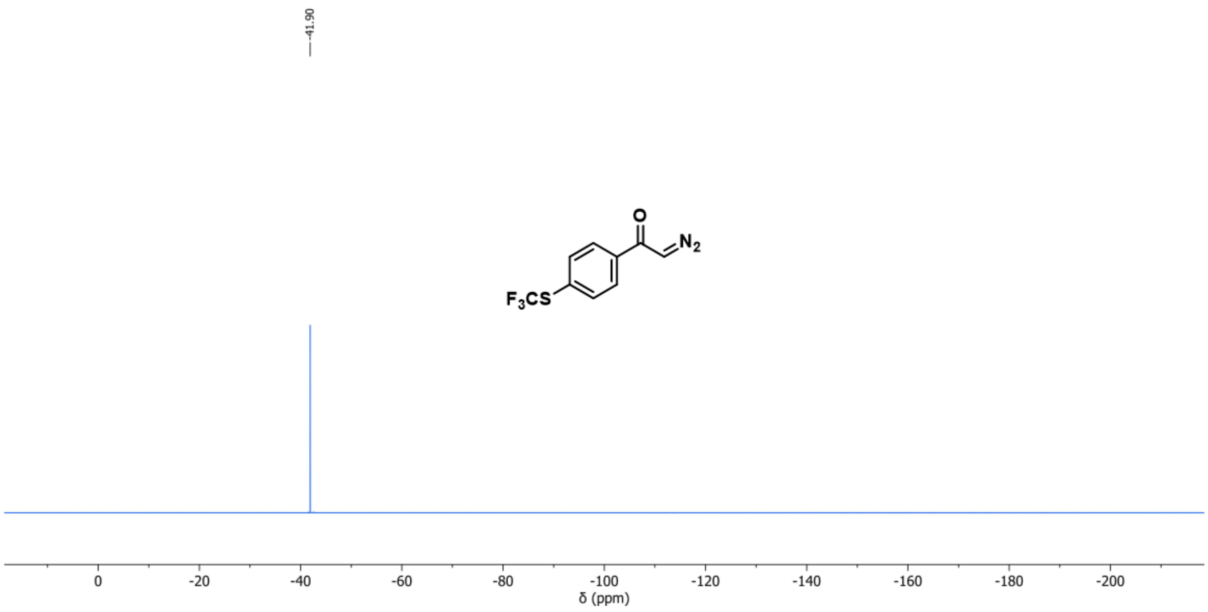

^{13}C-NMR of **1l** (75 MHz, $CDCl_3$, 298 K):

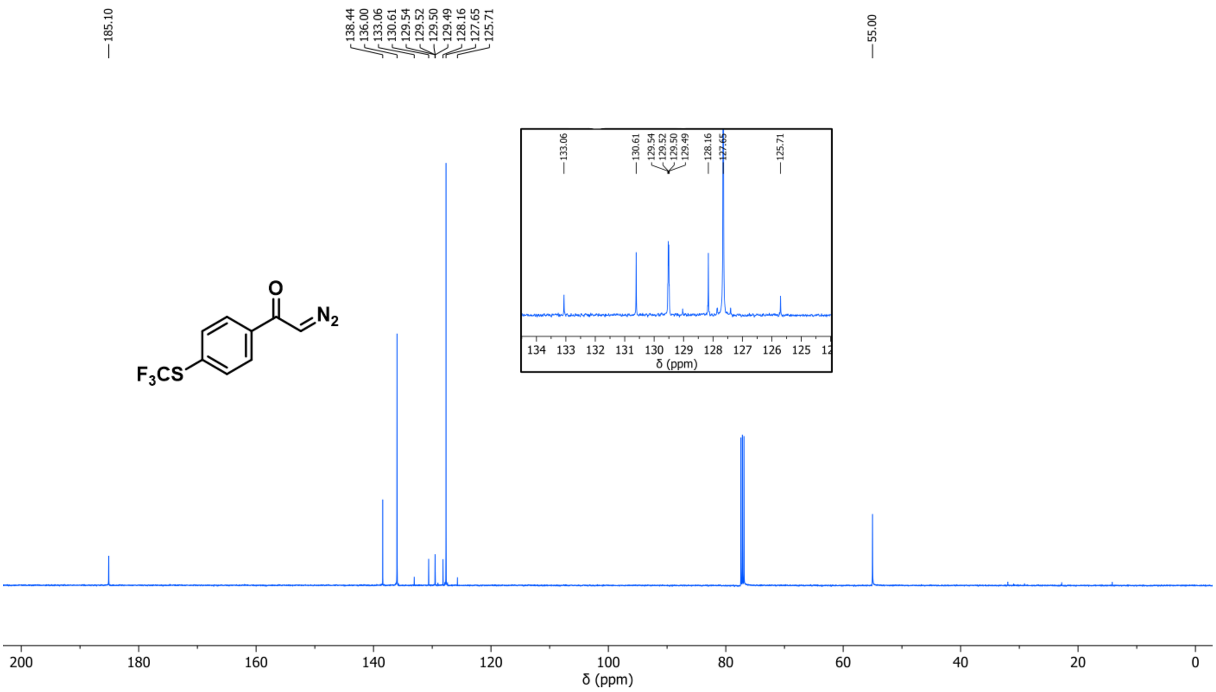

^{1}H-NMR of **3** (300 MHz, $CDCl_3$, 298 K):

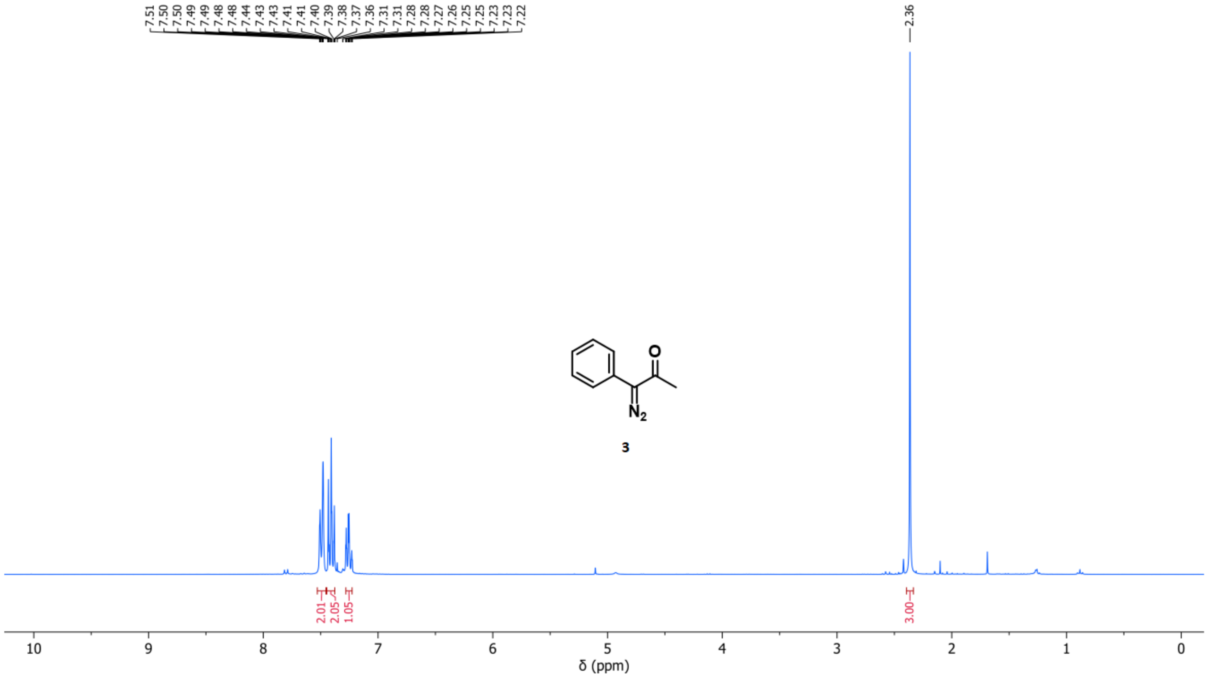

^{1}H-NMR of **5** (300 MHz, $CDCl_3$, 298 K):

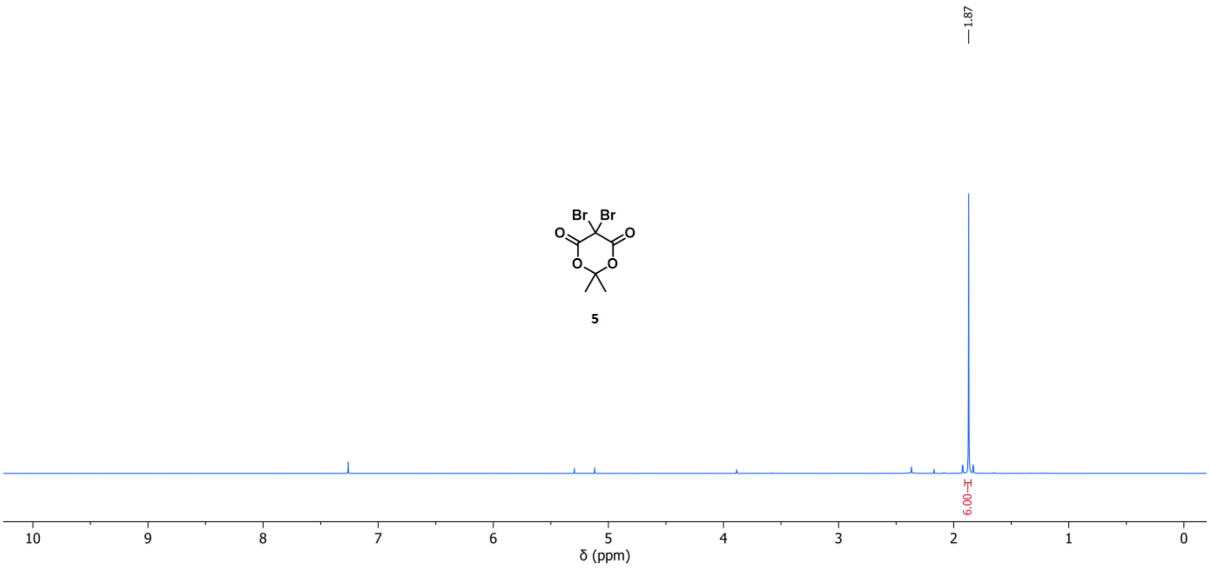

^{1}H-NMR of **2a** (300 MHz, $CDCl_3$, 298 K):

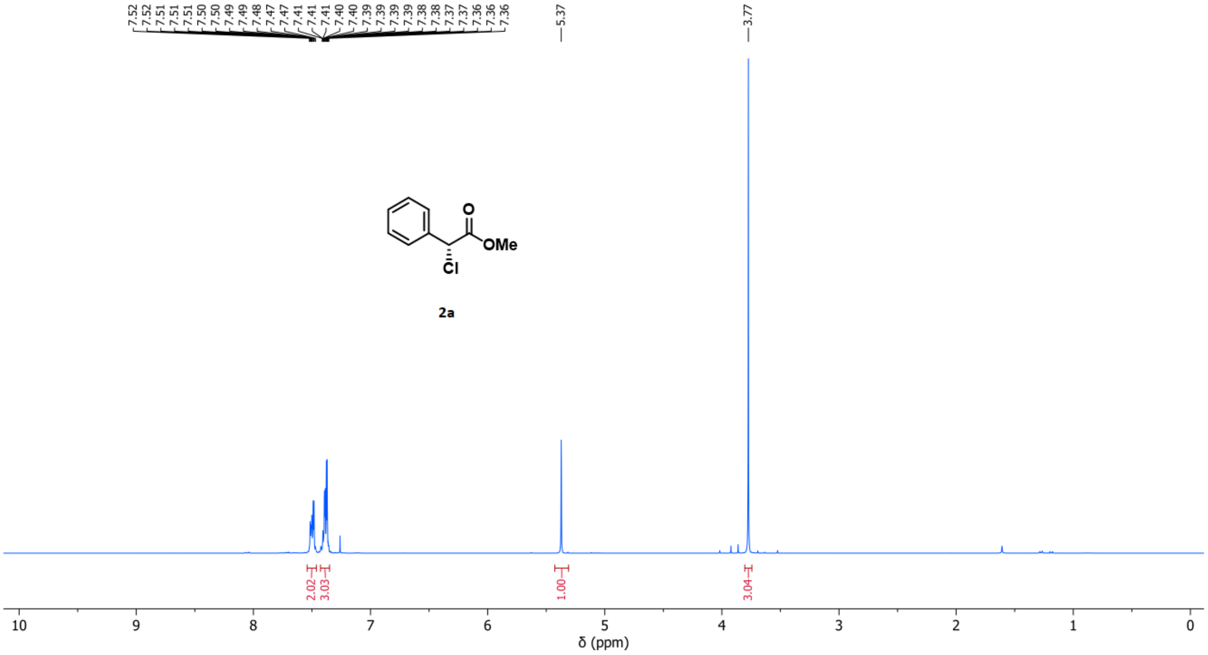

^{13}C-NMR of **2a** (75 MHz, $CDCl_3$, 298 K):

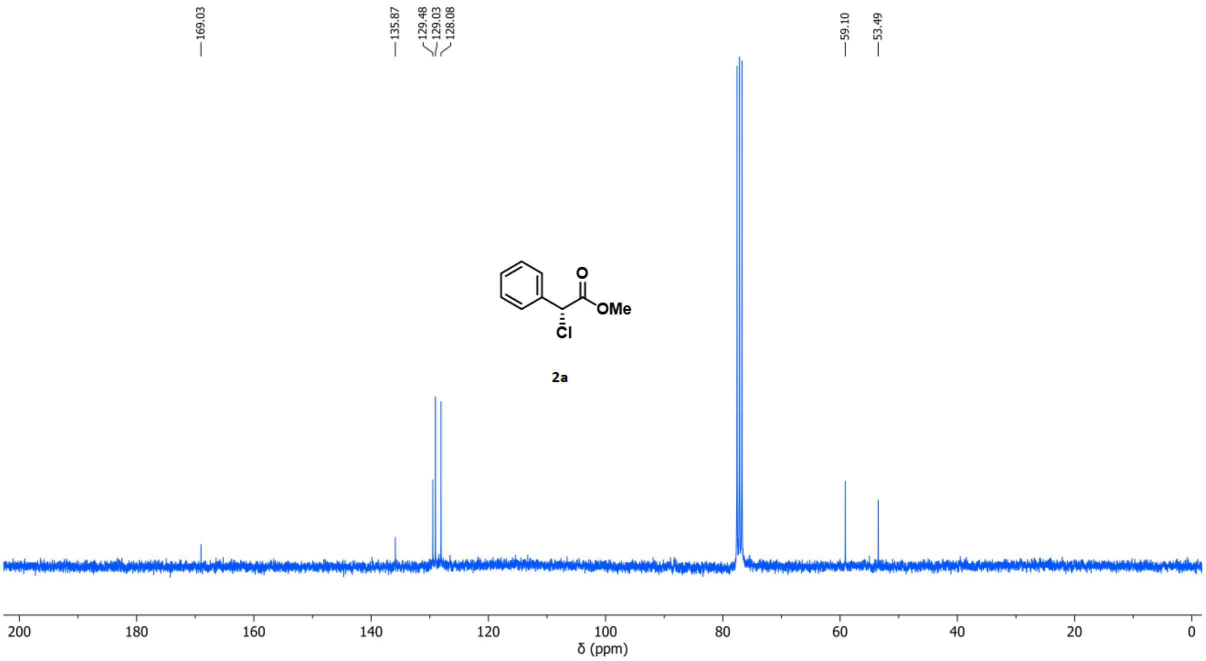

^{1}H-NMR of **2b** (300 MHz, $CDCl_3$, 298 K):

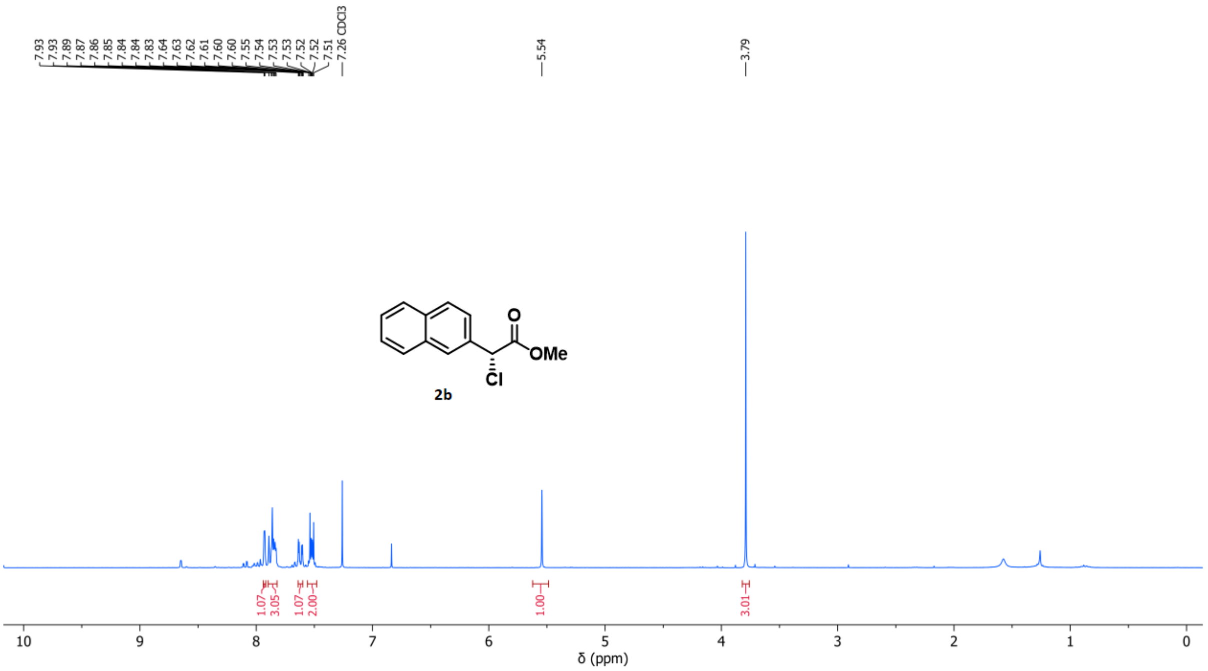

^{13}C-NMR of **2b** (75 MHz, $CDCl_3$, 298 K):

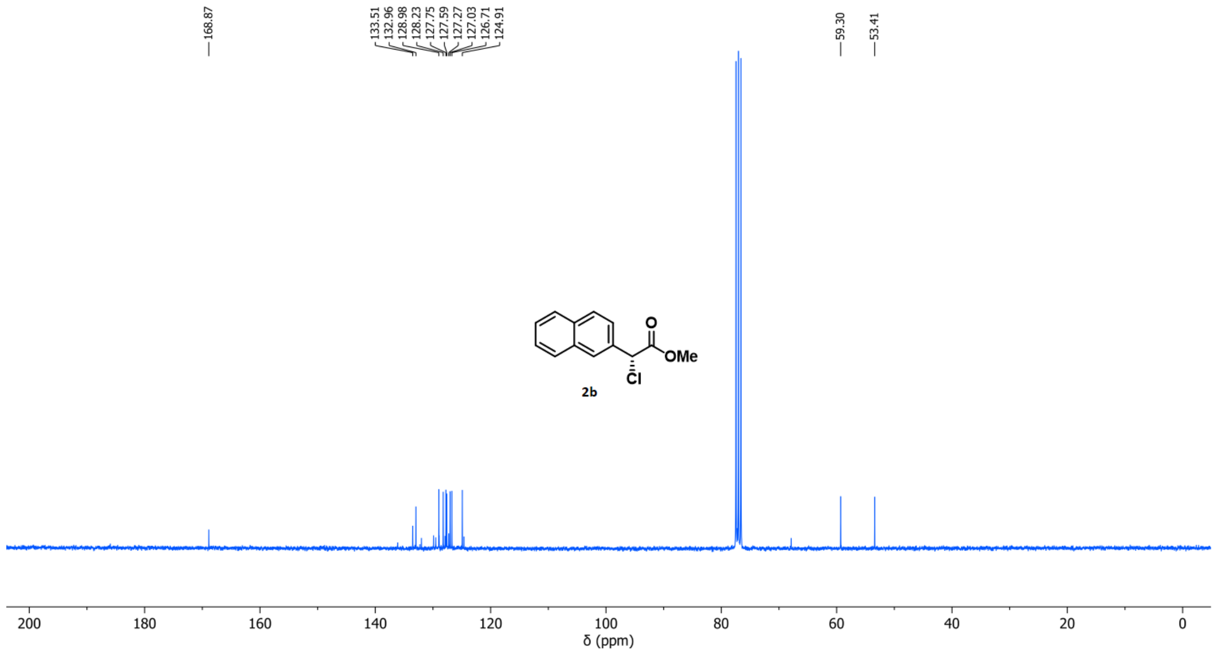

^{1}H-NMR of **2c** (300 MHz, $CDCl_3$, 298 K):

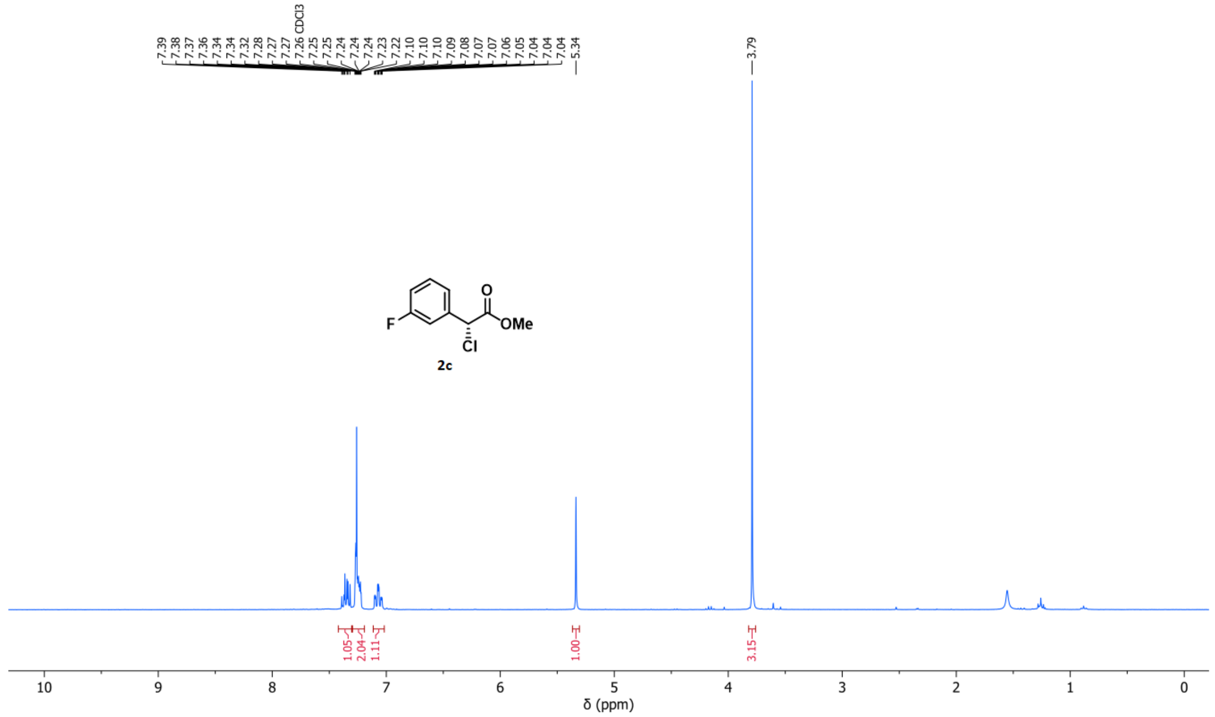

^{13}C-NMR of **2c** (75 MHz, $CDCl_3$, 298 K):

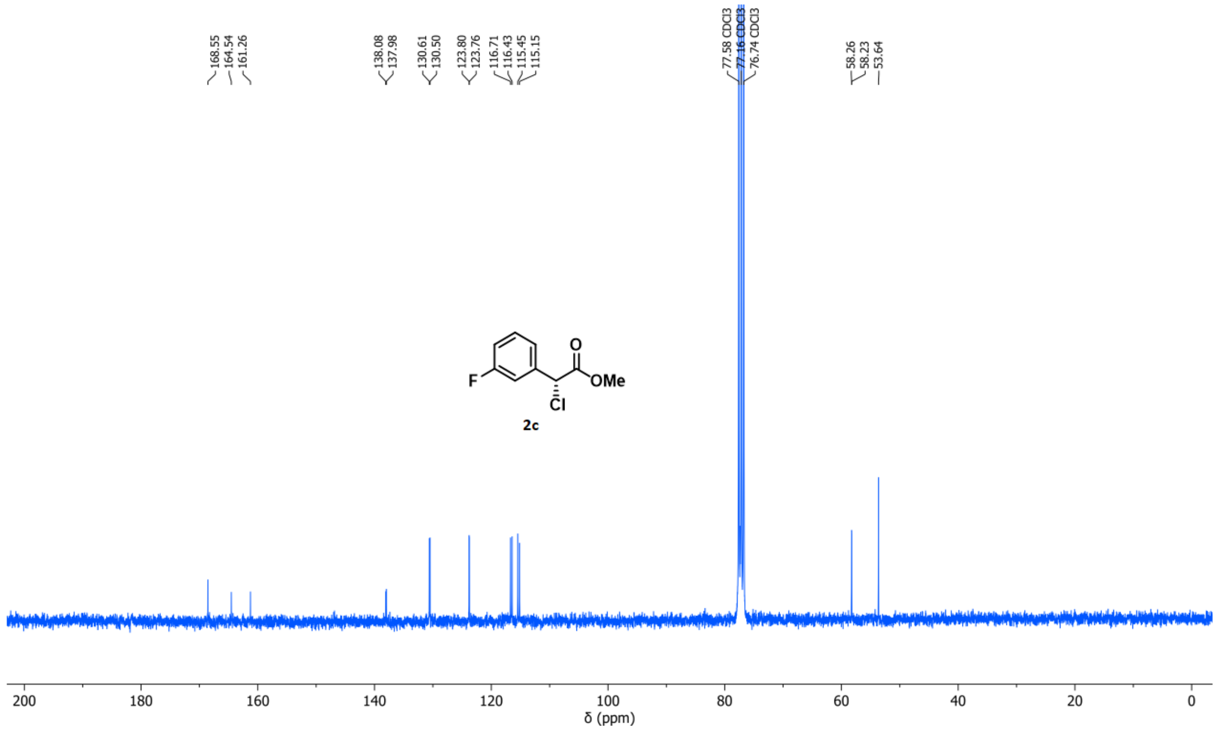

^{19}F-NMR of **2c** (282 MHz, $CDCl_3$, 298 K):

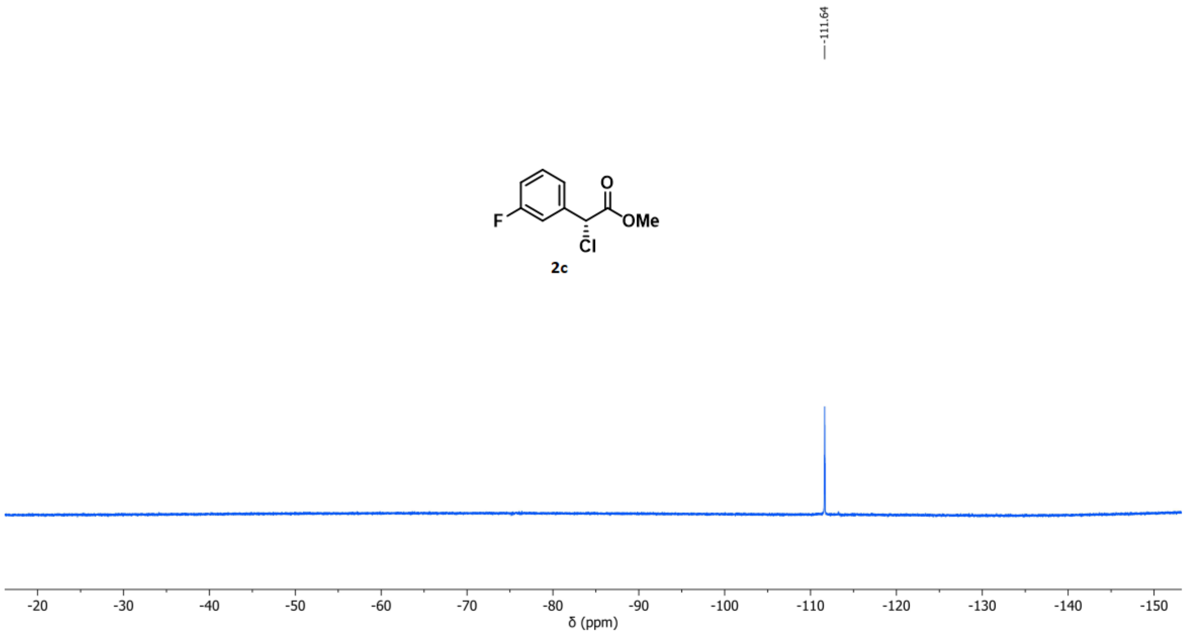

^{1}H-NMR of **2d** (300 MHz, $CDCl_3$, 298 K):

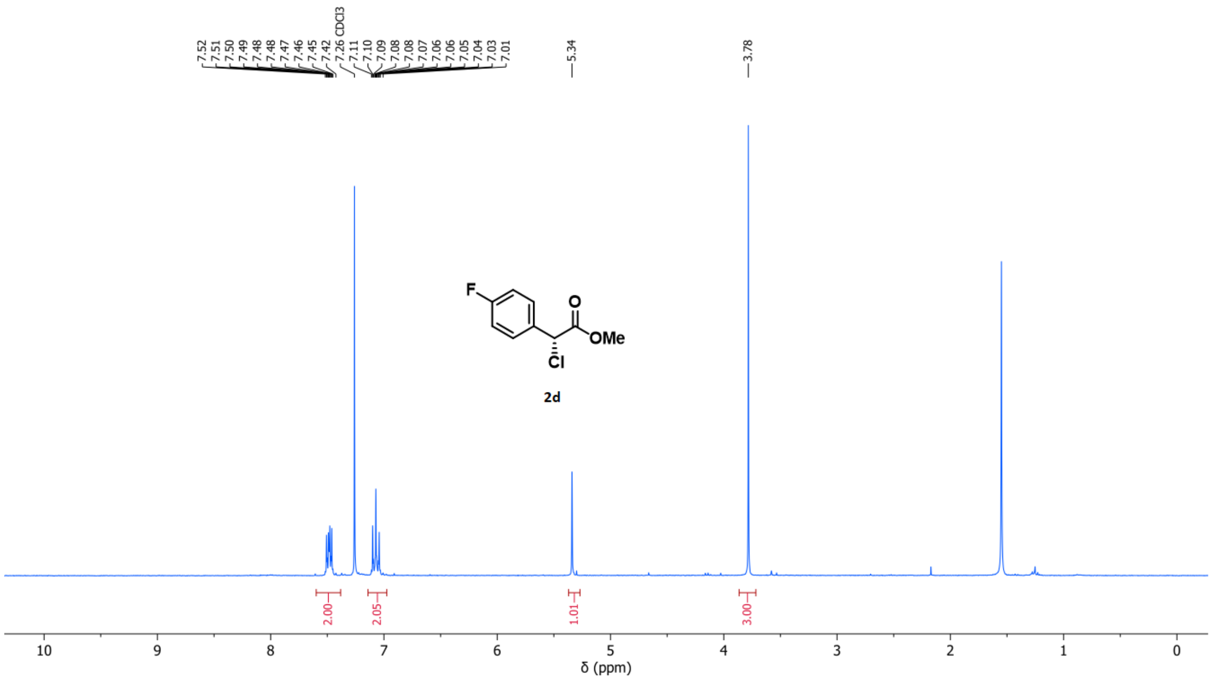

^{13}C-NMR of **2d** (75 MHz, $CDCl_3$, 298 K):

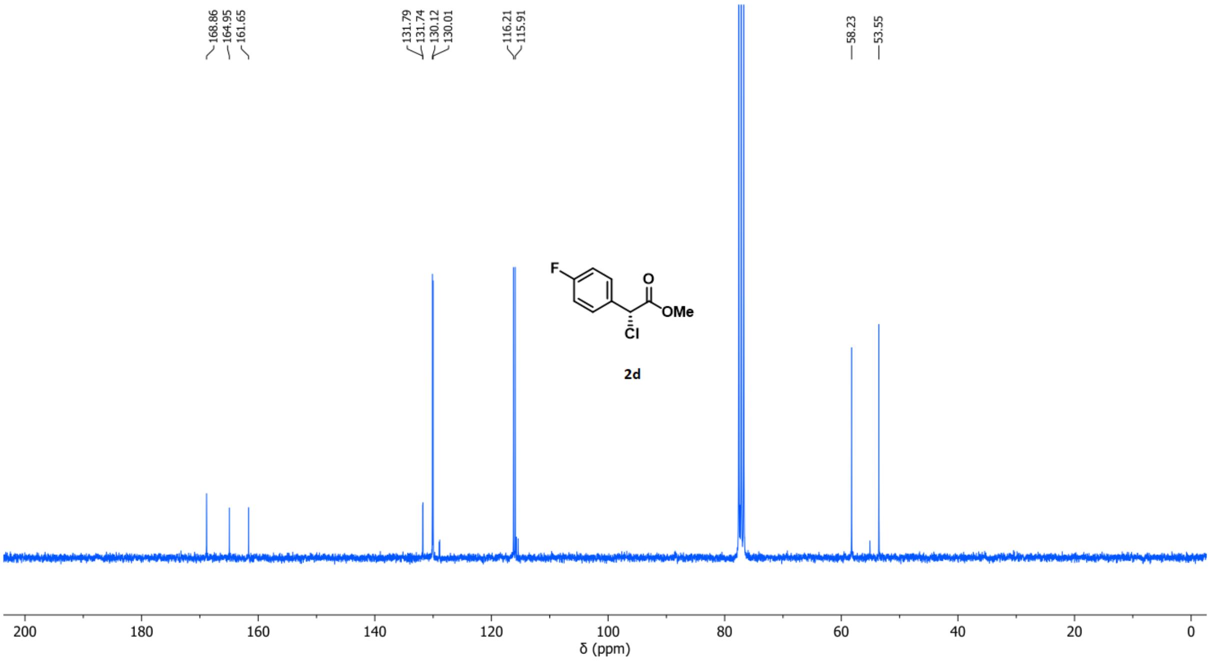

^{19}F-NMR of **2d** (282 MHz, $CDCl_3$, 298 K):

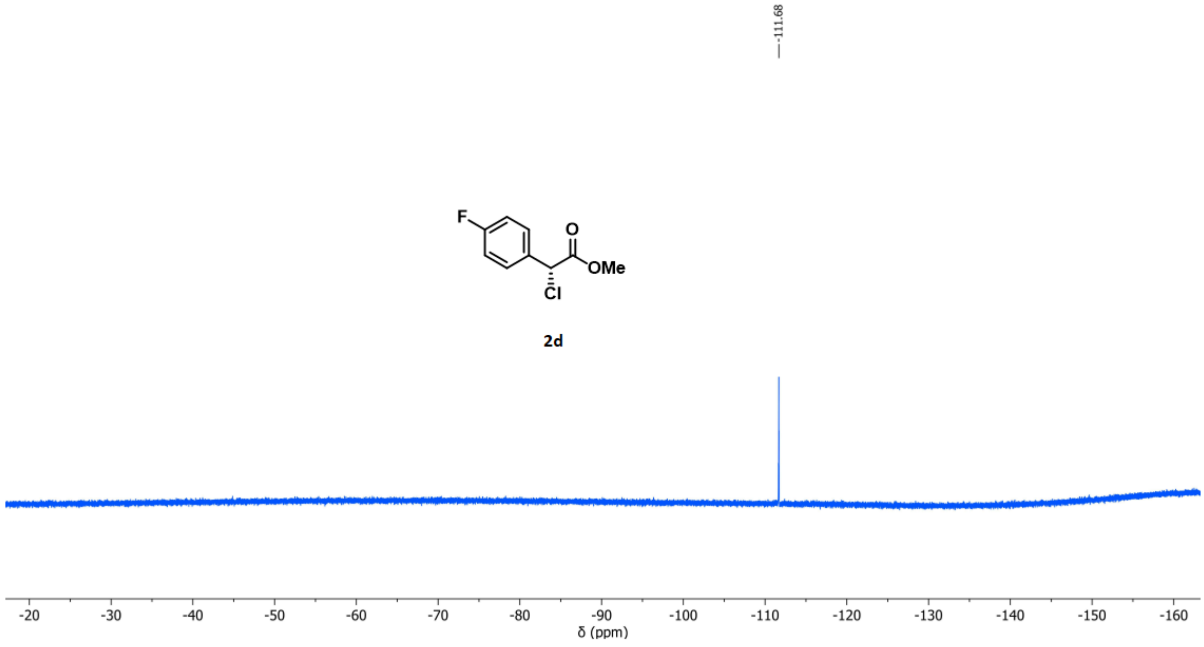

^{1}H-NMR of **2e** (300 MHz, $CDCl_3$, 298 K):

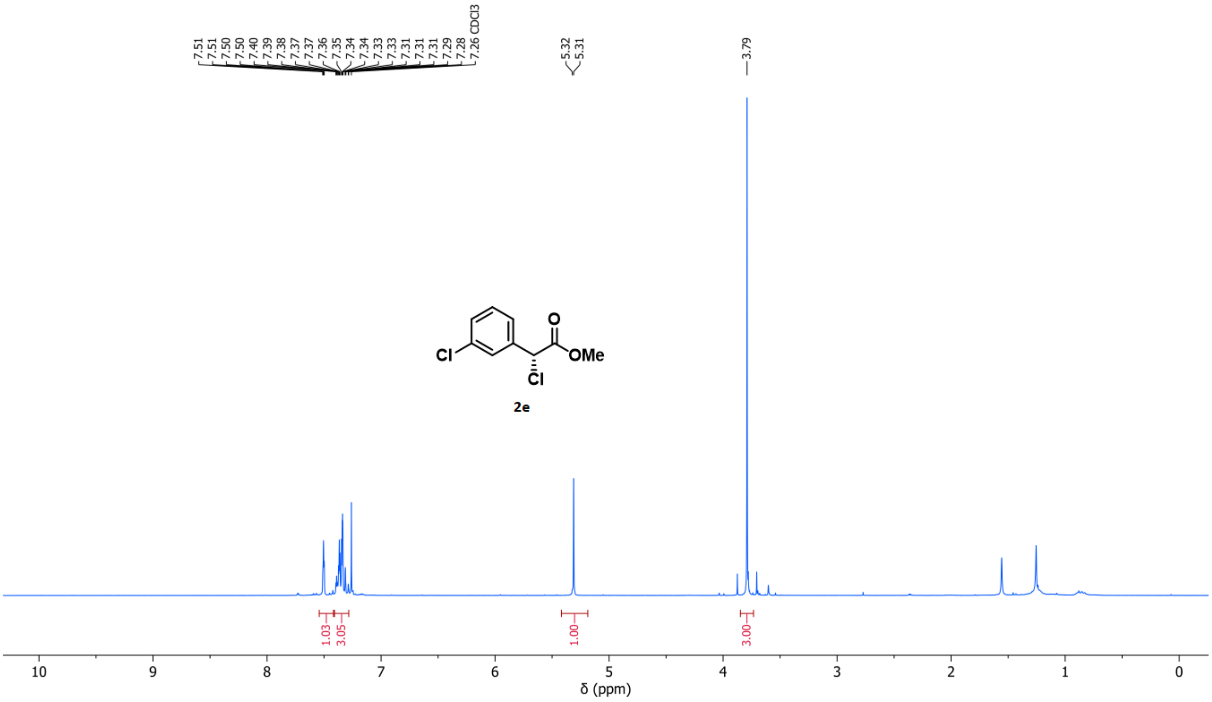

^{13}C-NMR of **2e** (75 MHz, $CDCl_3$, 298 K):

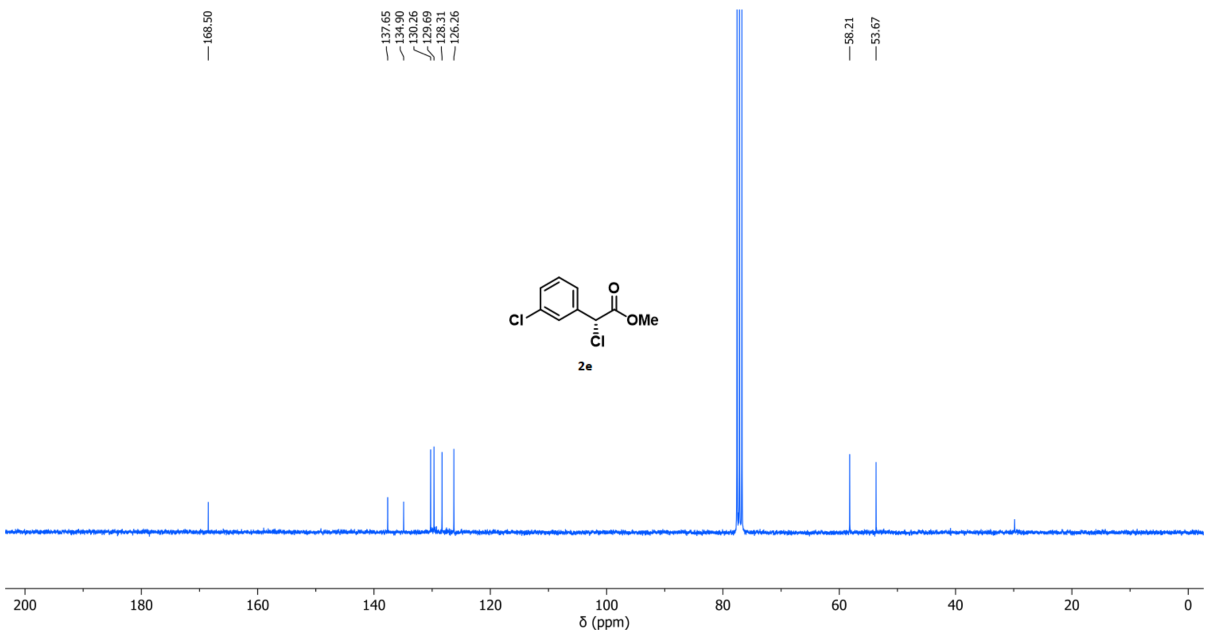

^{1}H-NMR of **2f** (300 MHz, $CDCl_3$, 298 K):

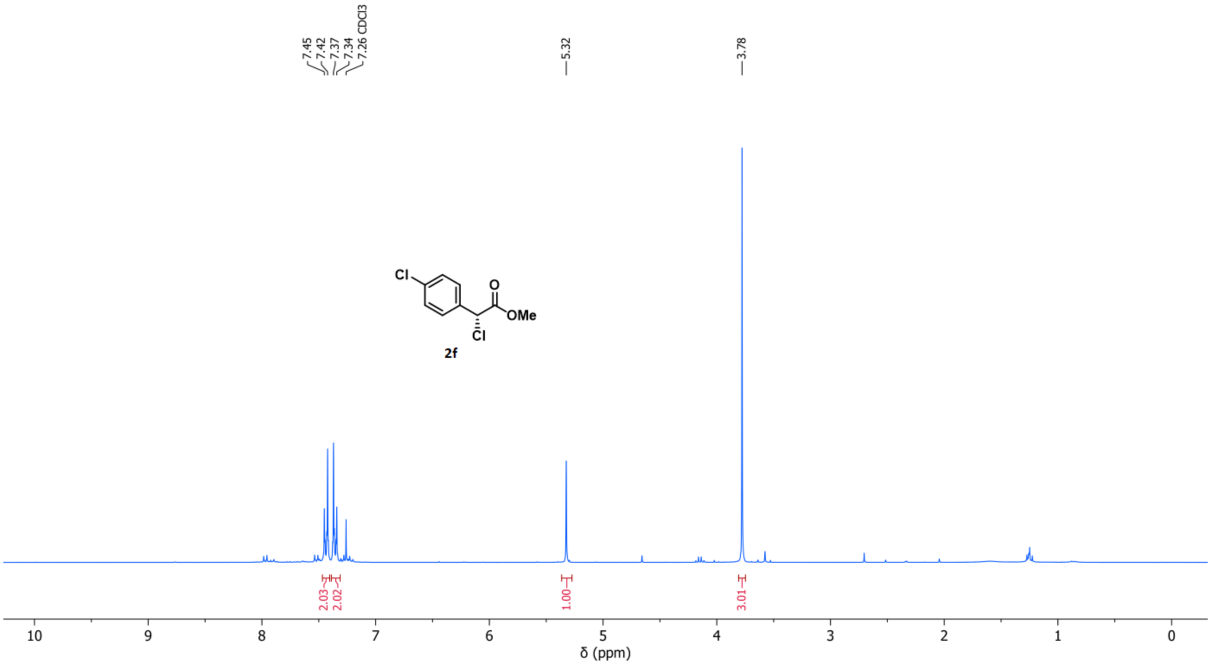

^{13}C-NMR of **2f** (75 MHz, $CDCl_3$, 298 K):

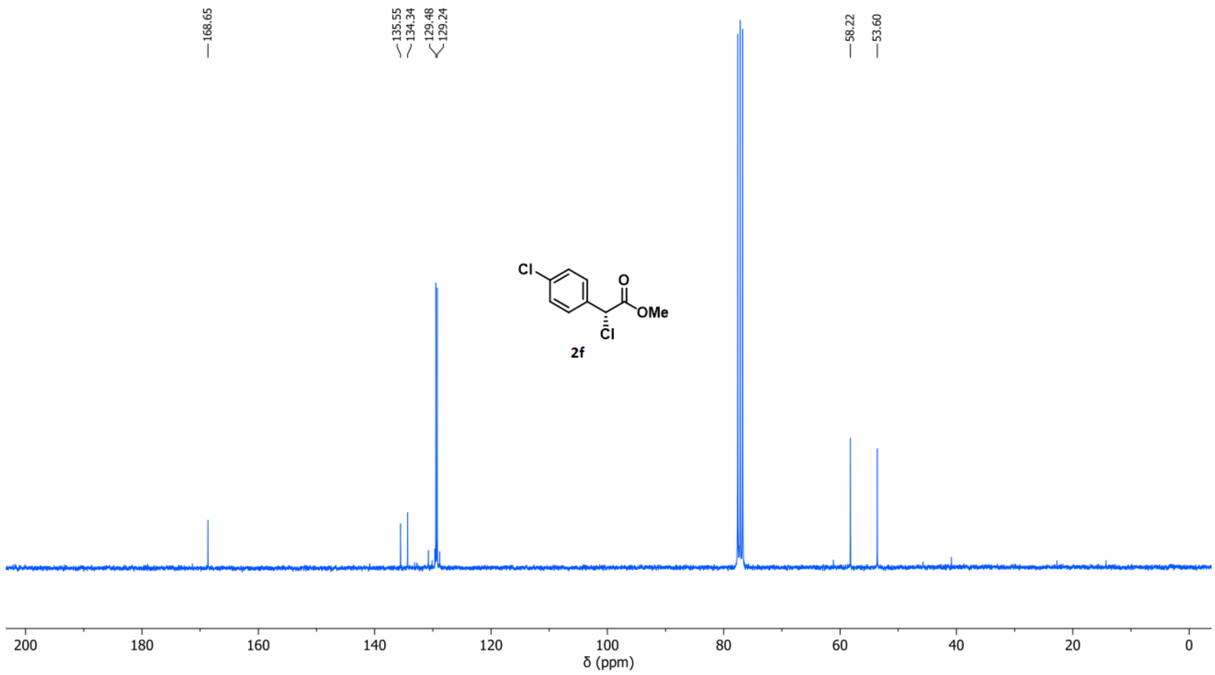

^{1}H-NMR of **2g** (300 MHz, $CDCl_3$, 298 K):

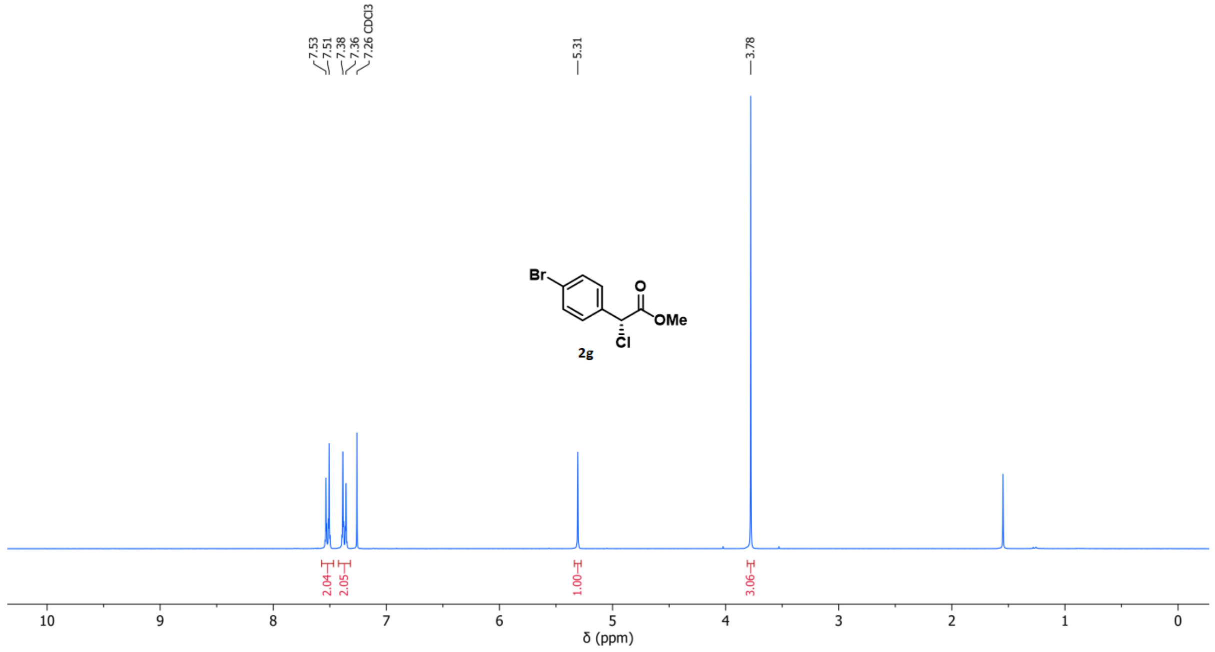

^{13}C-NMR of **2g** (75 MHz, $CDCl_3$, 298 K):

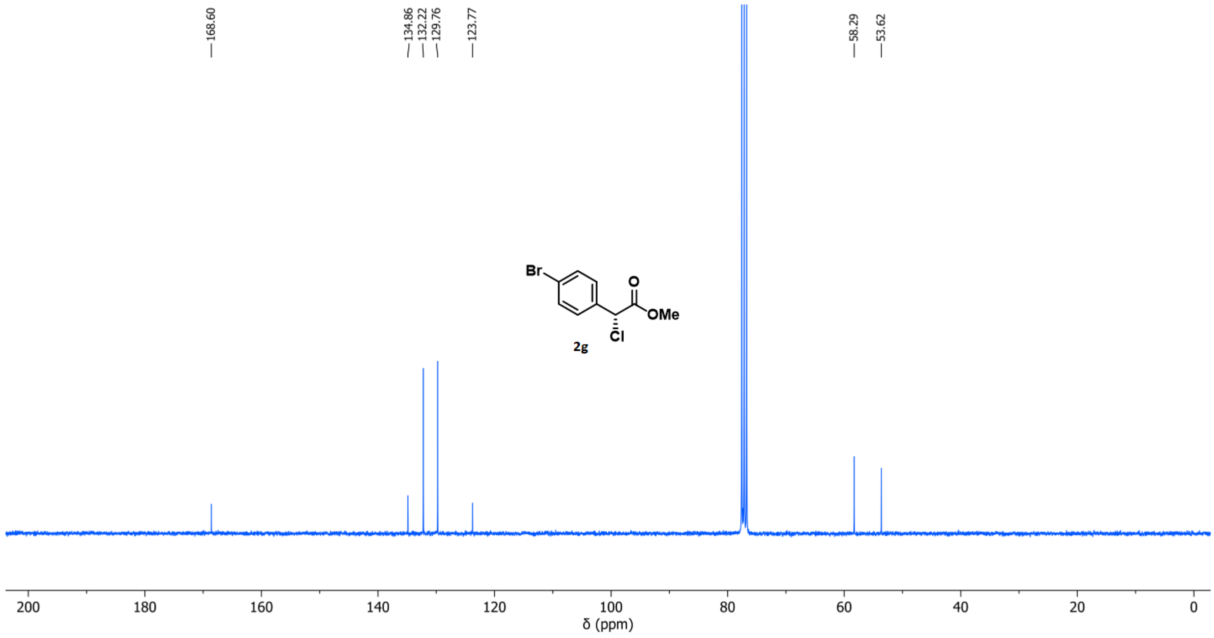

^{1}H-NMR of **2h** (300 MHz, $CDCl_3$, 298 K):

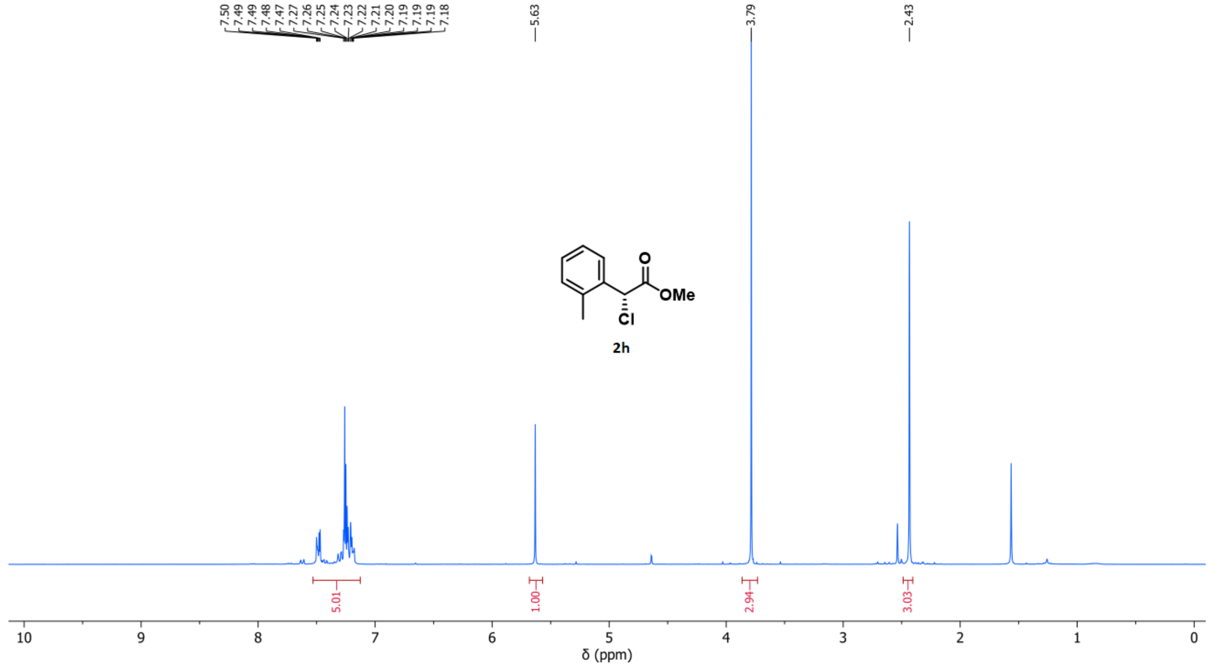

^{13}C-NMR of **2h** (75 MHz, $CDCl_3$, 298 K):

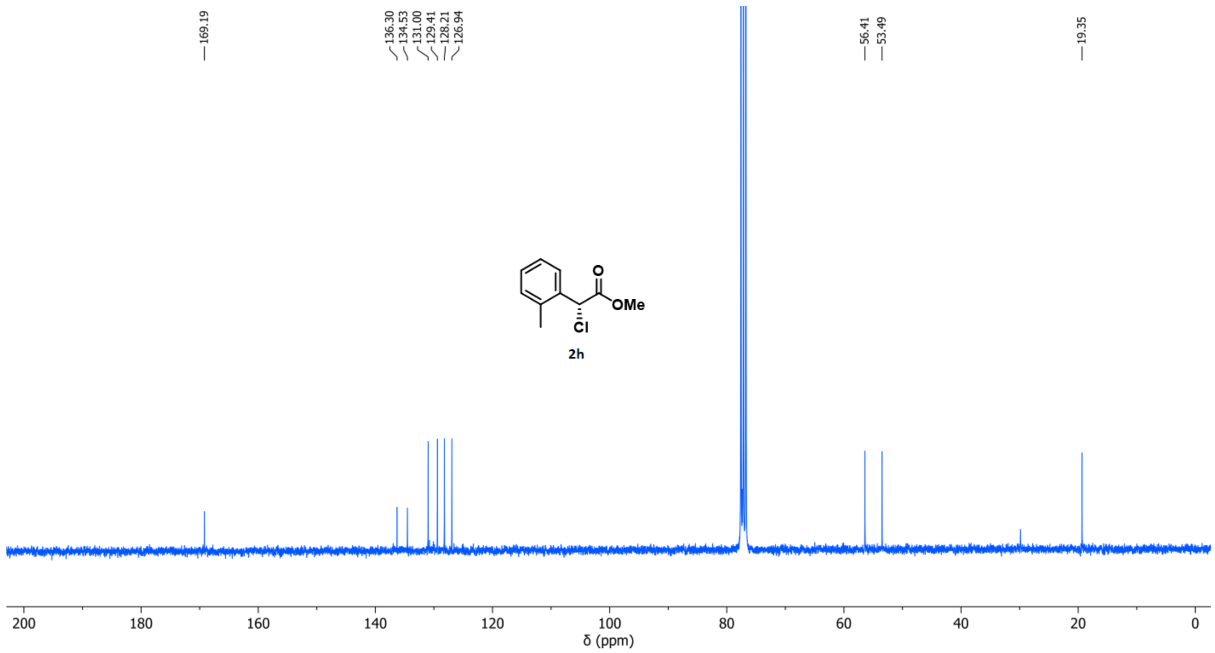

^{1}H-NMR of **2i** (300 MHz, $CDCl_3$, 298 K):

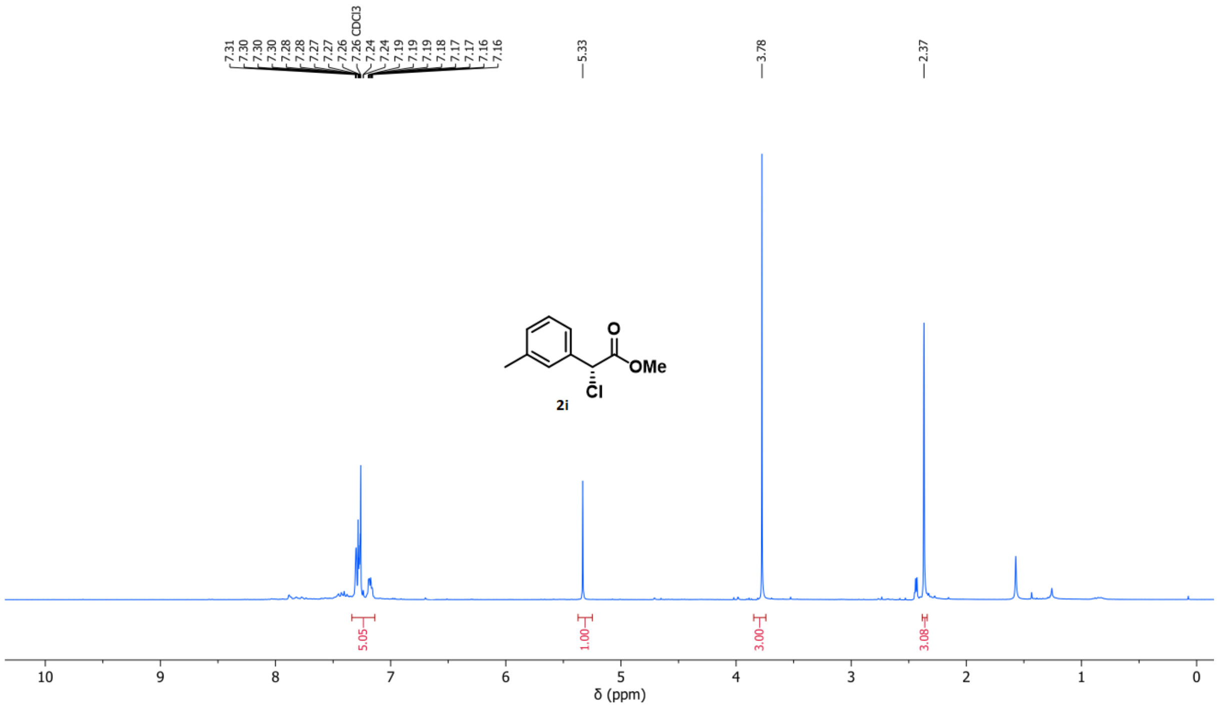

^{13}C-NMR of **2i** (75 MHz, $CDCl_3$, 298 K):

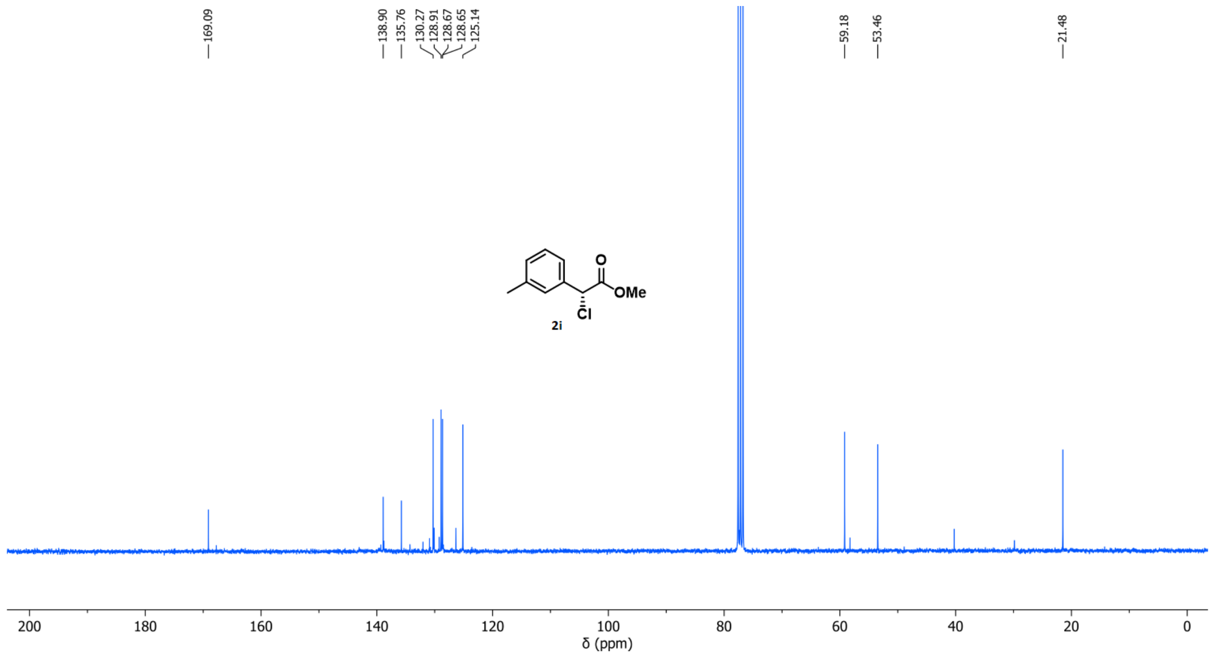

^{1}H-NMR of **2j** (300 MHz, $CDCl_3$, 298 K):

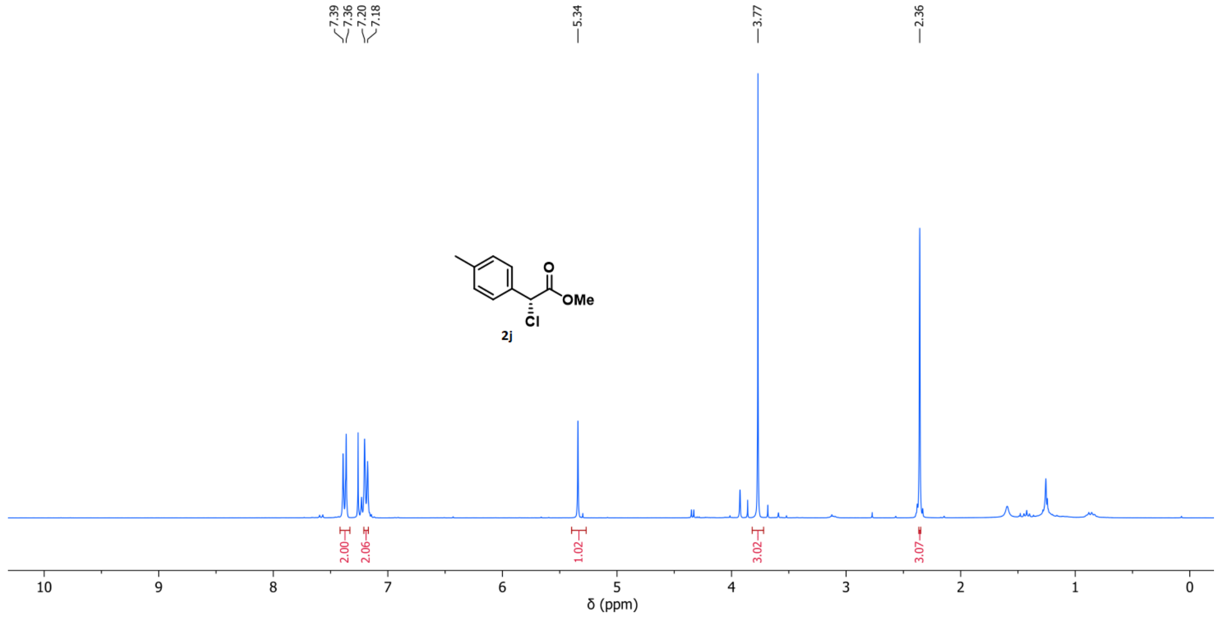

^{13}C-NMR of **2j** (75 MHz, $CDCl_3$, 298 K):

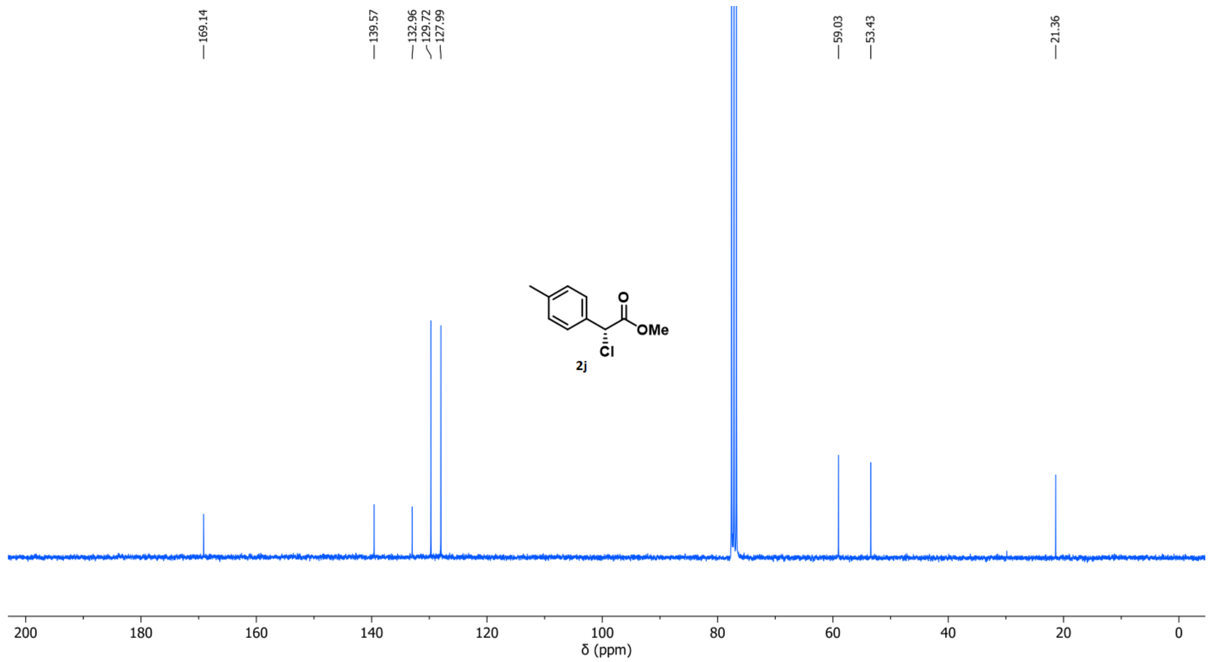

^{1}H-NMR of **2k** (300 MHz, $CDCl_3$, 298 K):

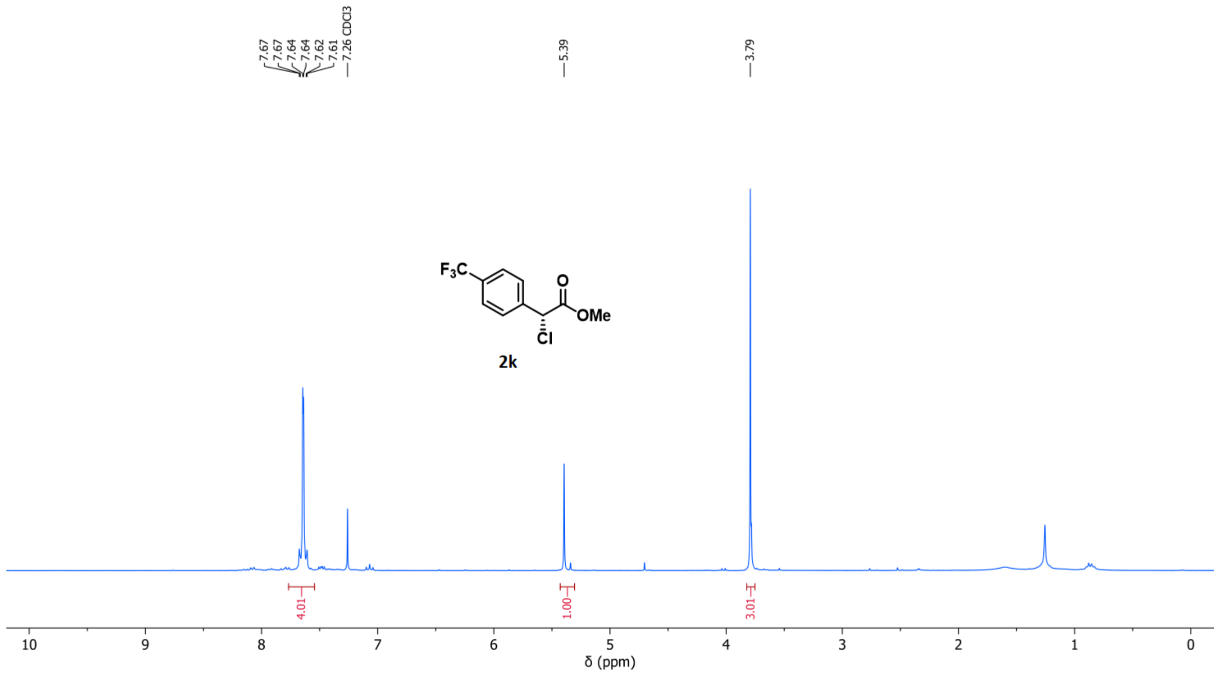

^{13}C-NMR of **2k** (126 MHz, $CDCl_3$, 298 K):

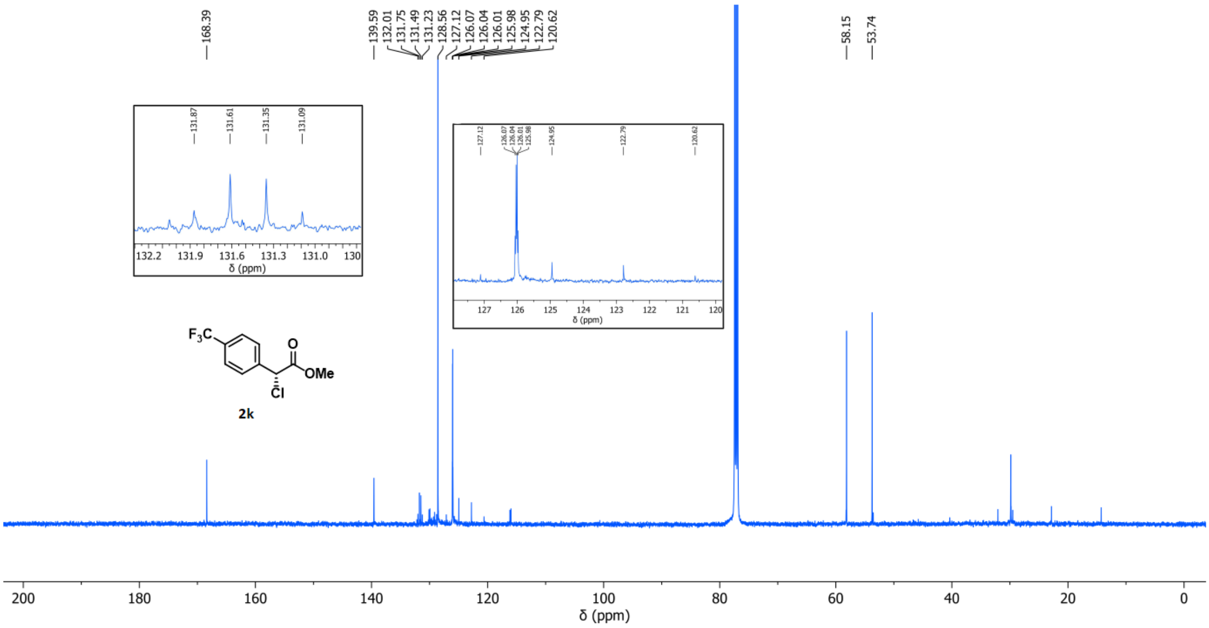

^{19}F-NMR of **2k** (282 MHz, $CDCl_3$, 298 K):

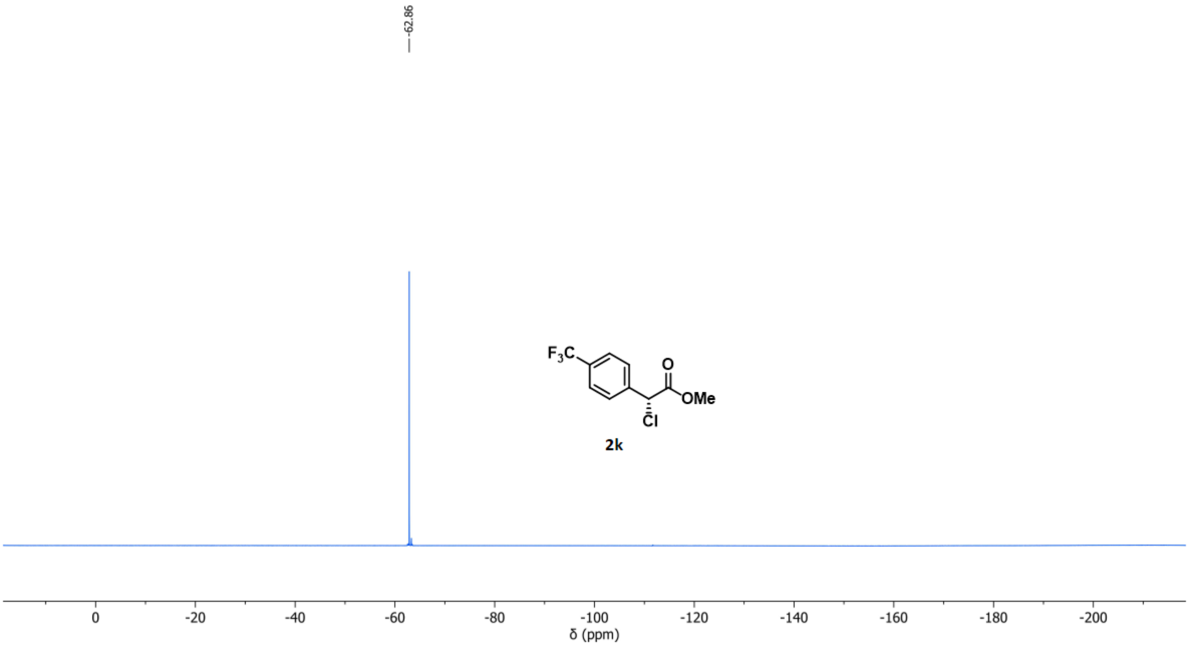

^{1}H-NMR of **2l** (300 MHz, $CDCl_3$, 298 K):

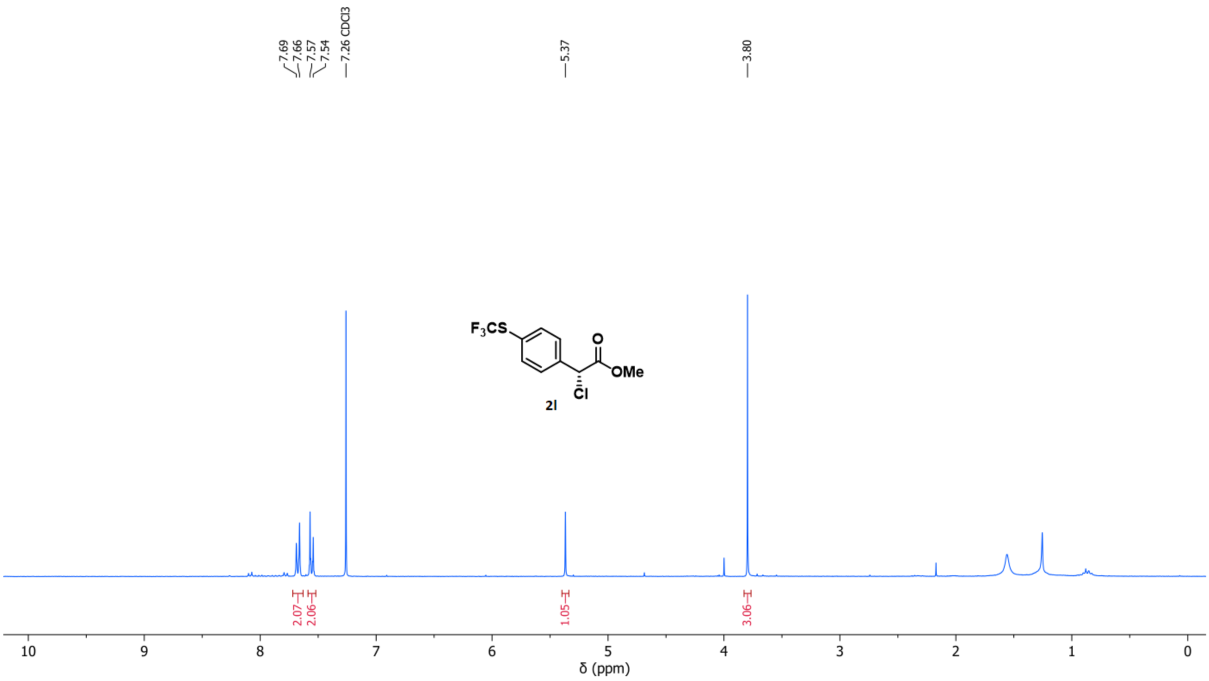

^{13}C-NMR of **2l** (126 MHz, $CDCl_3$, 298 K):

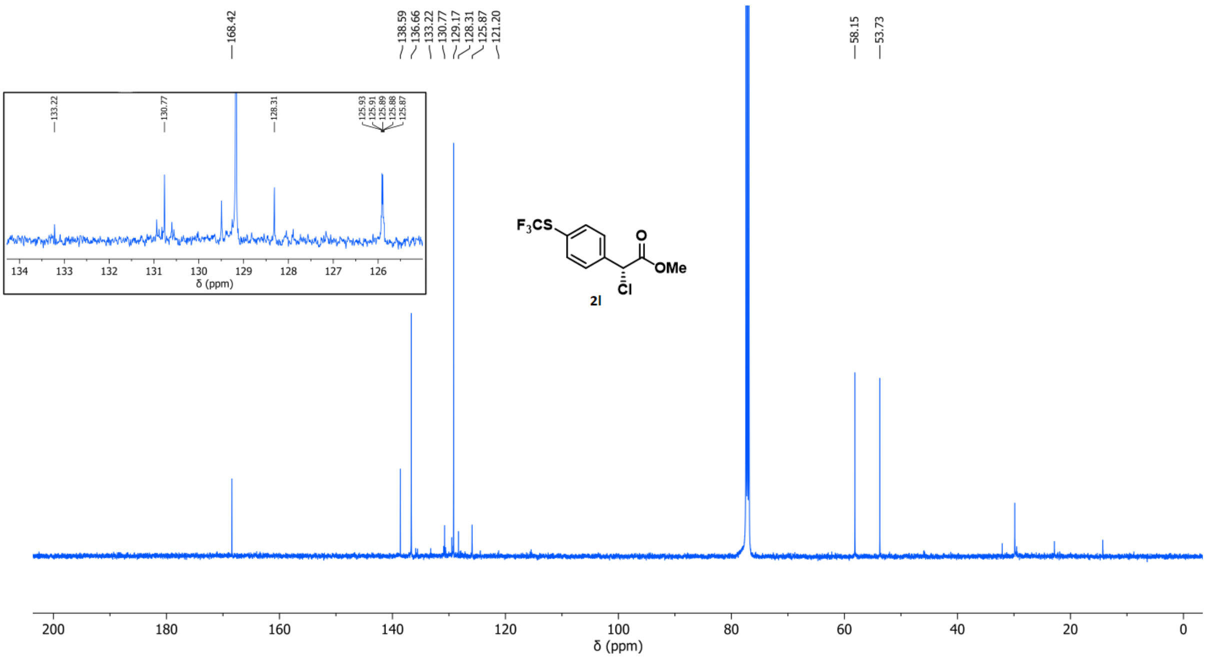

^{19}F-NMR of **2l** (282 MHz, $CDCl_3$, 298 K):

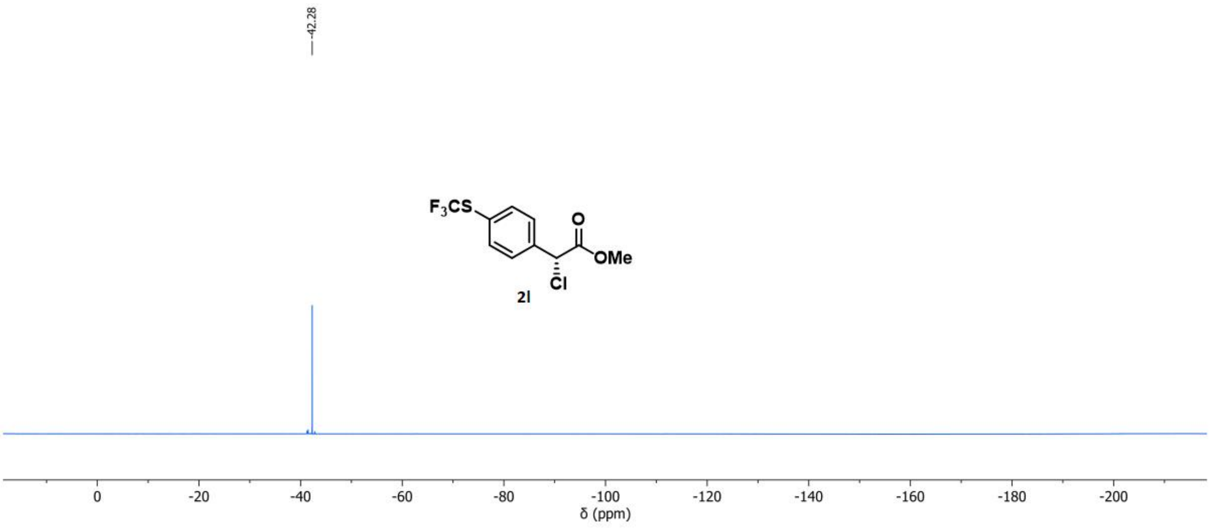

^{1}H-NMR of **2m** (300 MHz, $CDCl_3$, 298 K):

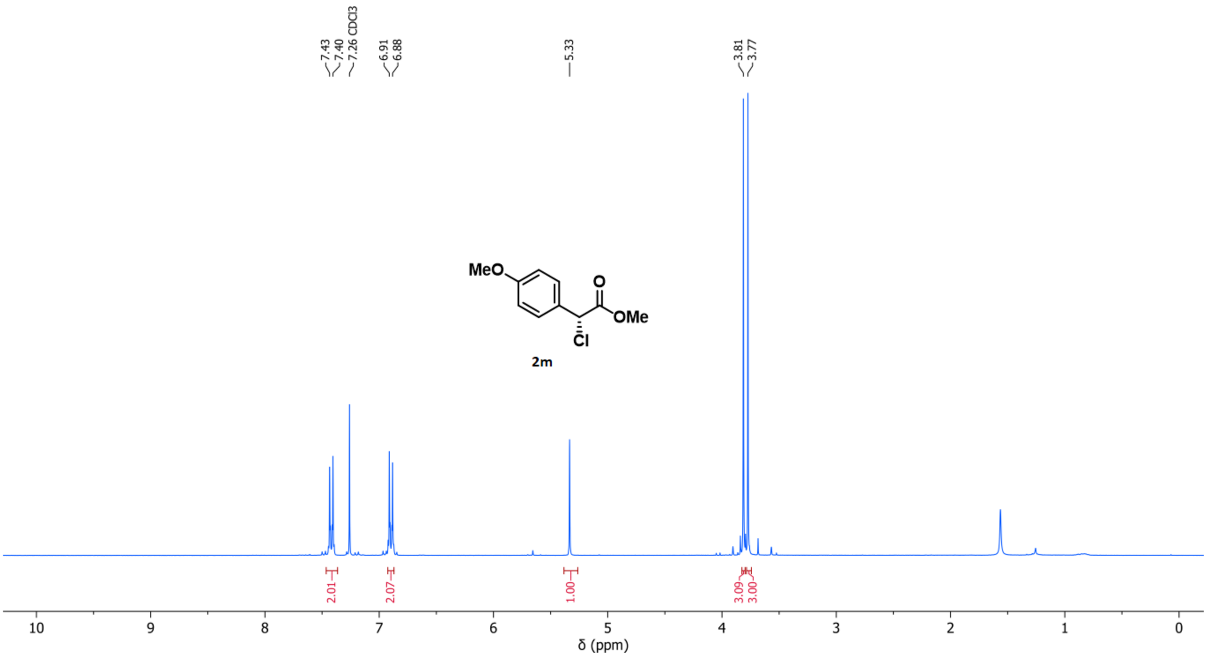

^{13}C-NMR of **2m** (75 MHz, $CDCl_3$, 298 K):

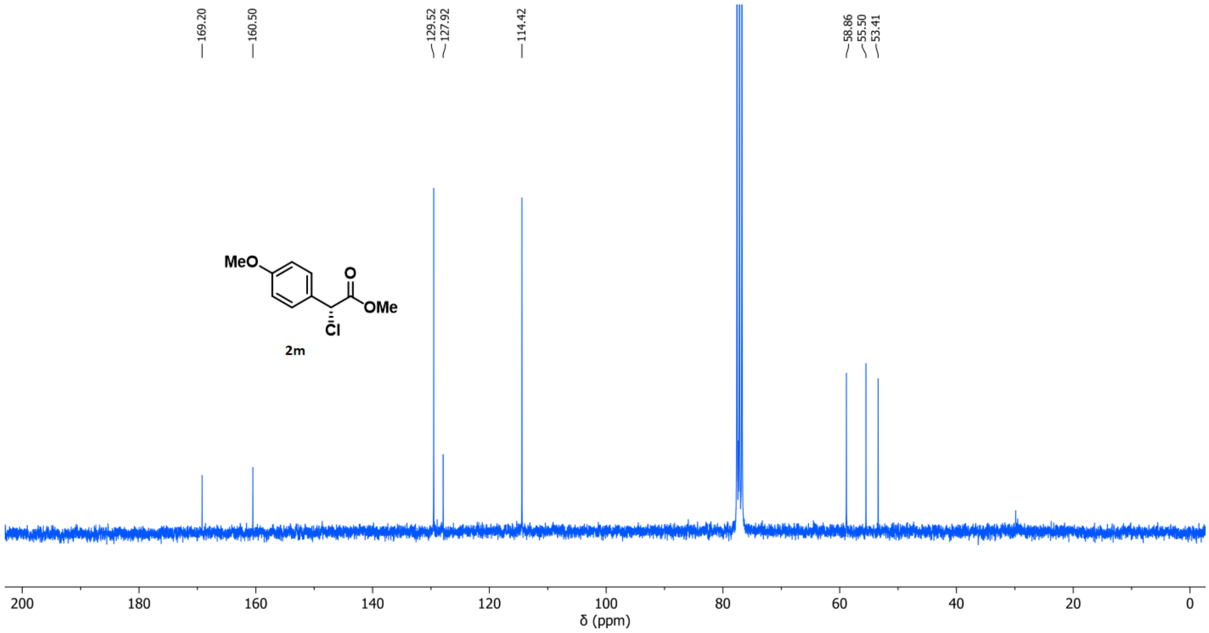

^{1}H-NMR of **2n** (300 MHz, $CDCl_3$, 298 K):

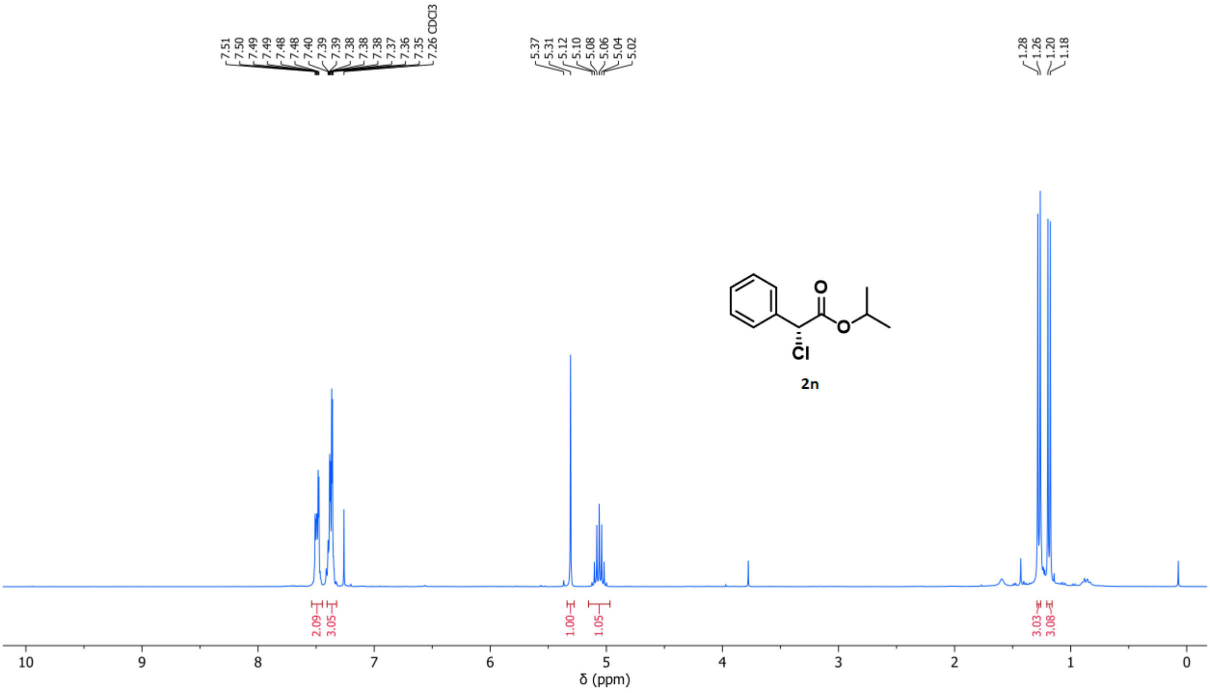

^{13}C-NMR of **2n** (75 MHz, $CDCl_3$, 298 K):

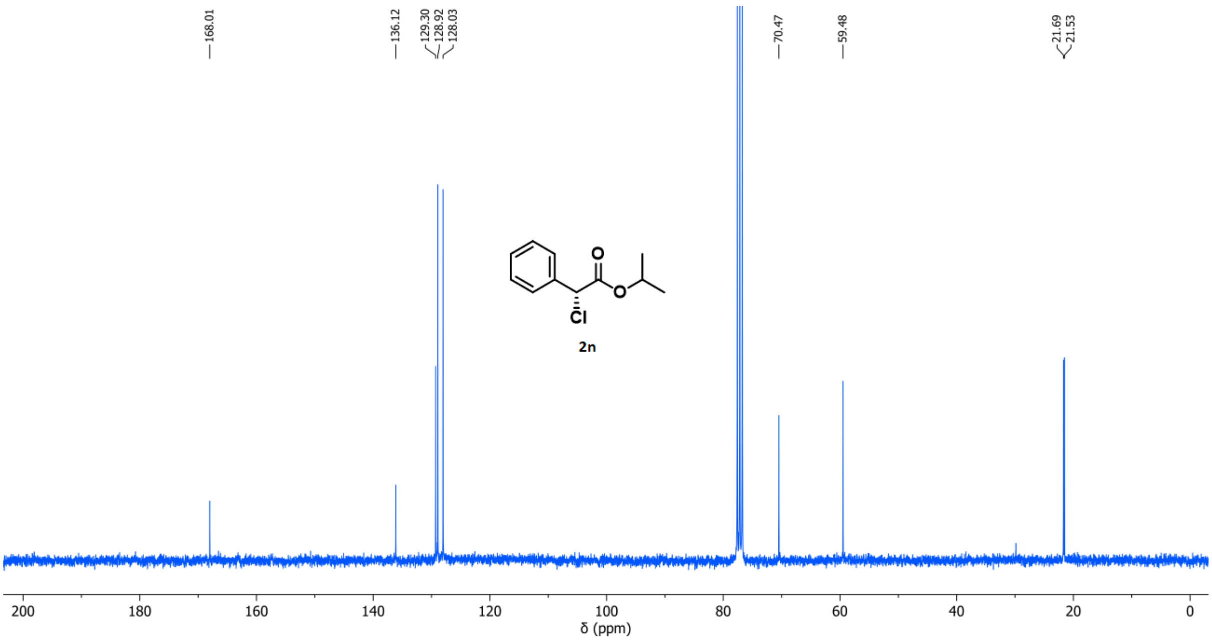

^{1}H-NMR of **2o** (300 MHz, $CDCl_3$, 298 K):

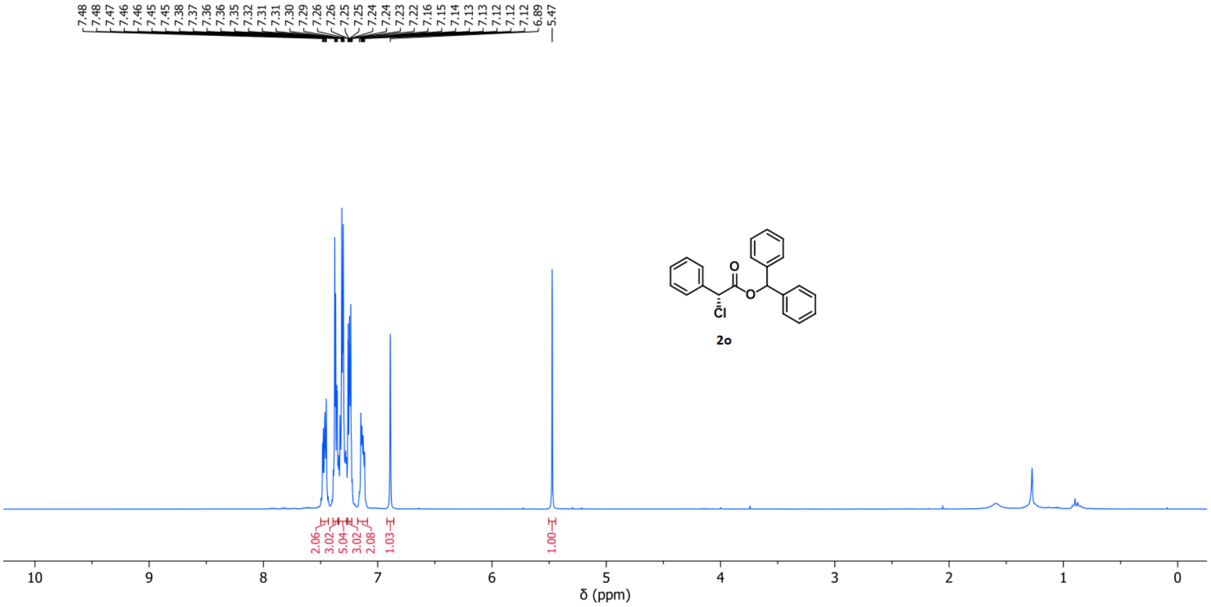

^{13}C-NMR of **2o** (75 MHz, $CDCl_3$, 298 K):

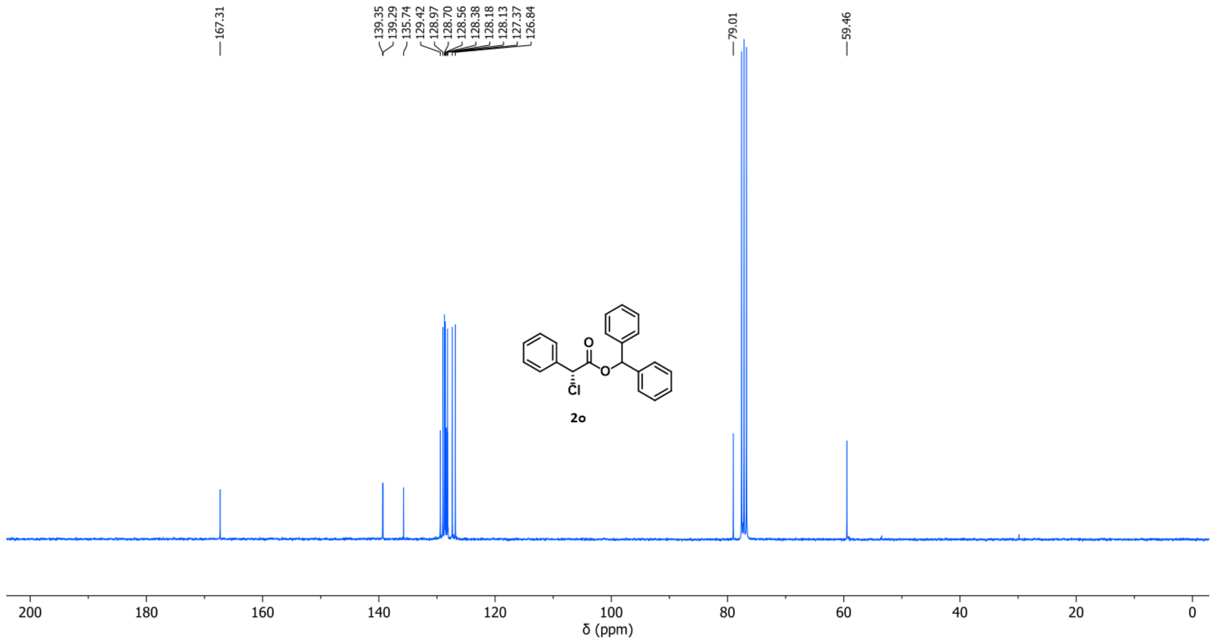

^{1}H-NMR of **4** (300 MHz, $CDCl_3$, 298 K):

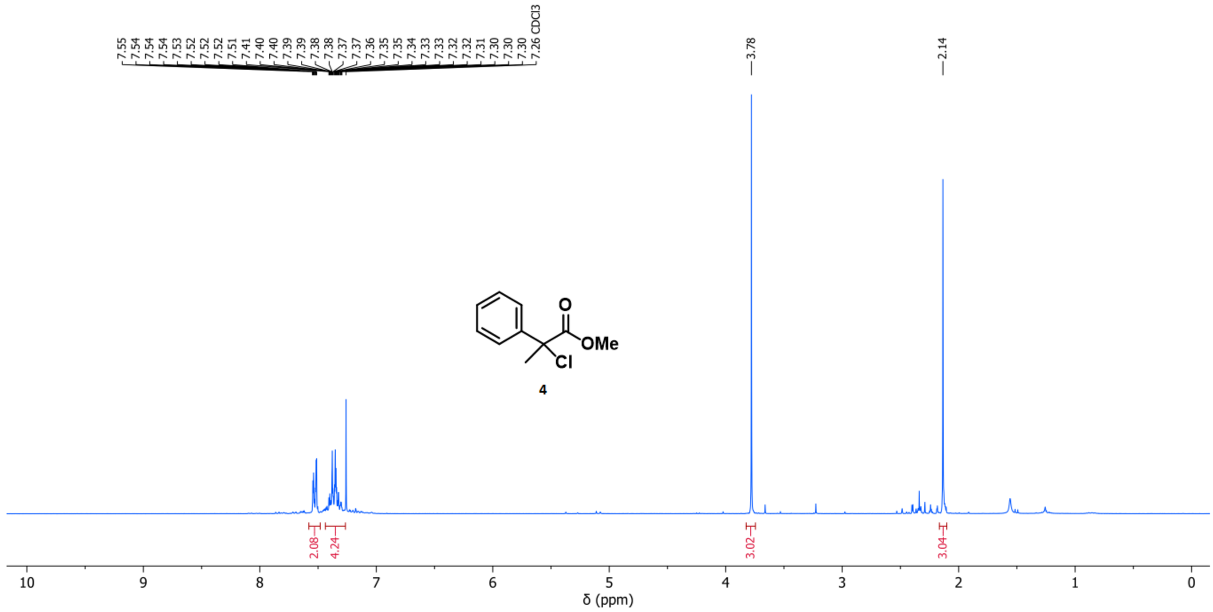

^{13}C-NMR of **4** (75 MHz, $CDCl_3$, 298 K):

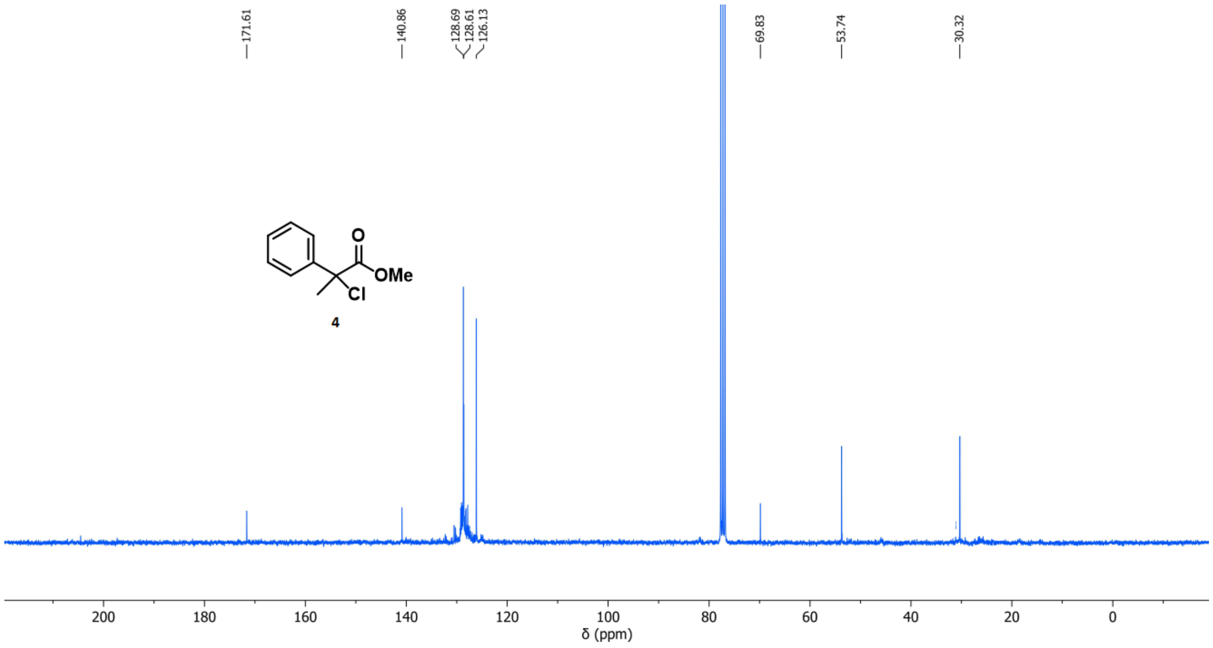

^{1}H-NMR of **6** (300 MHz, $CDCl_3$, 298 K):

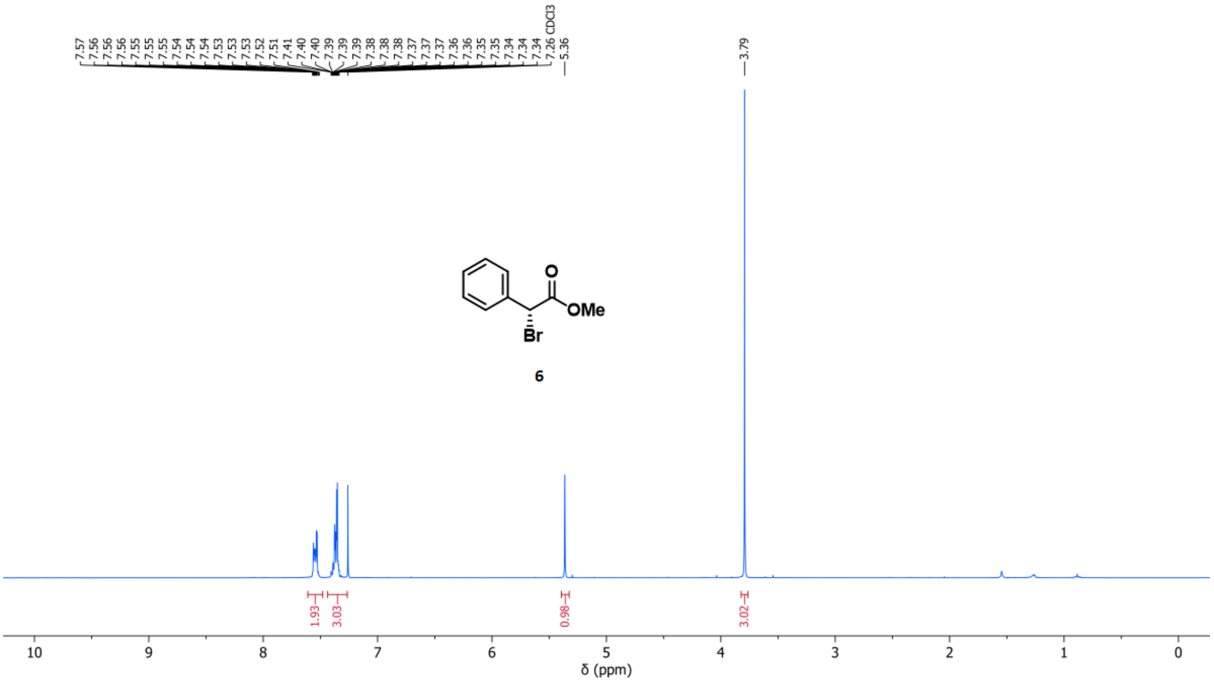

^{13}C-NMR of **6** (75 MHz, $CDCl_3$, 298 K):

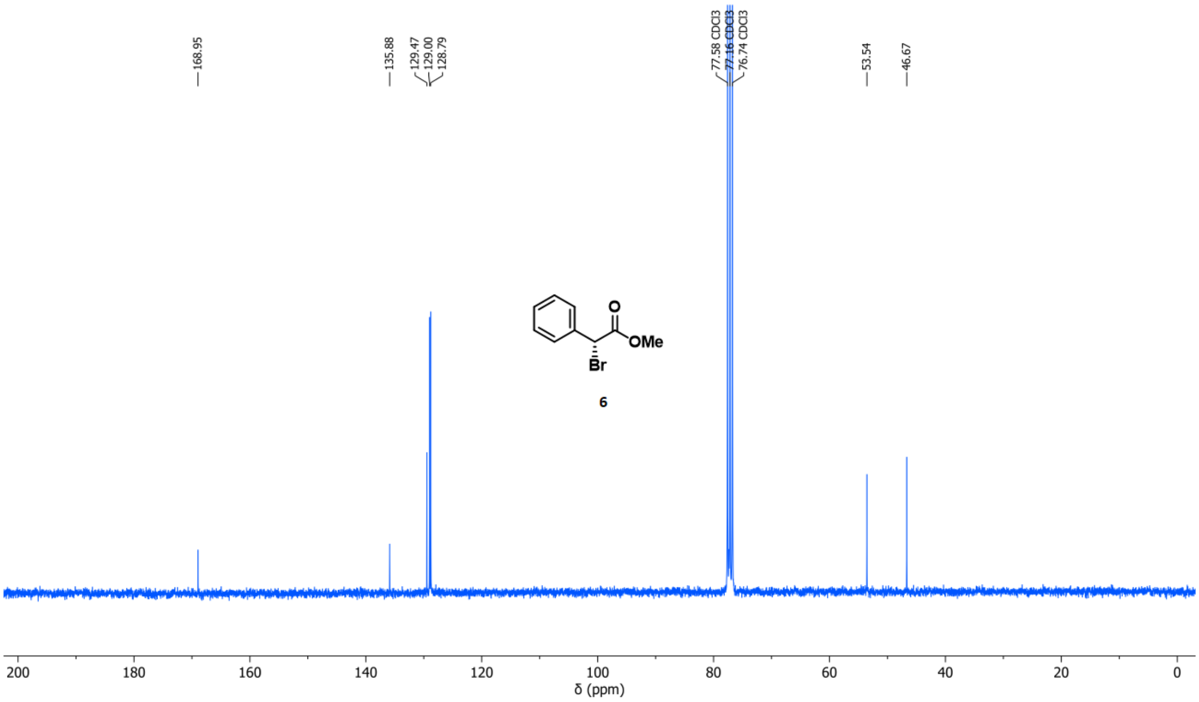

^{1}H-NMR of **7** (300 MHz, $CDCl_3$, 298 K):

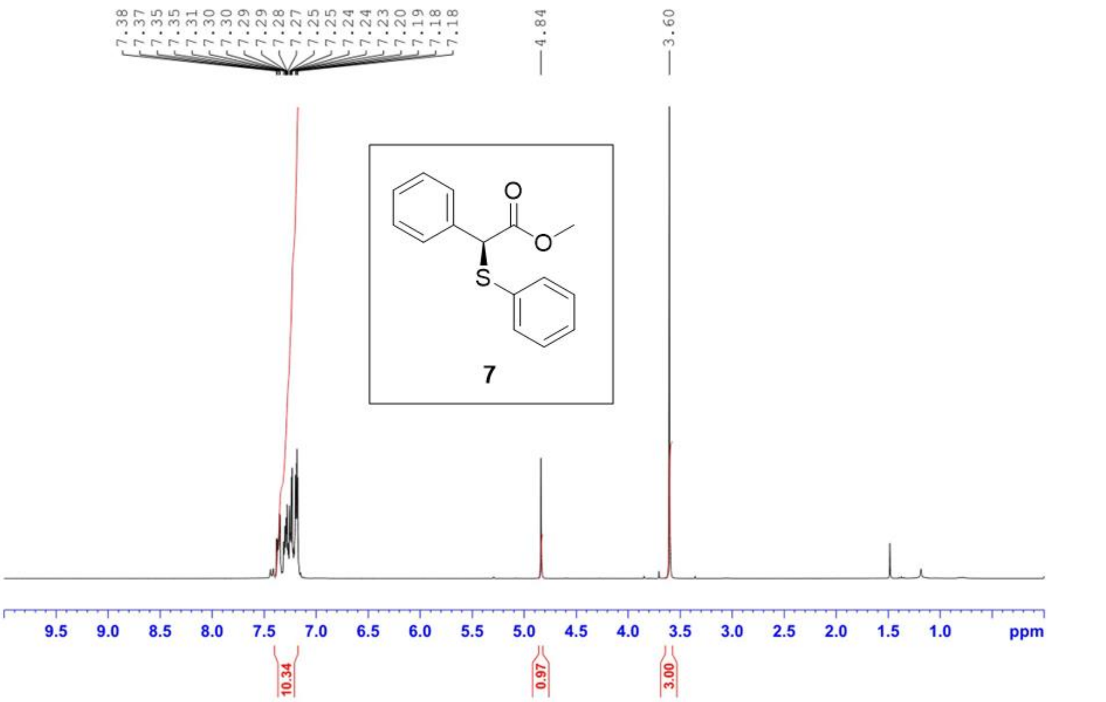

^{13}C-NMR of **7** (75 MHz, $CDCl_3$, 298 K):

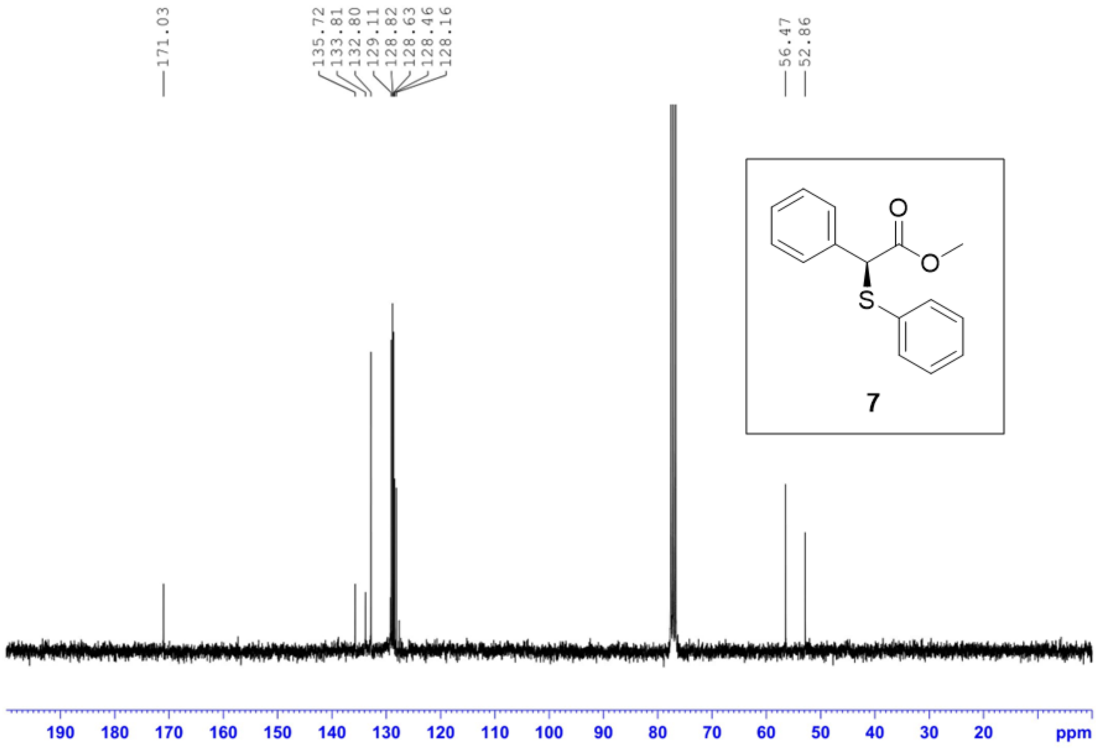

^{1}H-NMR of **8** (300 MHz, $CDCl_3$, 298 K):

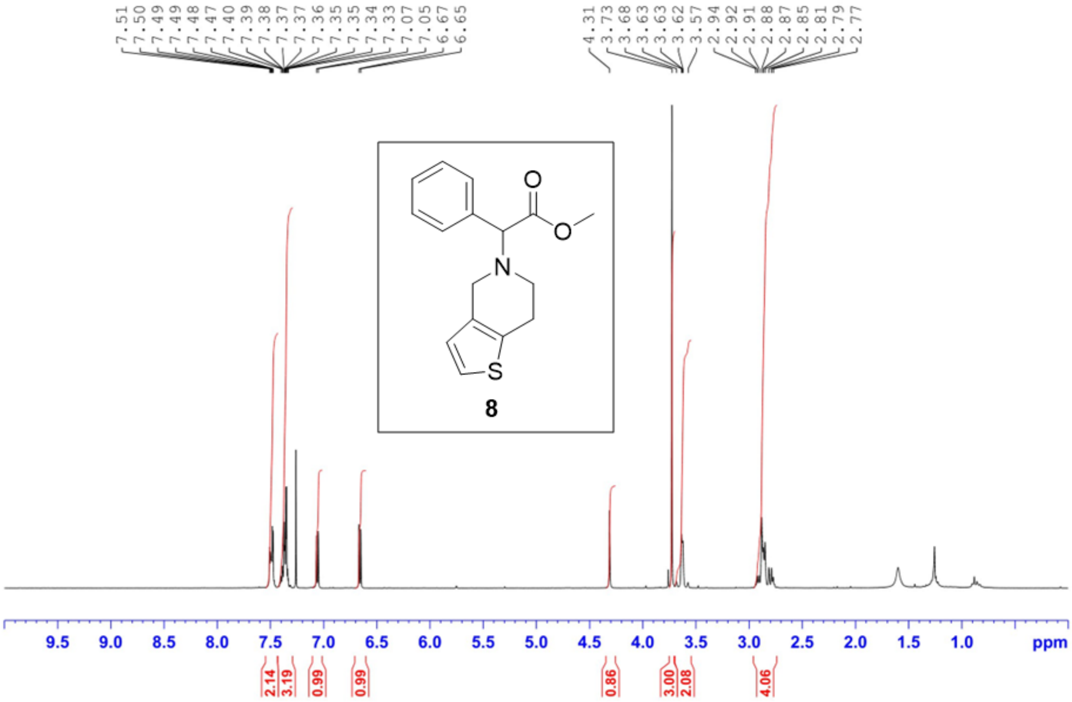

^{13}C-NMR of **8** (75 MHz, $CDCl_3$, 298 K):

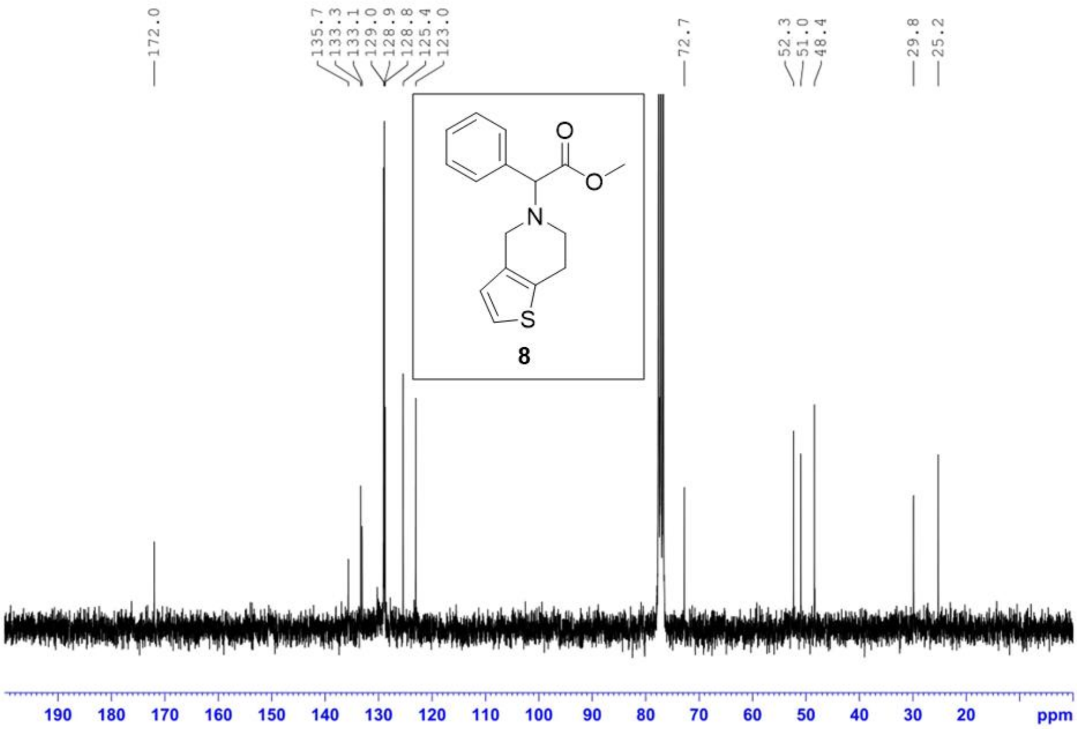

^{1}H-NMR of **9** (300 MHz, $CDCl_3$, 298 K):

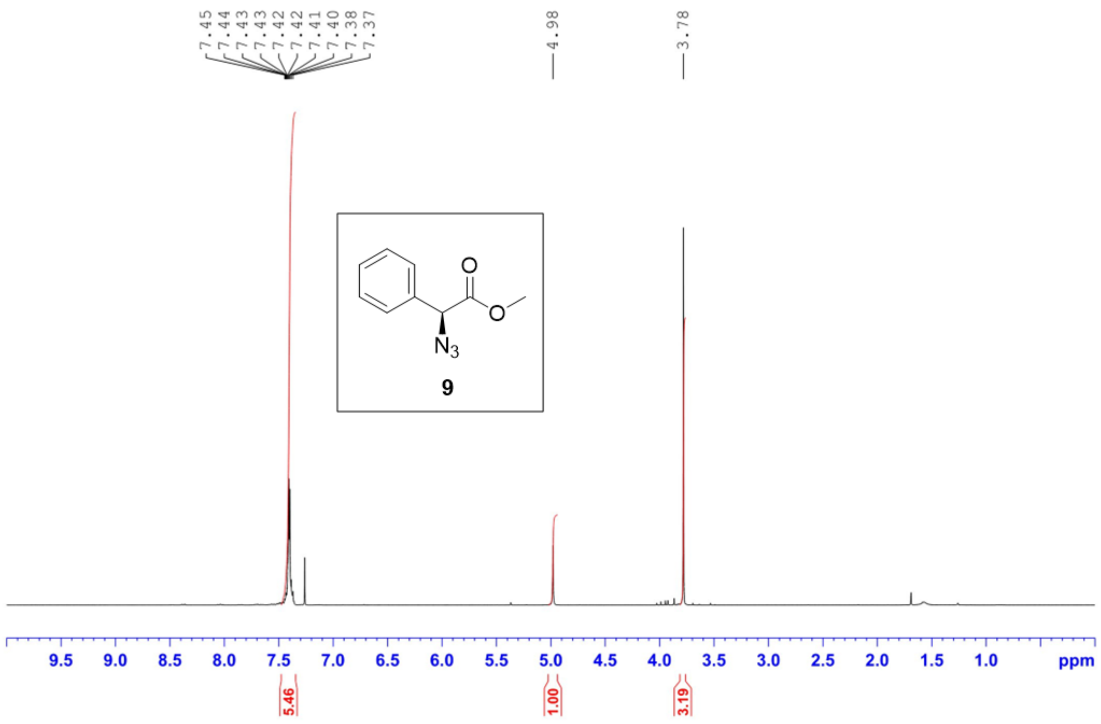

^{13}C-NMR of **9** (75 MHz, $CDCl_3$, 298 K):

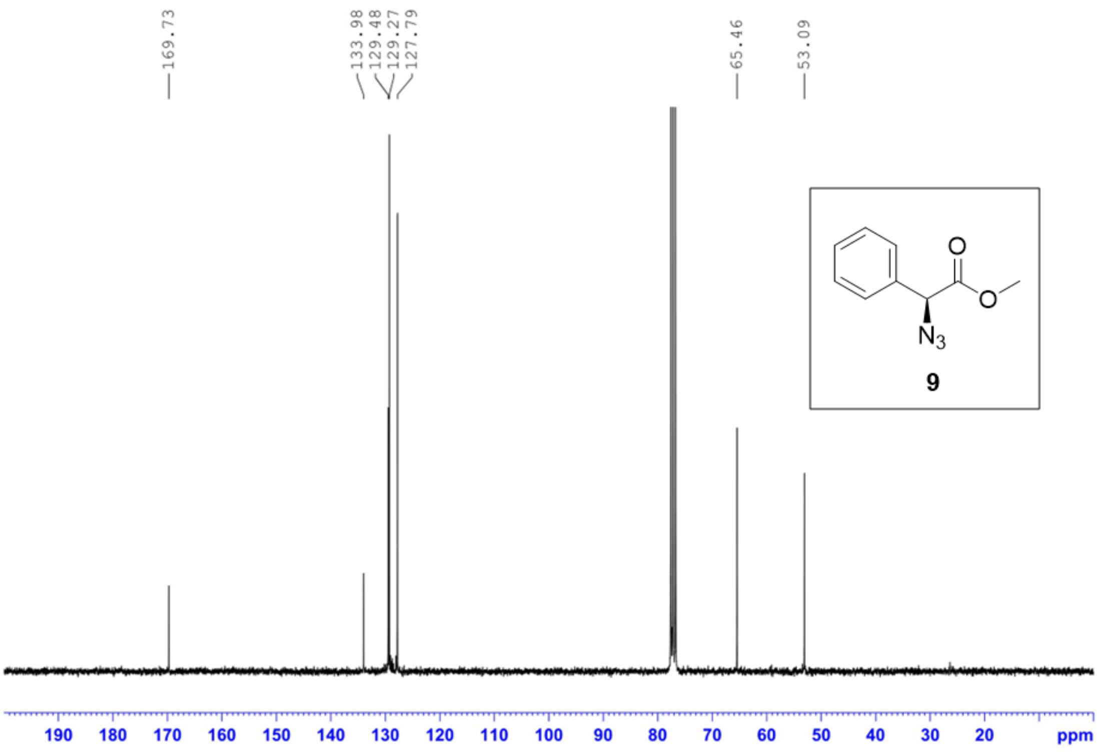

^{1}H-NMR of **10** (300 MHz, $CDCl_3$, 298 K):

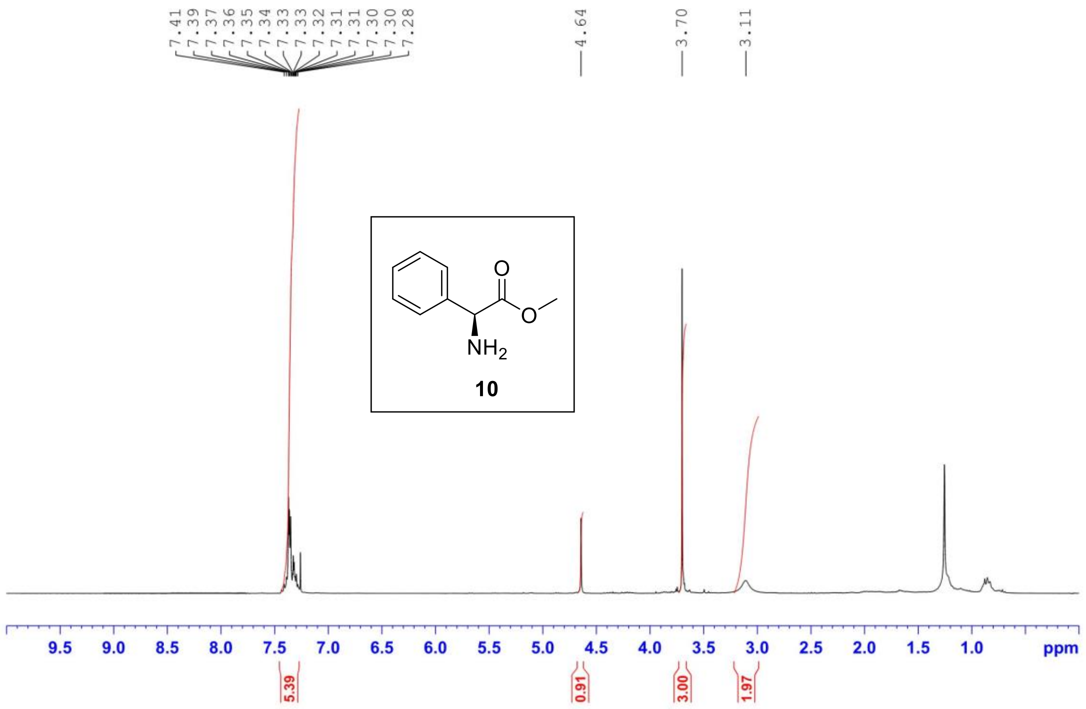

^{13}C-NMR of **10** (75 MHz, $CDCl_3$, 298 K):

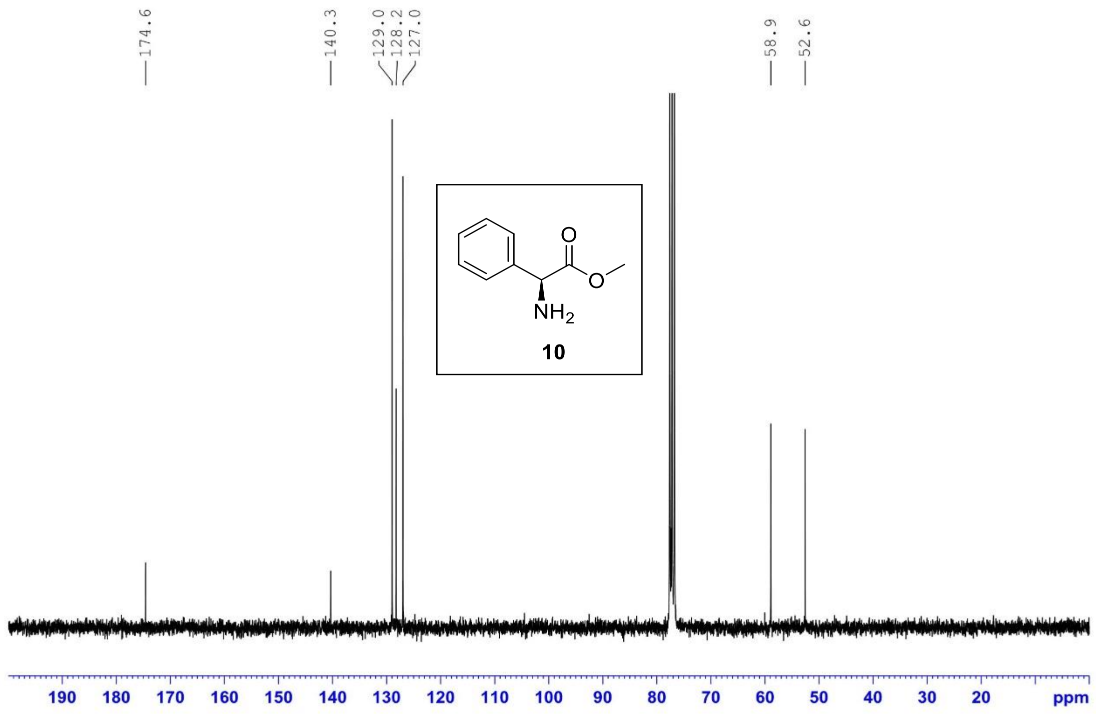

7. Copies of HPLC Chromatograms:

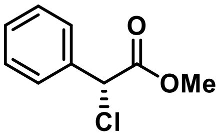

HPLC chromatograms of racemic and enantioenriched **2a**

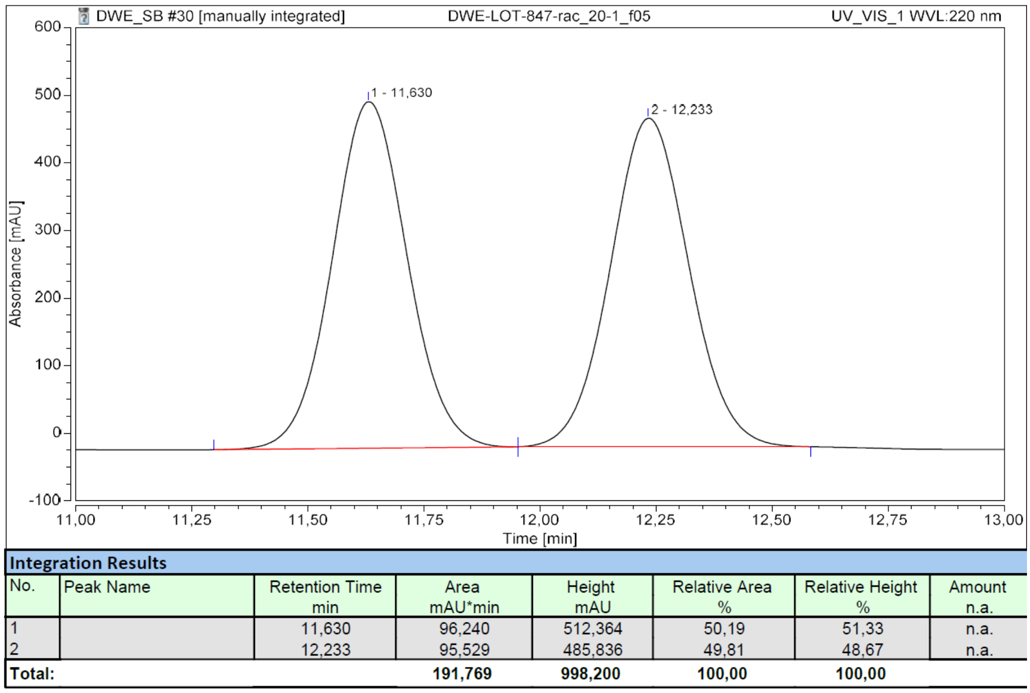

Integration Results

No.	Peak Name	Retention Time min	Area mAU*min	Height mAU	Relative Area %	Relative Height %	Amount n.a.
1		11,630	96,240	512,364	50,19	51,33	n.a.
2		12,233	95,529	485,836	49,81	48,67	n.a.
**Total:**			**191,769**	**998,200**	**100,00**	**100,00**	

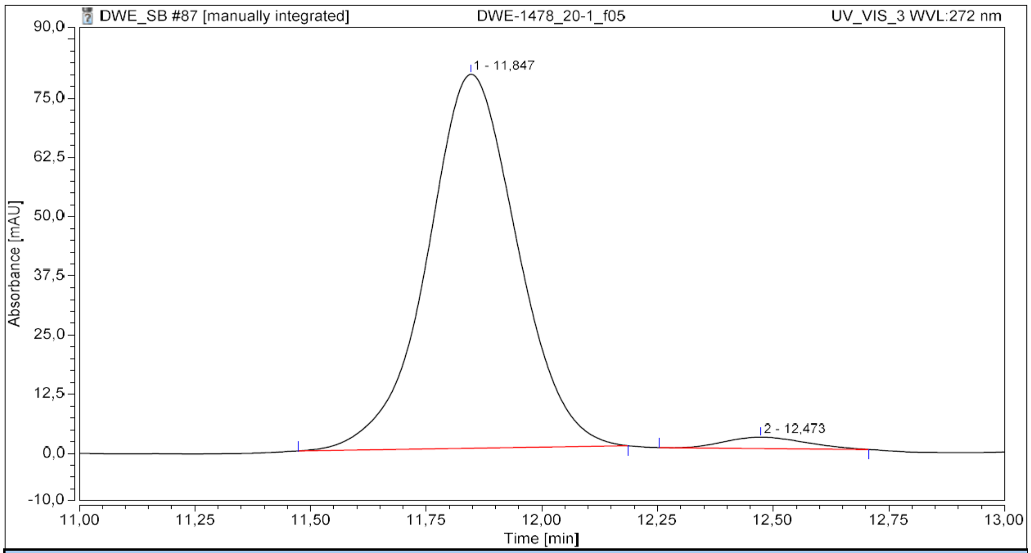

Integration Results

No.	Peak Name	Retention Time min	Area mAU*min	Height mAU	Relative Area %	Relative Height %	Amount n.a.
1		11,847	17,825	78,993	97,38	97,08	n.a.
2		12,473	0,479	2,380	2,62	2,92	n.a.
**Total:**			**18,304**	**81,373**	**100,00**	**100,00**	

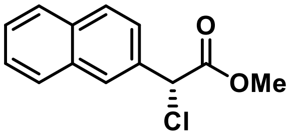

HPLC chromatograms of racemic and enantioenriched **2b**

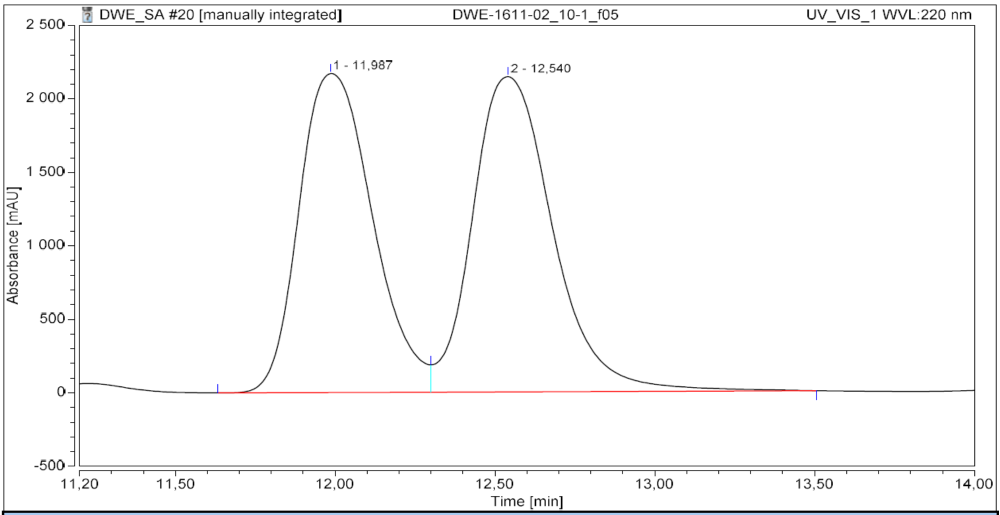

Integration Results							
No.	Peak Name	Retention Time min	Area mAU*min	Height mAU	Relative Area %	Relative Height %	Amount n.a.
1		11,987	578,499	2170,768	48,09	50,30	n.a.
2		12,540	624,431	2144,523	51,91	49,70	n.a.
**Total:**			**1202,930**	**4315,291**	**100,00**	**100,00**	

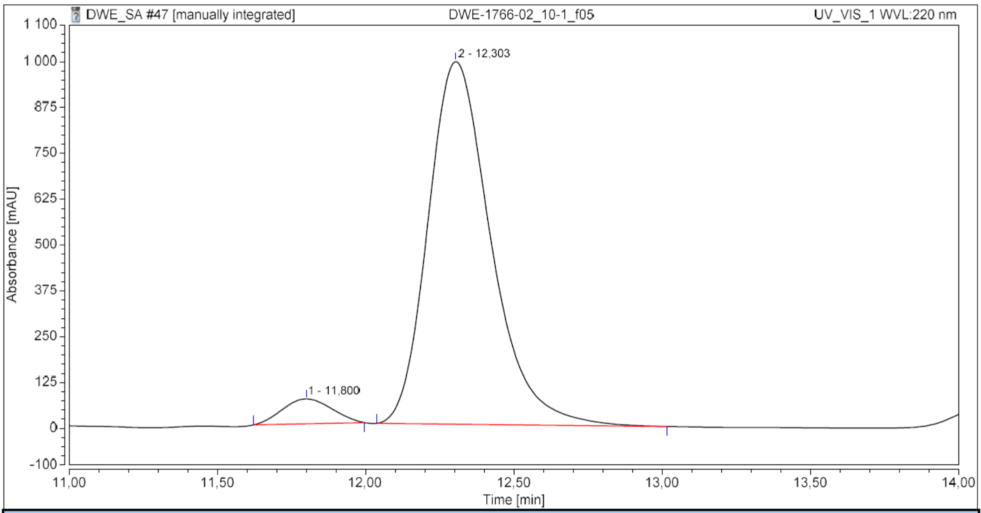

Integration Results							
No.	Peak Name	Retention Time min	Area mAU*min	Height mAU	Relative Area %	Relative Height %	Amount n.a.
1		11,800	13,045	67,989	5,11	6,43	n.a.
2		12,303	242,485	989,078	94,89	93,57	n.a.
**Total:**			**255,530**	**1057,067**	**100,00**	**100,00**	

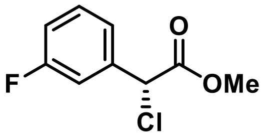

HPLC chromatograms of racemic and enantioenriched **2c**

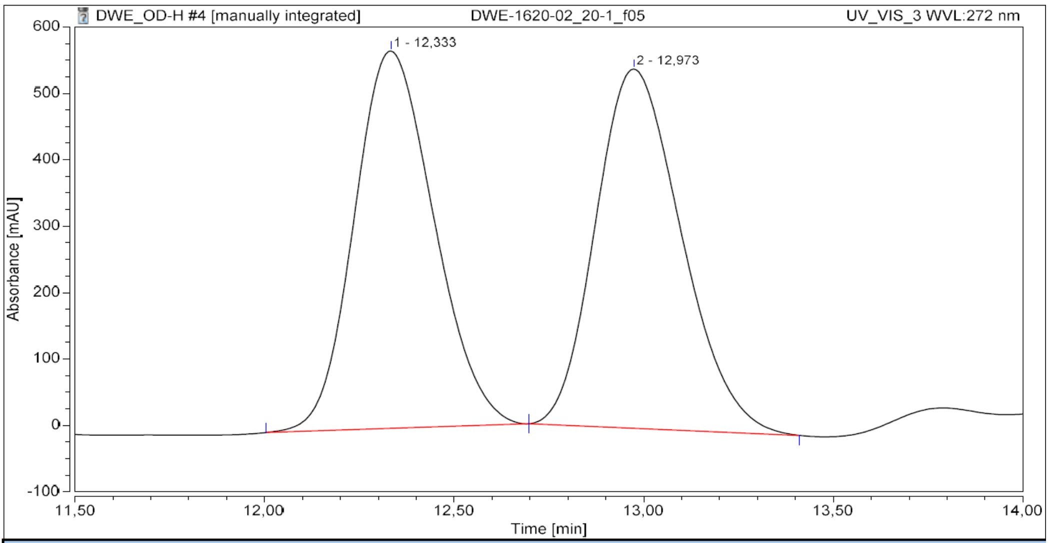

Integration Results

No.	Peak Name	Retention Time min	Area mAU*min	Height mAU	Relative Area %	Relative Height %	Amount n.a.
1		12,333	138,286	568,278	49,37	51,23	n.a.
2		12,973	141,835	540,928	50,63	48,77	n.a.
**Total:**			**280,121**	**1109,207**	**100,00**	**100,00**	

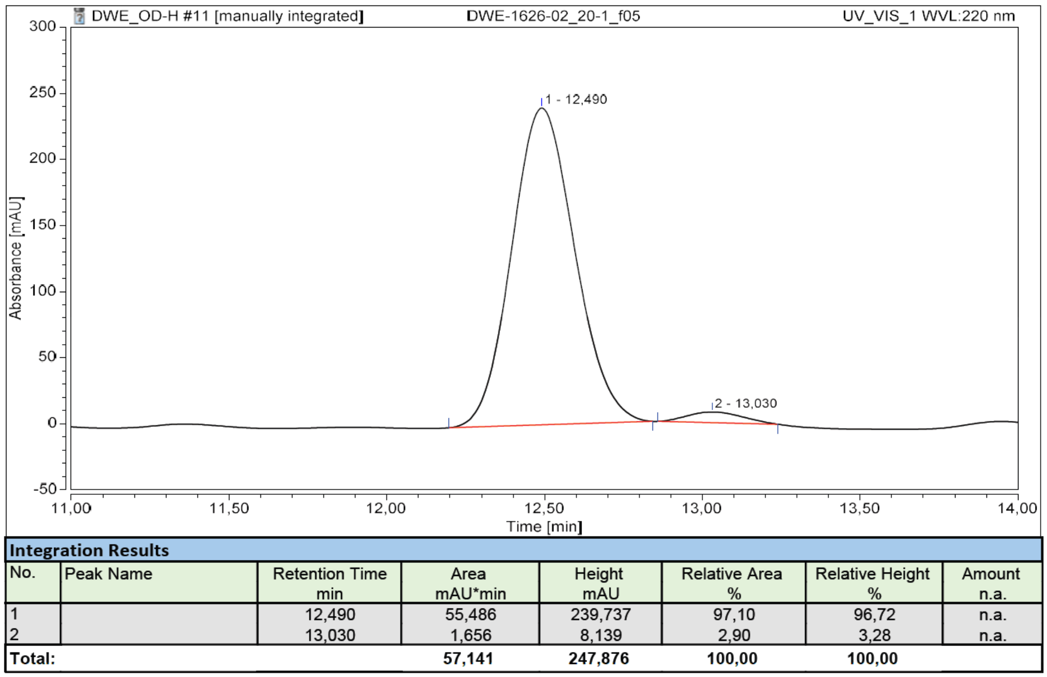

Integration Results

No.	Peak Name	Retention Time min	Area mAU*min	Height mAU	Relative Area %	Relative Height %	Amount n.a.
1		12,490	55,486	239,737	97,10	96,72	n.a.
2		13,030	1,656	8,139	2,90	3,28	n.a.
**Total:**			**57,141**	**247,876**	**100,00**	**100,00**	

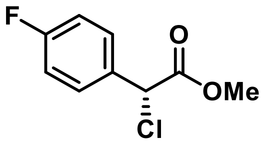

HPLC chromatograms of racemic and enantioenriched **2d**

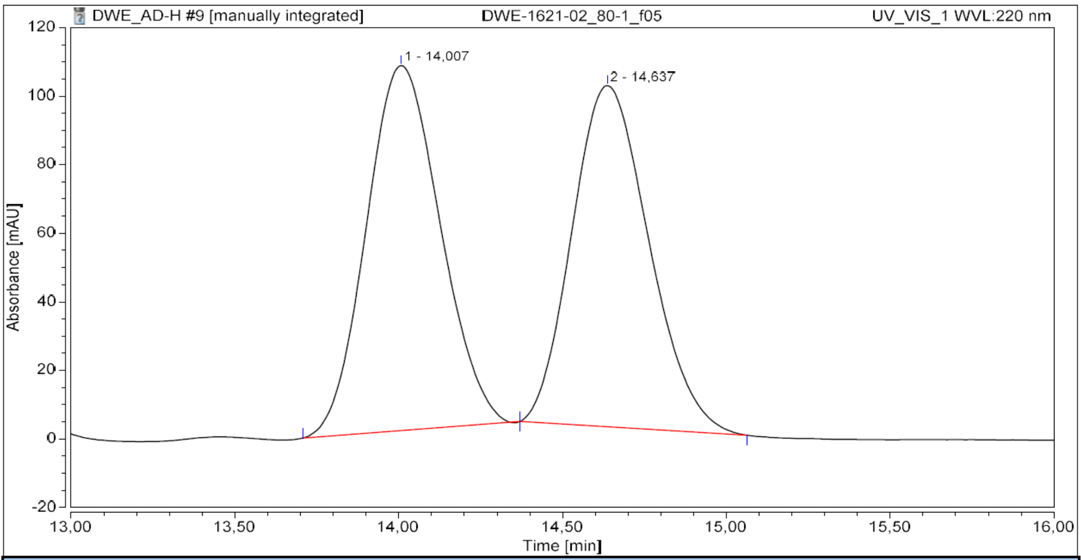

Integration Results							
No.	Peak Name	Retention Time min	Area mAU*min	Height mAU	Relative Area %	Relative Height %	Amount n.a.
1		14,007	27,806	106,477	50,69	51,69	n.a.
2		14,637	27,052	99,519	49,31	48,31	n.a.
**Total:**			**54,857**	**205,996**	**100,00**	**100,00**	

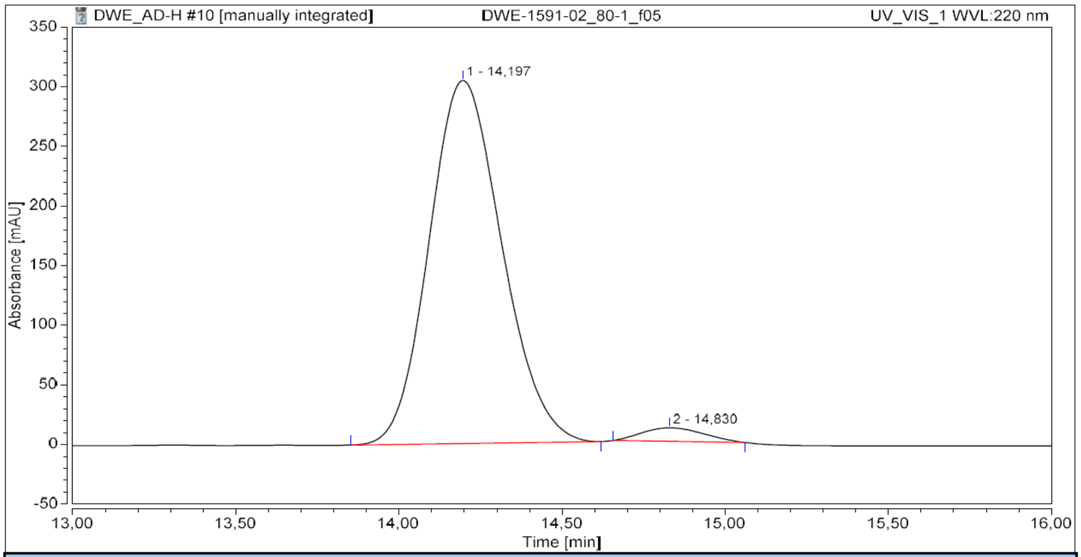

Integration Results							
No.	Peak Name	Retention Time min	Area mAU*min	Height mAU	Relative Area %	Relative Height %	Amount n.a.
1		14,197	78,616	304,608	96,94	96,39	n.a.
2		14,830	2,485	11,415	3,06	3,61	n.a.
**Total:**			**81,101**	**316,023**	**100,00**	**100,00**	

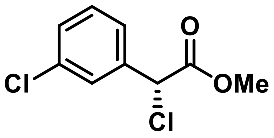

HPLC chromatograms of racemic and enantioenriched **2e**

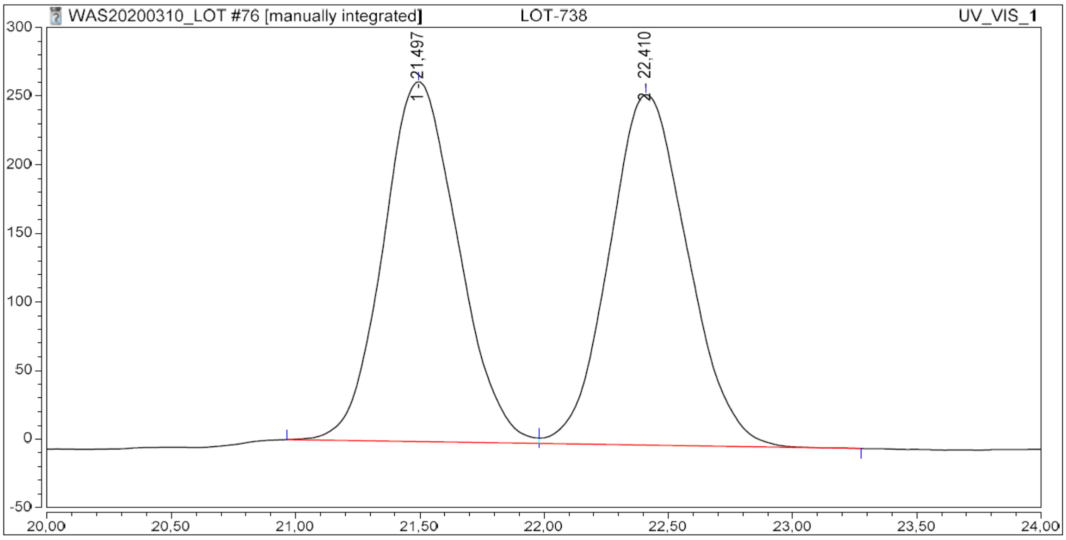

Integration Results							
No.	Peak Name	Retention Time min	Area mAU*min	Height mAU	Relative Area %	Relative Height %	Amount n.a.
1		21,497	91,590	262,318	49,43	50,66	n.a.
2		22,410	93,705	255,451	50,57	49,34	n.a.
			**185,294**	**517,769**	**100,00**	**100,00**	

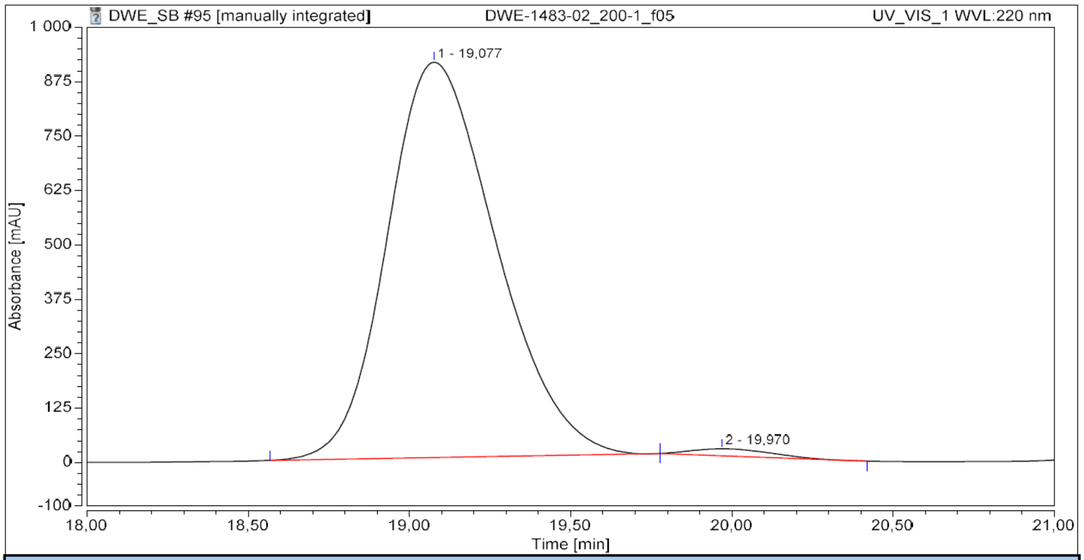

Integration Results							
No.	Peak Name	Retention Time min	Area mAU*min	Height mAU	Relative Area %	Relative Height %	Amount n.a.
1		19,077	354,851	908,101	98,71	98,26	n.a.
2		19,970	4,625	16,083	1,29	1,74	n.a.
**Total:**			**359,476**	**924,184**	**100,00**	**100,00**	

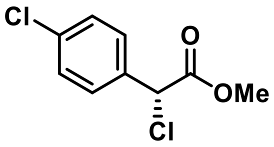

HPLC chromatograms of racemic and enantioenriched **2f**

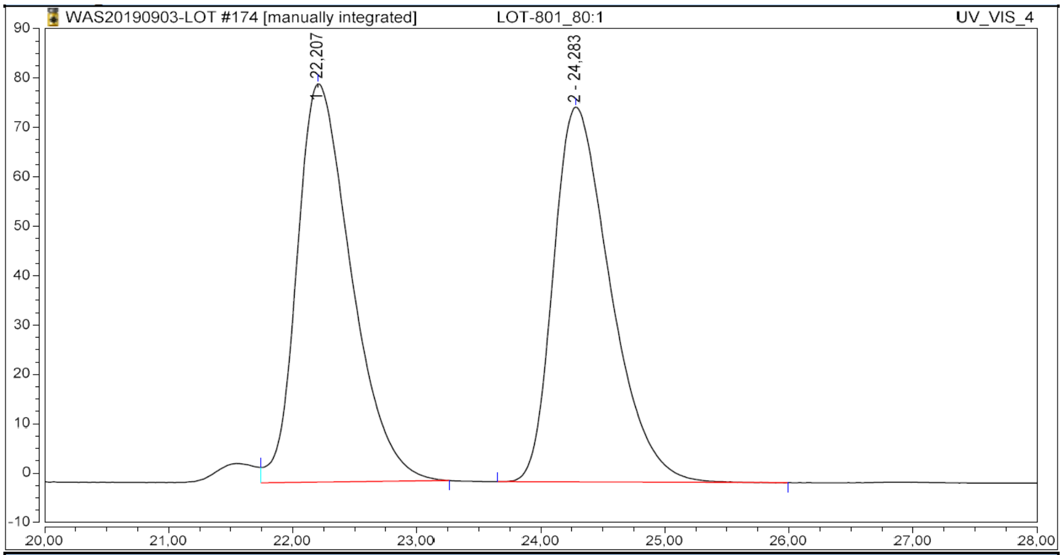

Integration Results

No.	Peak Name	Retention Time min	Area mAU*min	Height mAU	Relative Area %	Relative Height %	Amount n.a.
5		22,207	40,912	80,650	50,33	51,51	n.a.
5		24,283	40,379	75,919	49,67	48,49	n.a.
**Total:**			**81,291**	**156,569**	**100,00**	**100,00**	

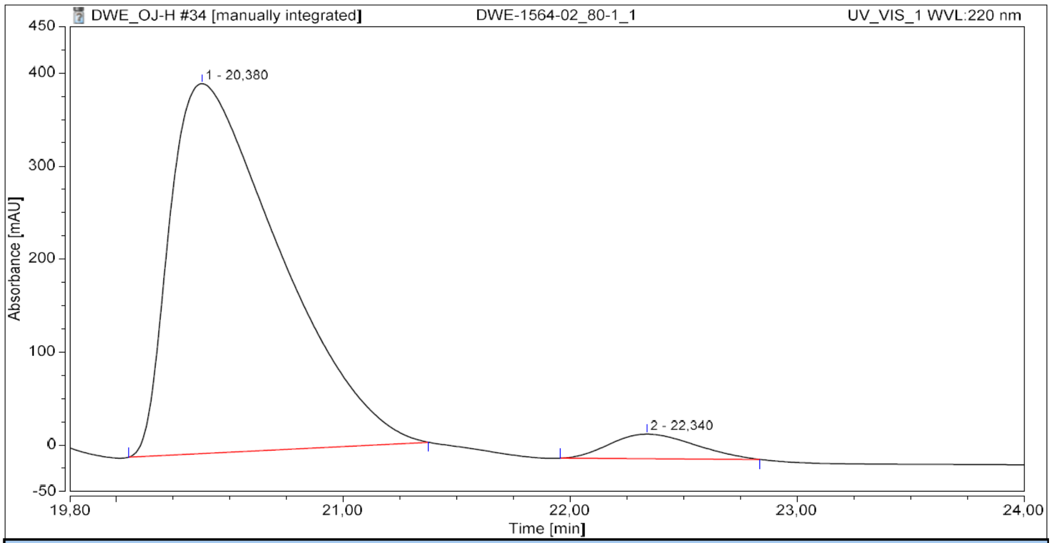

Integration Results

No.	Peak Name	Retention Time min	Area mAU*min	Height mAU	Relative Area %	Relative Height %	Amount n.a.
1		20,380	222,800	397,683	95,09	93,75	n.a.
2		22,340	11,514	26,517	4,91	6,25	n.a.
**Total:**			**234,315**	**424,199**	**100,00**	**100,00**	

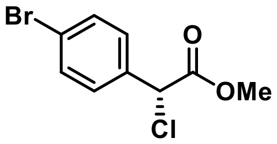

HPLC chromatograms of racemic and enantioenriched **2g**

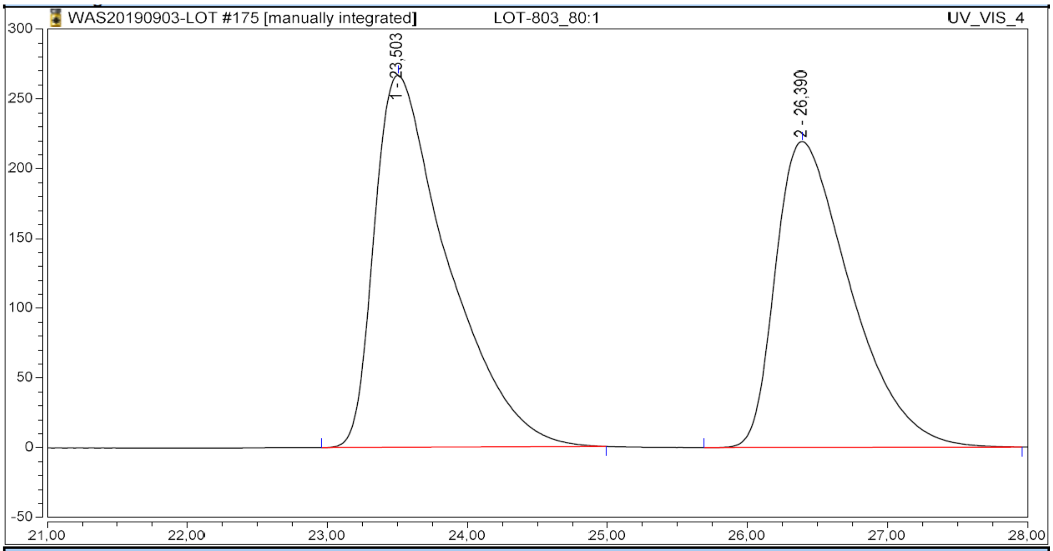

Integration Results

No.	Peak Name	Retention Time min	Area mAU*min	Height mAU	Relative Area %	Relative Height %	Amount n.a.
5		23,503	162,164	266,625	54,41	54,88	n.a.
5		26,390	135,868	219,189	45,59	45,12	n.a.
**Total:**			**298,032**	**485,814**	**100,00**	**100,00**	

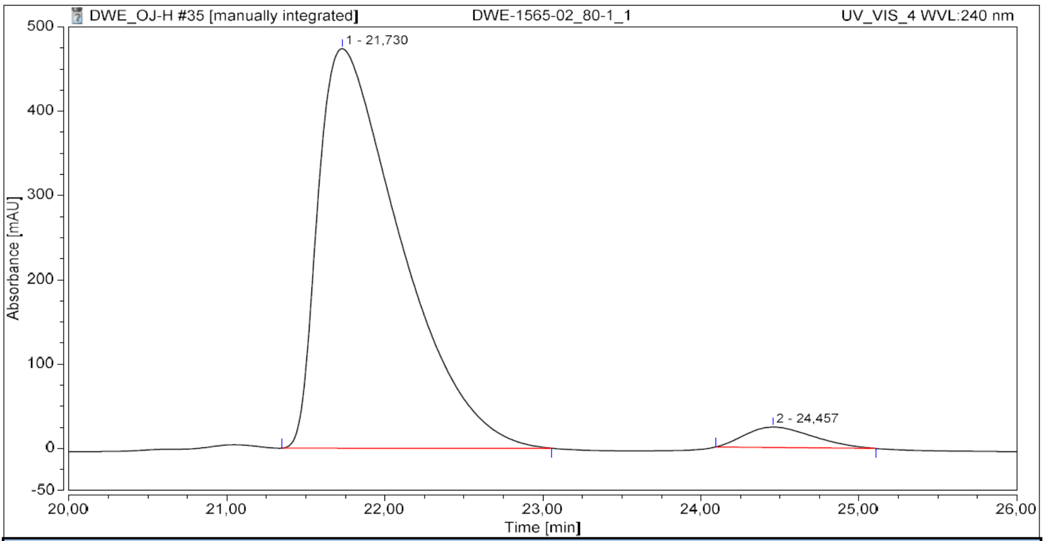

Integration Results

No.	Peak Name	Retention Time min	Area mAU*min	Height mAU	Relative Area %	Relative Height %	Amount n.a.
1		21,730	286,872	473,886	95,95	95,14	n.a.
2		24,457	12,104	24,216	4,05	4,86	n.a.
**Total:**			**298,976**	**498,102**	**100,00**	**100,00**	

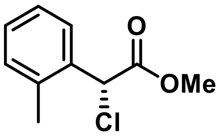

HPLC chromatograms of racemic and enantioenriched **2h**

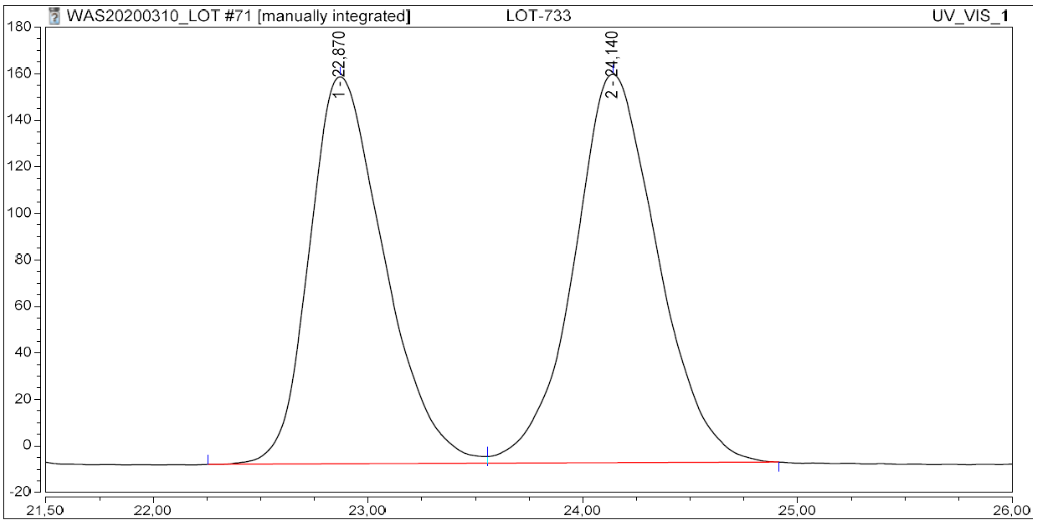

Integration Results							
No.	Peak Name	Retention Time min	Area mAU*min	Height mAU	Relative Area %	Relative Height %	Amount n.a.
1		22,870	67,272	166,438	47,11	49,92	n.a.
2		24,140	75,515	166,980	52,89	50,08	n.a.
			**142,787**	**333,418**	**100,00**	**100,00**	

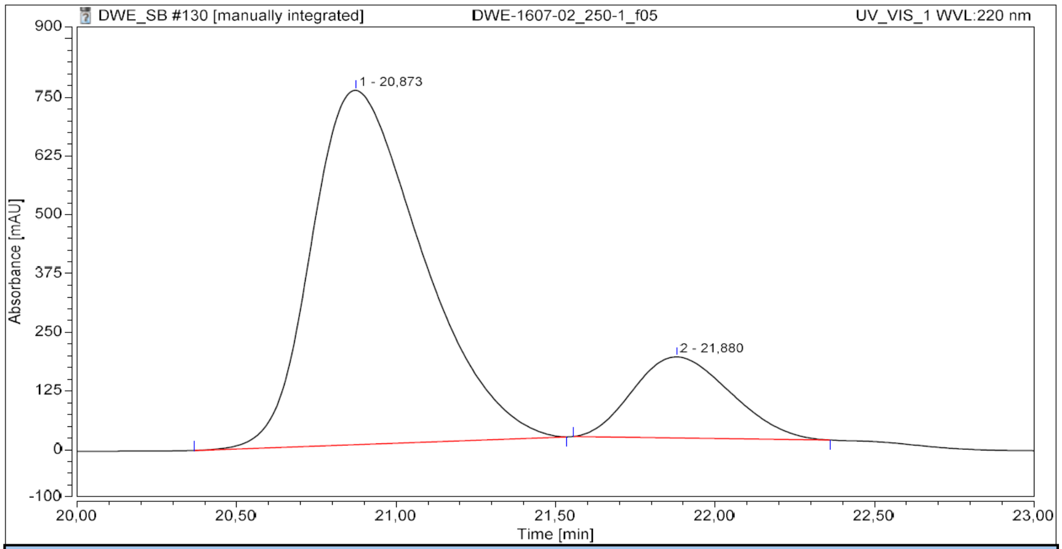

Integration Results							
No.	Peak Name	Retention Time min	Area mAU*min	Height mAU	Relative Area %	Relative Height %	Amount n.a.
1		20,873	302,941	753,764	83,22	81,39	n.a.
2		21,880	61,089	172,404	16,78	18,61	n.a.
**Total:**			**364,030**	**926,168**	**100,00**	**100,00**	

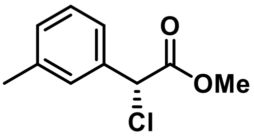

HPLC chromatograms of racemic and enantioenriched **2i**

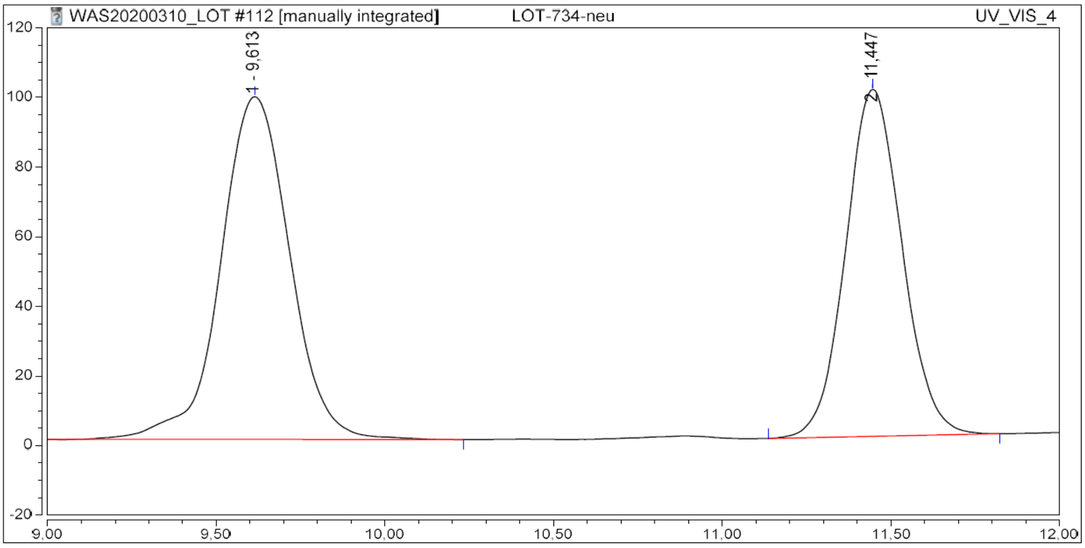

Integration Results							
No.	Peak Name	Retention Time min	Area mAU*min	Height mAU	Relative Area %	Relative Height %	Amount n.a.
1		9,613	23,320	98,457	54,68	49,69	n.a.
2		11,447	19,329	99,686	45,32	50,31	n.a.
			**42,650**	**198,143**	**100,00**	**100,00**	

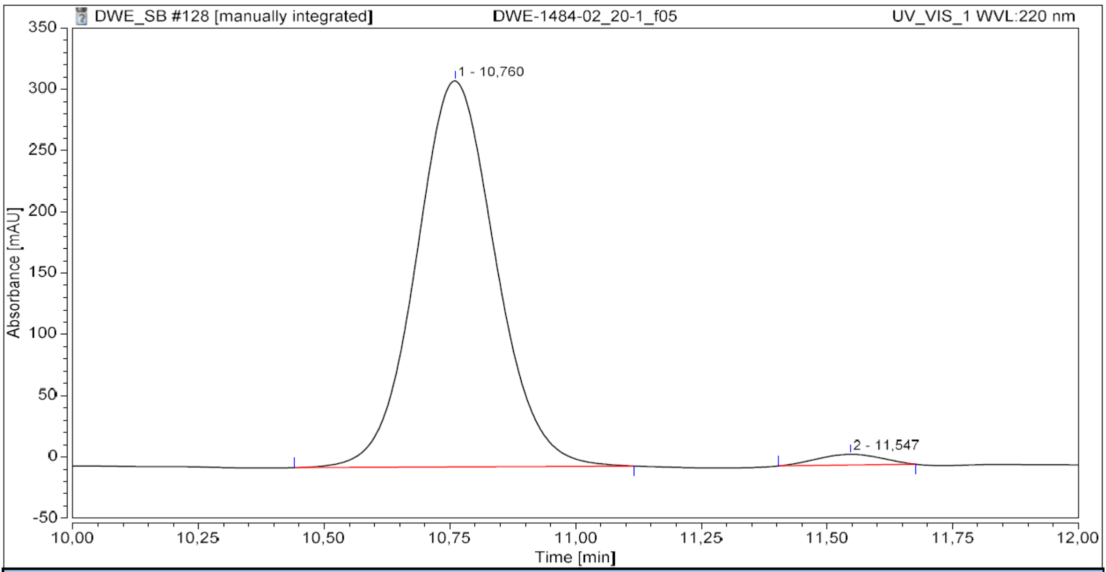

Integration Results							
No.	Peak Name	Retention Time min	Area mAU*min	Height mAU	Relative Area %	Relative Height %	Amount n.a.
1		10,760	57,715	314,965	97,78	97,29	n.a.
2		11,547	1,309	8,782	2,22	2,71	n.a.
**Total:**			**59,025**	**323,747**	**100,00**	**100,00**	

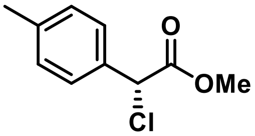

HPLC chromatograms of racemic and enantioenriched **2j**

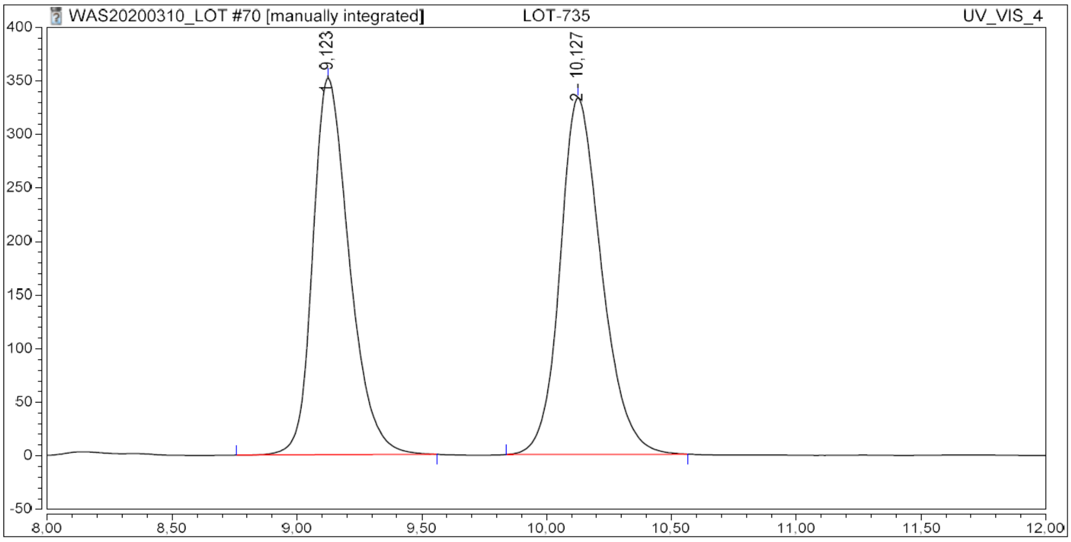

Integration Results							
No.	Peak Name	Retention Time min	Area mAU*min	Height mAU	Relative Area %	Relative Height %	Amount n.a.
1		9,123	59,699	352,016	48,12	51,38	n.a.
2		10,127	64,359	333,075	51,88	48,62	n.a.
			**124,058**	**685,091**	**100,00**	**100,00**	

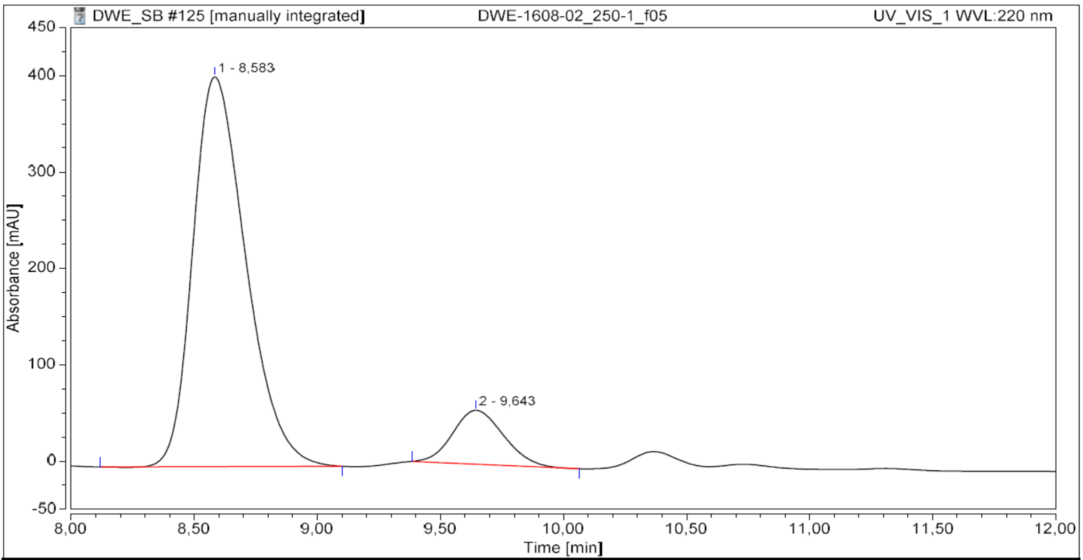

Integration Results							
No.	Peak Name	Retention Time min	Area mAU*min	Height mAU	Relative Area %	Relative Height %	Amount n.a.
1		8,583	100,889	404,451	88,27	87,86	n.a.
2		9,643	13,409	55,894	11,73	12,14	n.a.
**Total:**			**114,298**	**460,344**	**100,00**	**100,00**	

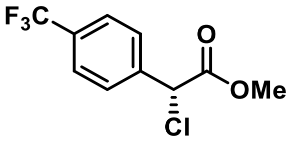

HPLC chromatograms of racemic and enantioenriched **2k**

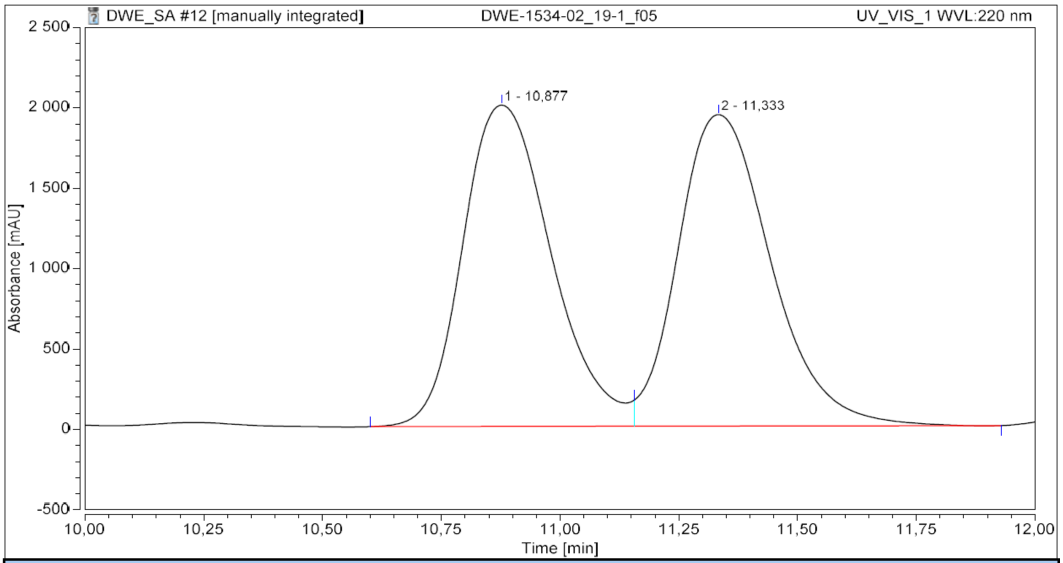

Integration Results							
No.	Peak Name	Retention Time min	Area mAU*min	Height mAU	Relative Area %	Relative Height %	Amount n.a.
1		10,877	431,321	1998,658	49,61	50,78	n.a.
2		11,333	438,085	1937,343	50,39	49,22	n.a.
**Total:**			**869,406**	**3936,001**	**100,00**	**100,00**	

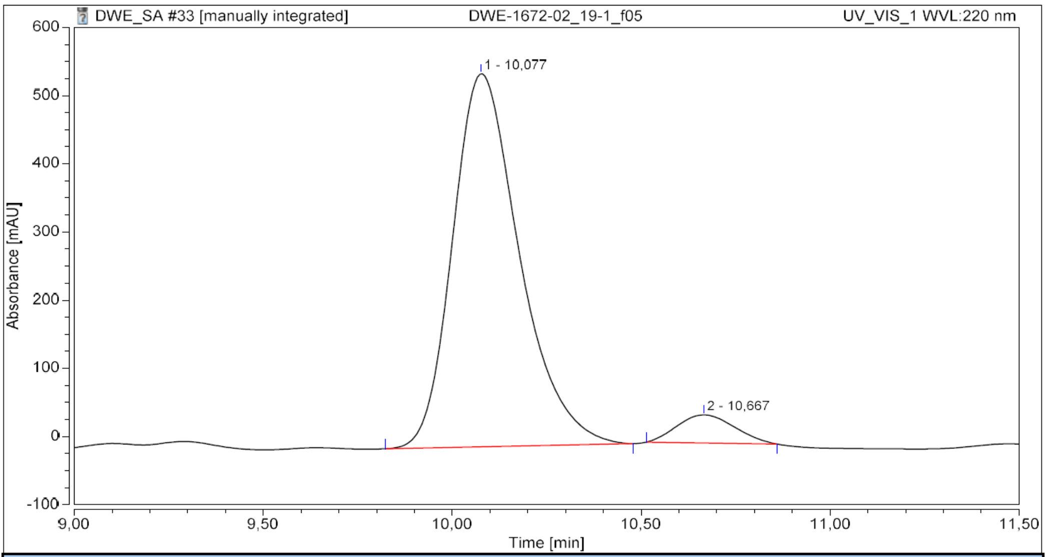

Integration Results							
No.	Peak Name	Retention Time min	Area mAU*min	Height mAU	Relative Area %	Relative Height %	Amount n.a.
1		10,077	112,093	547,051	93,92	93,00	n.a.
2		10,667	7,255	41,196	6,08	7,00	n.a.
**Total:**			**119,348**	**588,247**	**100,00**	**100,00**	

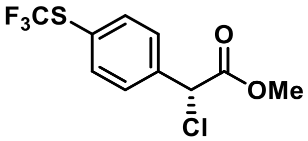

HPLC chromatograms of racemic and enantioenriched **2l**

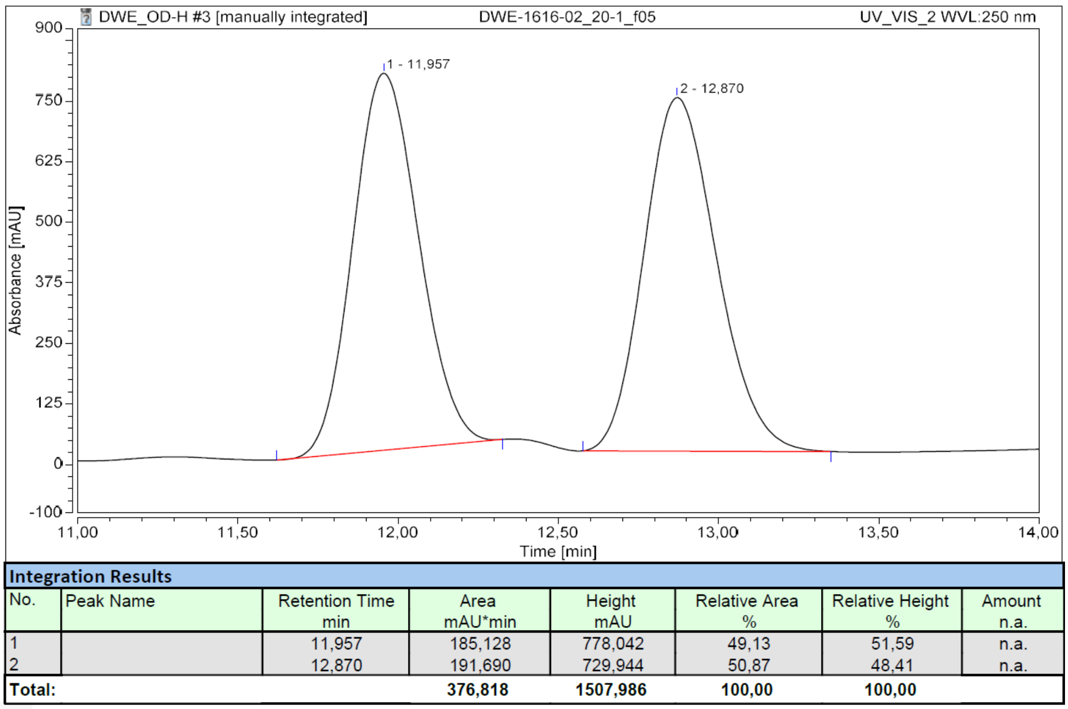

Integration Results

No.	Peak Name	Retention Time min	Area mAU*min	Height mAU	Relative Area %	Relative Height %	Amount n.a.
1		11,957	185,128	778,042	49,13	51,59	n.a.
2		12,870	191,690	729,944	50,87	48,41	n.a.
**Total:**			**376,818**	**1507,986**	**100,00**	**100,00**	

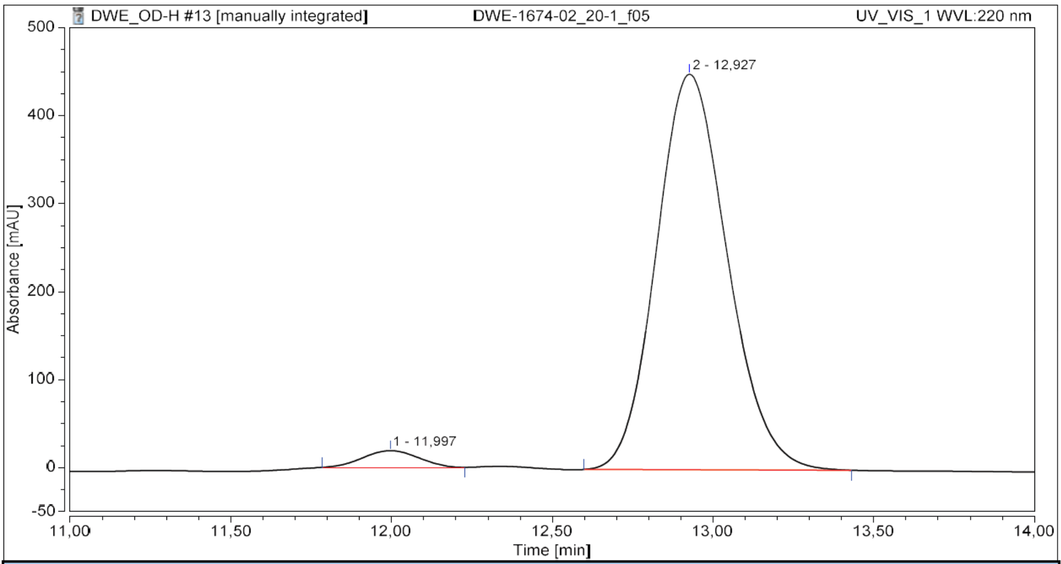

Integration Results

No.	Peak Name	Retention Time min	Area mAU*min	Height mAU	Relative Area %	Relative Height %	Amount n.a.
1		11,997	3,785	18,961	3,18	4,05	n.a.
2		12,927	115,080	449,511	96,82	95,95	n.a.
**Total:**			**118,865**	**468,472**	**100,00**	**100,00**	

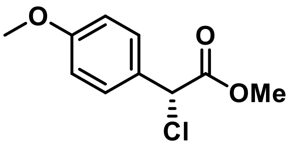

HPLC chromatograms of racemic and enantioenriched **2m**

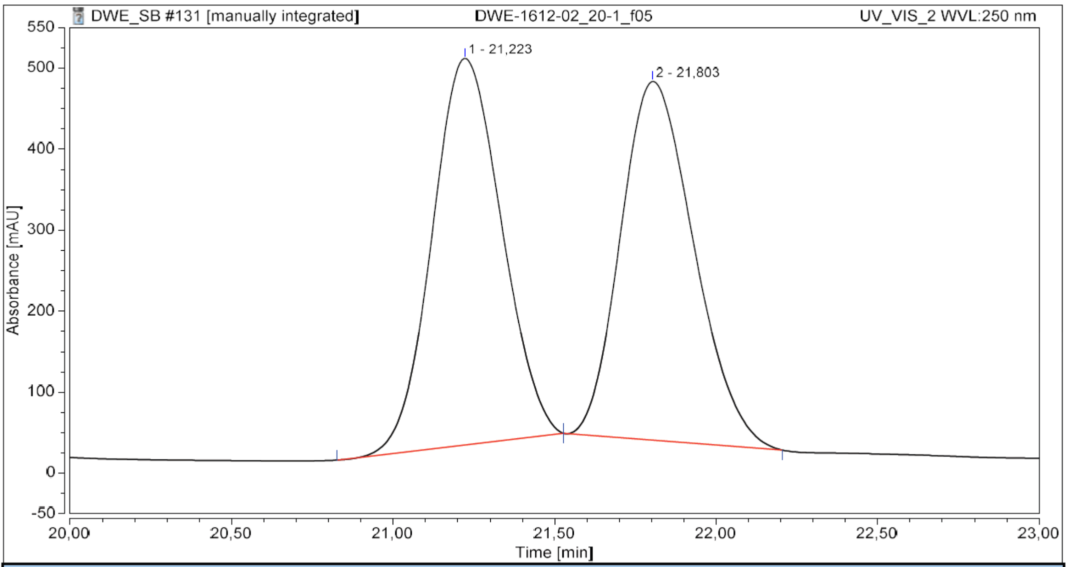

Integration Results							
No.	Peak Name	Retention Time min	Area mAU*min	Height mAU	Relative Area %	Relative Height %	Amount n.a.
1		21,223	118,432	477,041	50,47	51,86	n.a.
2		21,803	116,241	442,794	49,53	48,14	n.a.
**Total:**			**234,674**	**919,835**	**100,00**	**100,00**	

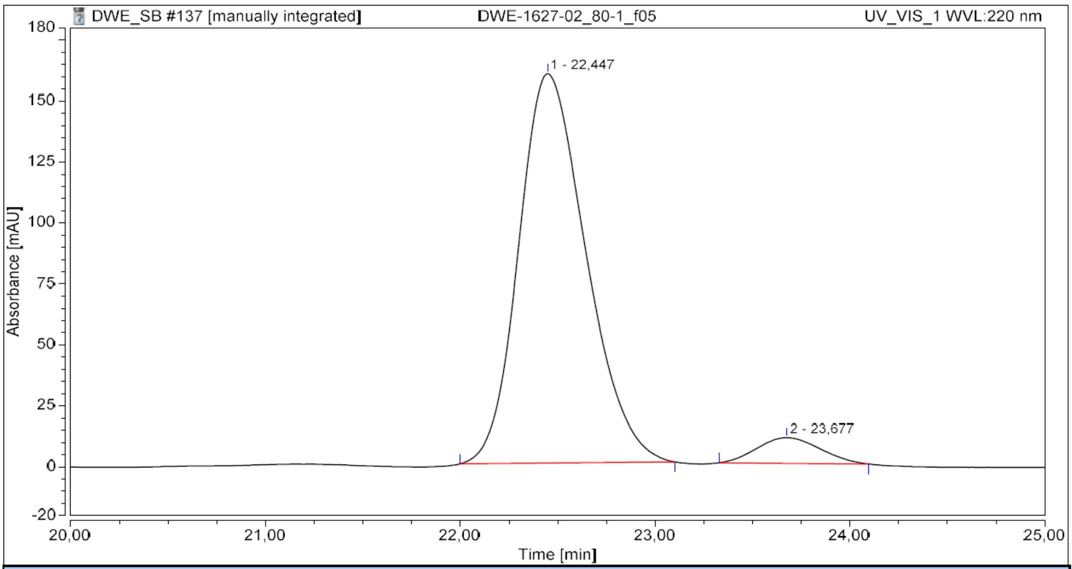

Integration Results							
No.	Peak Name	Retention Time min	Area mAU*min	Height mAU	Relative Area %	Relative Height %	Amount n.a.
1		22,447	63,770	159,682	94,20	93,84	n.a.
2		23,677	3,927	10,487	5,80	6,16	n.a.
**Total:**			**67,697**	**170,169**	**100,00**	**100,00**	

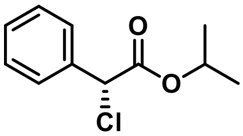

HPLC chromatograms of racemic and enantioenriched **2n**

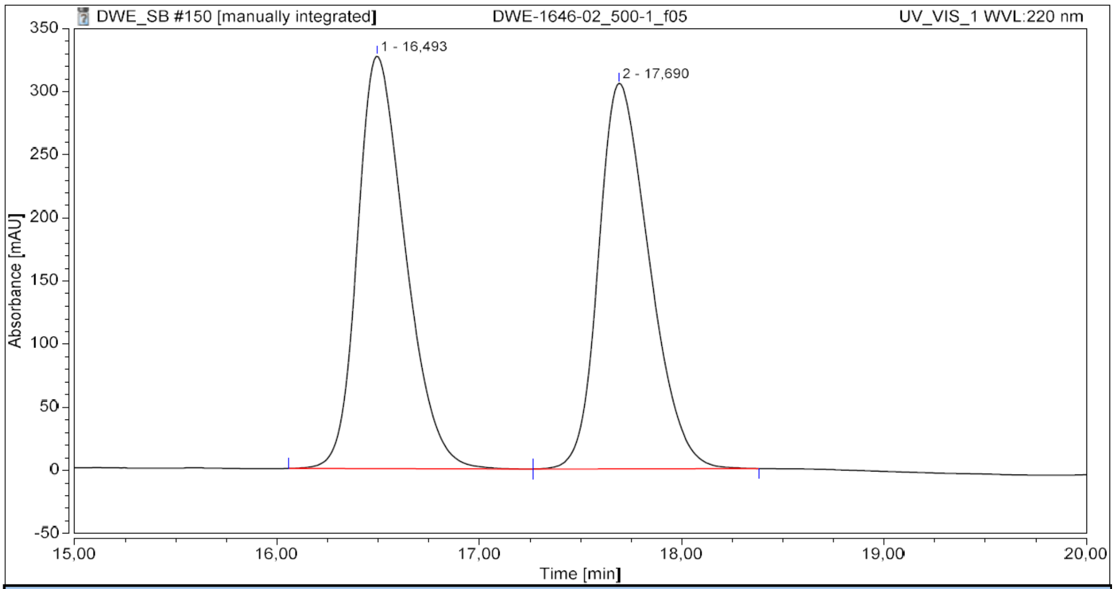

Integration Results

No.	Peak Name	Retention Time min	Area mAU*min	Height mAU	Relative Area %	Relative Height %	Amount n.a.
1		16,493	89,613	326,782	50,27	51,68	n.a.
2		17,690	88,658	305,502	49,73	48,32	n.a.
**Total:**			**178,271**	**632,284**	**100,00**	**100,00**	

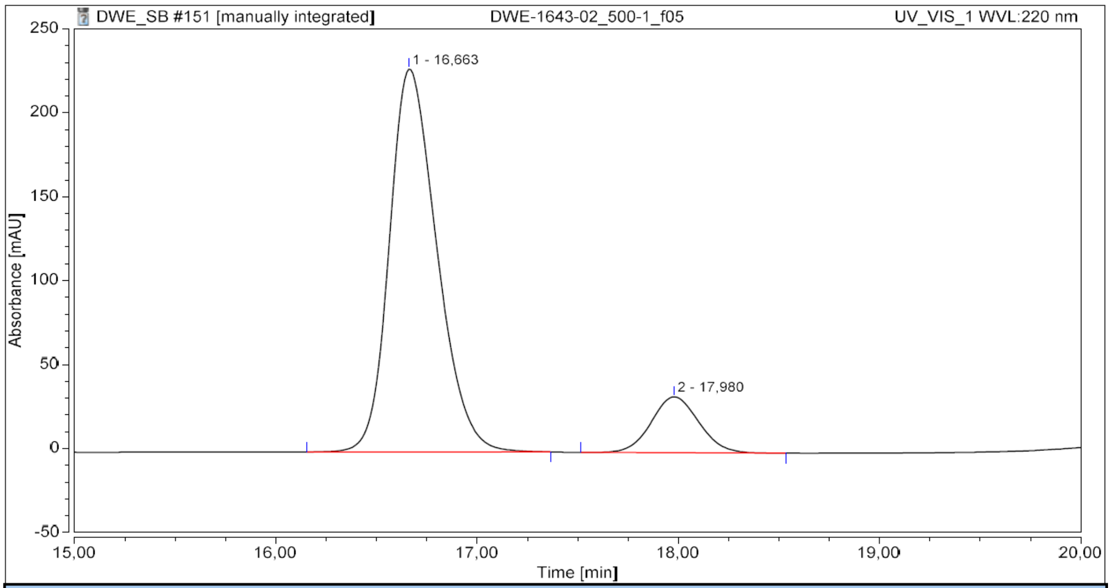

Integration Results

No.	Peak Name	Retention Time min	Area mAU*min	Height mAU	Relative Area %	Relative Height %	Amount n.a.
1		16,663	62,285	228,102	87,20	87,25	n.a.
2		17,980	9,140	33,324	12,80	12,75	n.a.
**Total:**			**71,424**	**261,426**	**100,00**	**100,00**	

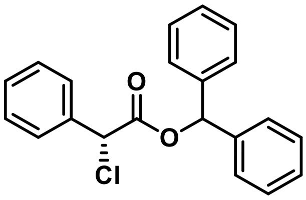

HPLC chromatograms of racemic and enantioenriched **2o**

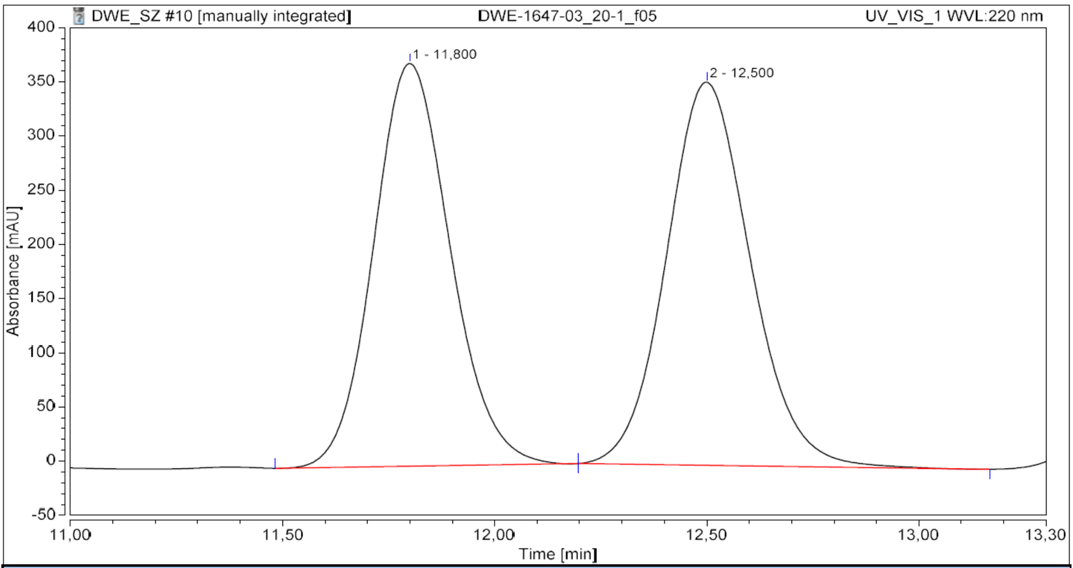

Integration Results							
No.	Peak Name	Retention Time min	Area mAU*min	Height mAU	Relative Area %	Relative Height %	Amount n.a.
1		11,800	77,973	371,637	48,92	51,24	n.a.
2		12,500	81,401	353,666	51,08	48,76	n.a.
**Total:**			**159,375**	**725,303**	**100,00**	**100,00**	

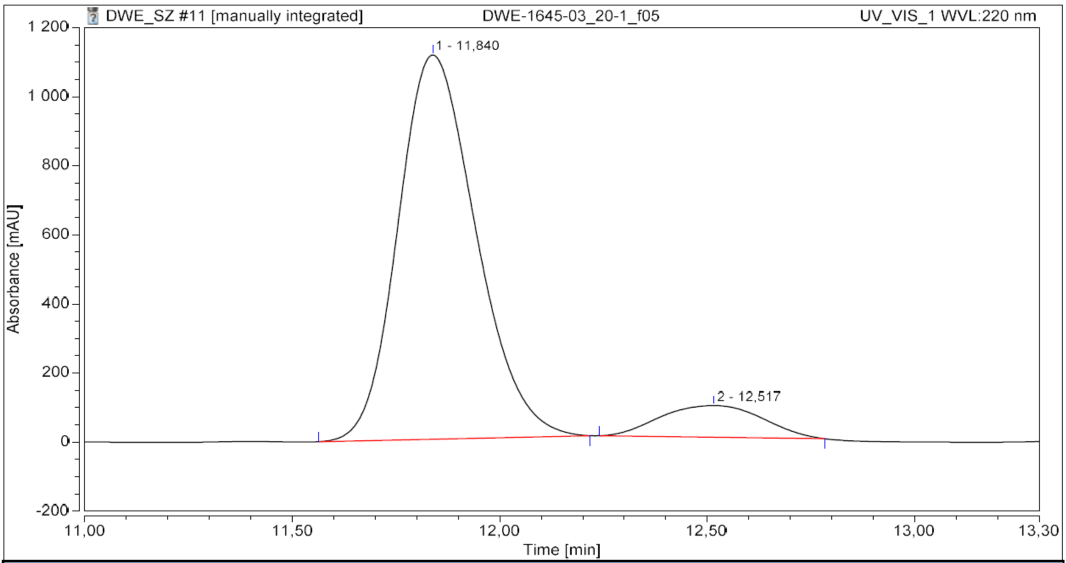

Integration Results							
No.	Peak Name	Retention Time min	Area mAU*min	Height mAU	Relative Area %	Relative Height %	Amount n.a.
1		11,840	241,909	1111,983	90,23	92,38	n.a.
2		12,517	26,201	91,754	9,77	7,62	n.a.
**Total:**			**268,110**	**1203,737**	**100,00**	**100,00**	

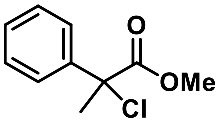

HPLC chromatograms of racemic and enantioenriched **4**

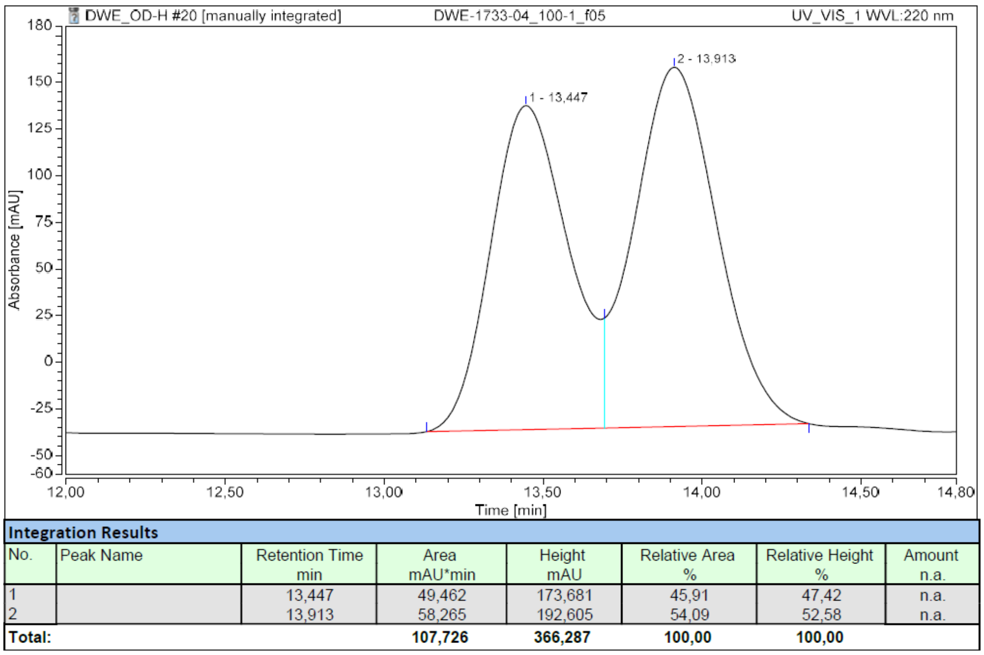

Integration Results							
No.	Peak Name	Retention Time min	Area mAU*min	Height mAU	Relative Area %	Relative Height %	Amount n.a.
1		13,447	49,462	173,681	45,91	47,42	n.a.
2		13,913	58,265	192,605	54,09	52,58	n.a.
**Total:**			**107,726**	**366,287**	**100,00**	**100,00**	

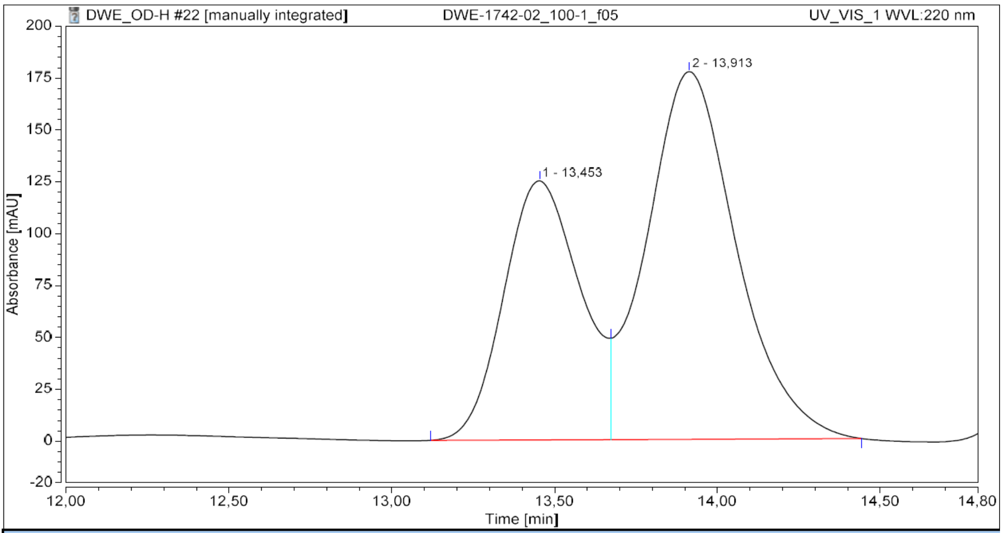

Integration Results							
No.	Peak Name	Retention Time min	Area mAU*min	Height mAU	Relative Area %	Relative Height %	Amount n.a.
1		13,453	34,136	124,837	37,59	41,33	n.a.
2		13,913	56,667	177,188	62,41	58,67	n.a.
**Total:**			**90,803**	**302,025**	**100,00**	**100,00**	

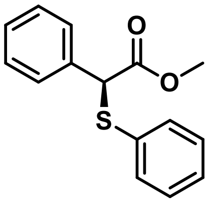

HPLC chromatograms of racemic and enantioenriched **7**

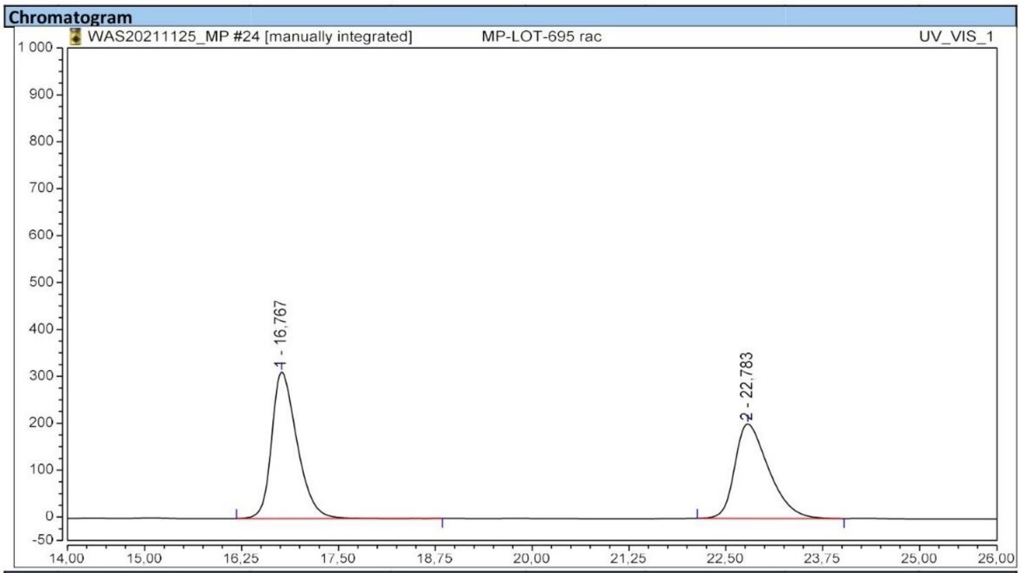

Integration Results

No.	Peak Name	Retention Time min	Area mAU*min	Height mAU	Relative Area %	Relative Height %	Amount n.a.
1		16,767	118,586	312,195	53,74	60,80	n.a.
2		22,783	102,070	201,289	46,26	39,20	n.a.
**Total:**			**220,656**	**513,484**	**100,00**	**100,00**	

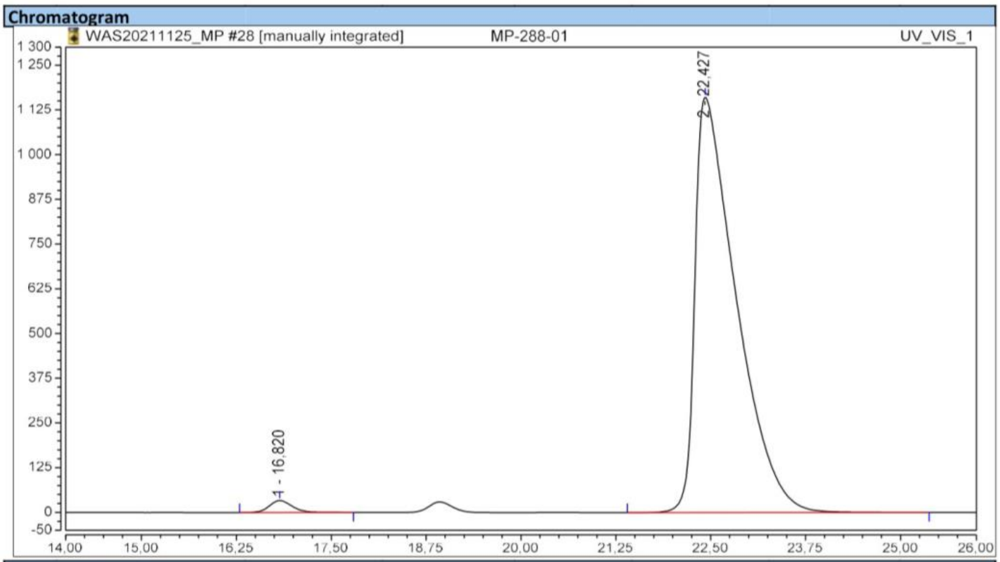

Integration Results

No.	Peak Name	Retention Time min	Area mAU*min	Height mAU	Relative Area %	Relative Height %	Amount n.a.
1		16,820	11,743	33,438	1,58	2,80	n.a.
2		22,427	731,830	1161,268	98,42	97,20	n.a.
**Total:**			**743,573**	**1194,706**	**100,00**	**100,00**	

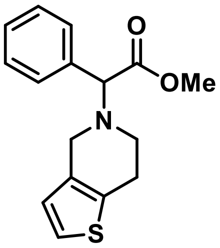

HPLC chromatograms of racemic and enantioenriched **8**

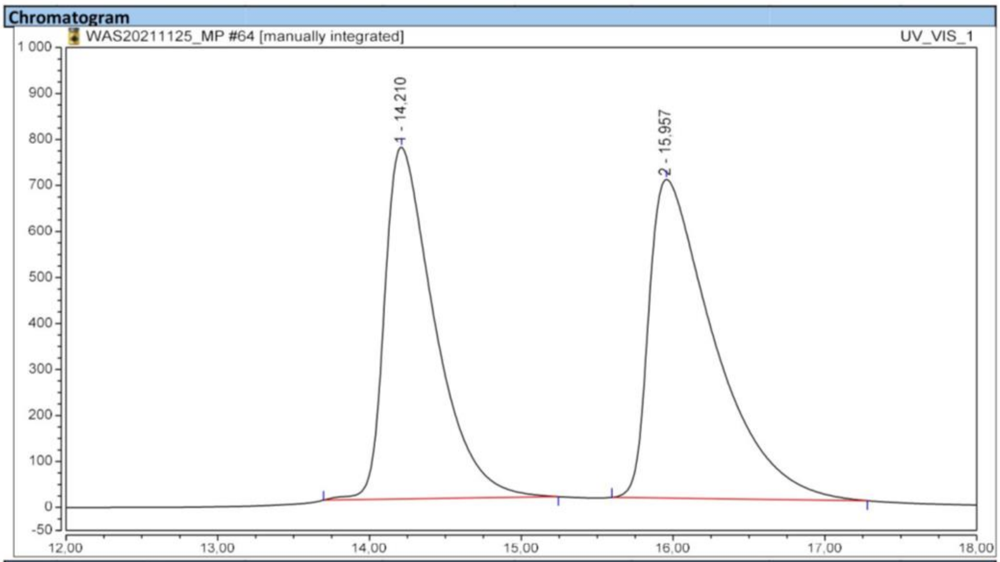

Integration Results

No.	Peak Name	Retention Time min	Area mAU*min	Height mAU	Relative Area %	Relative Height %	Amount n.a.
1		14,210	292,026	764,679	45,78	52,47	n.a.
2		15,957	345,841	692,764	54,22	47,53	n.a.
**Total:**			**637,867**	**1457,443**	**100,00**	**100,00**	

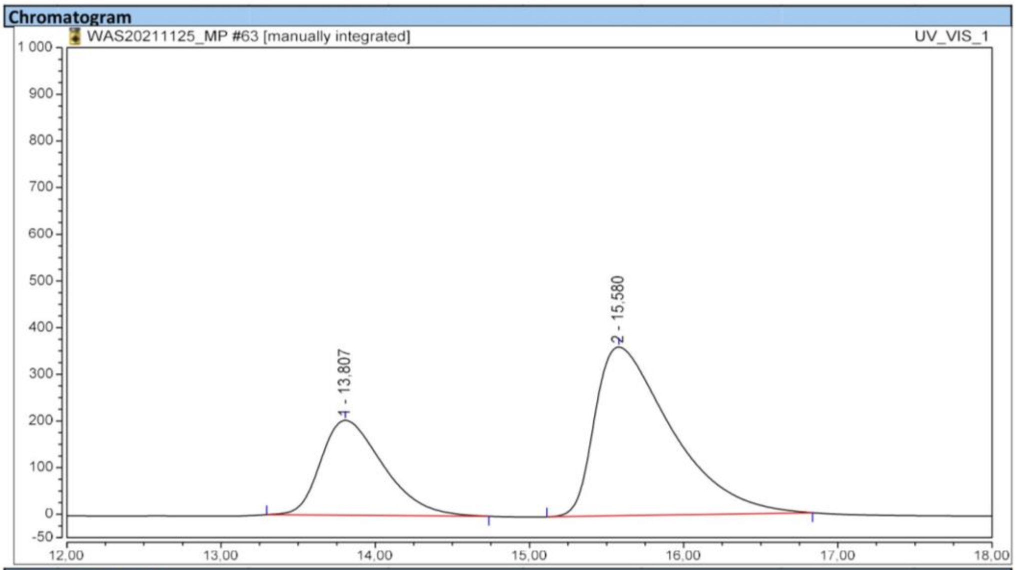

Integration Results

No.	Peak Name	Retention Time min	Area mAU*min	Height mAU	Relative Area %	Relative Height %	Amount n.a.
1		13,807	95,445	203,196	31,40	35,98	n.a.
2		15,580	208,524	361,517	68,60	64,02	n.a.
**Total:**			**303,969**	**564,713**	**100,00**	**100,00**	

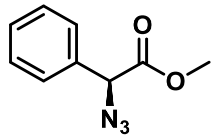

HPLC chromatograms of racemic and enantioenriched **9**

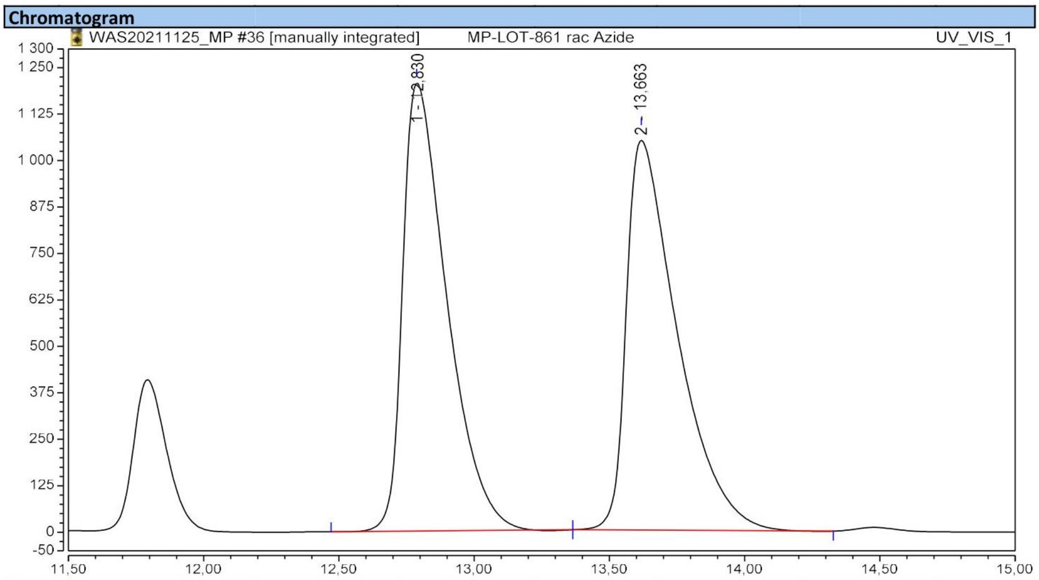

Integration Results

No.	Peak Name	Retention Time min	Area mAU*min	Height mAU	Relative Area %	Relative Height %	Amount n.a.
1		13,527	357,705	1205,378	49,80	53,47	n.a.
2		14,830	360,575	1048,858	50,20	46,53	n.a.
**Total:**			**718,280**	**2254,236**	**100,00**	**100,00**	

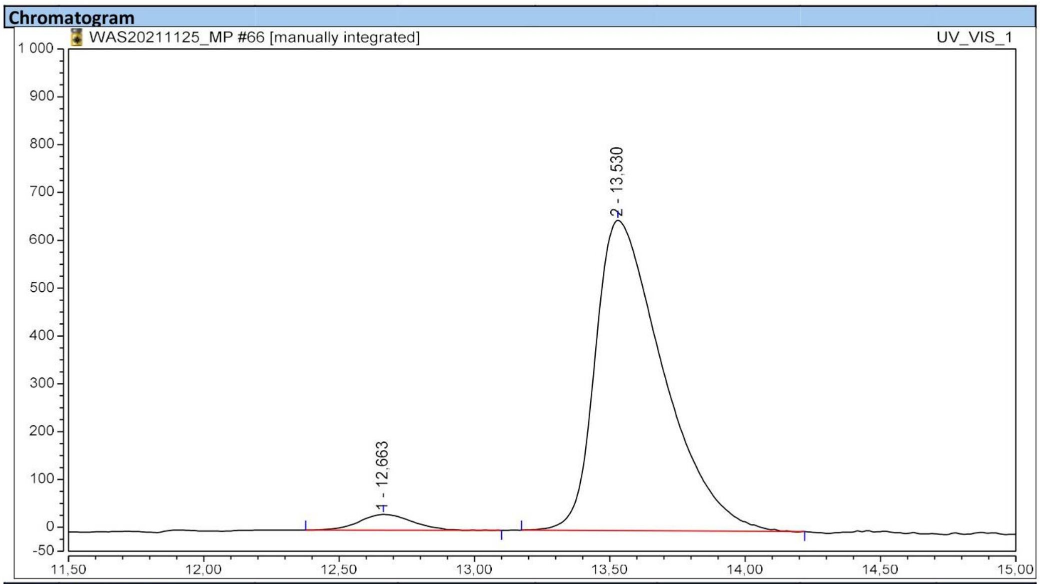

Integration Results

No.	Peak Name	Retention Time min	Area mAU*min	Height mAU	Relative Area %	Relative Height %	Amount n.a.
1		12,663	7,389	32,728	3,77	4,80	n.a.
2		13,530	188,414	648,888	96,23	95,20	n.a.
**Total:**			**195,803**	**681,616**	**100,00**	**100,00**	

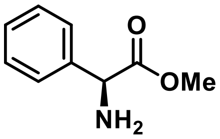

HPLC chromatograms of racemic and enantioenriched **10**

Chromatogram

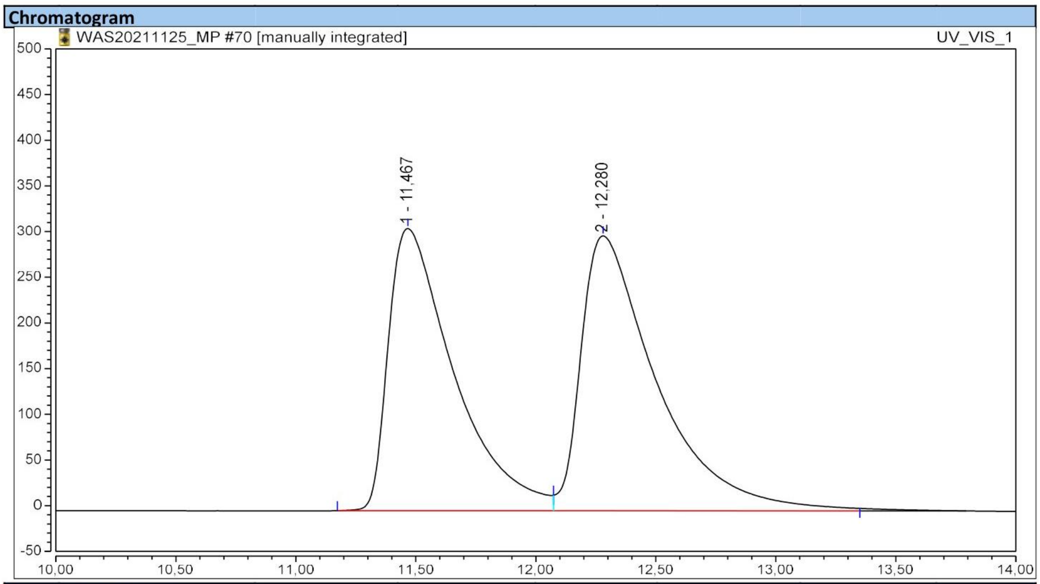

Integration Results

No.	Peak Name	Retention Time min	Area mAU*min	Height mAU	Relative Area %	Relative Height %	Amount n.a.
1		11,467	97,684	308,972	47,08	50,66	n.a.
2		12,280	109,791	300,940	52,92	49,34	n.a.
**Total:**			**207,475**	**609,912**	**100,00**	**100,00**	

Chromatogram

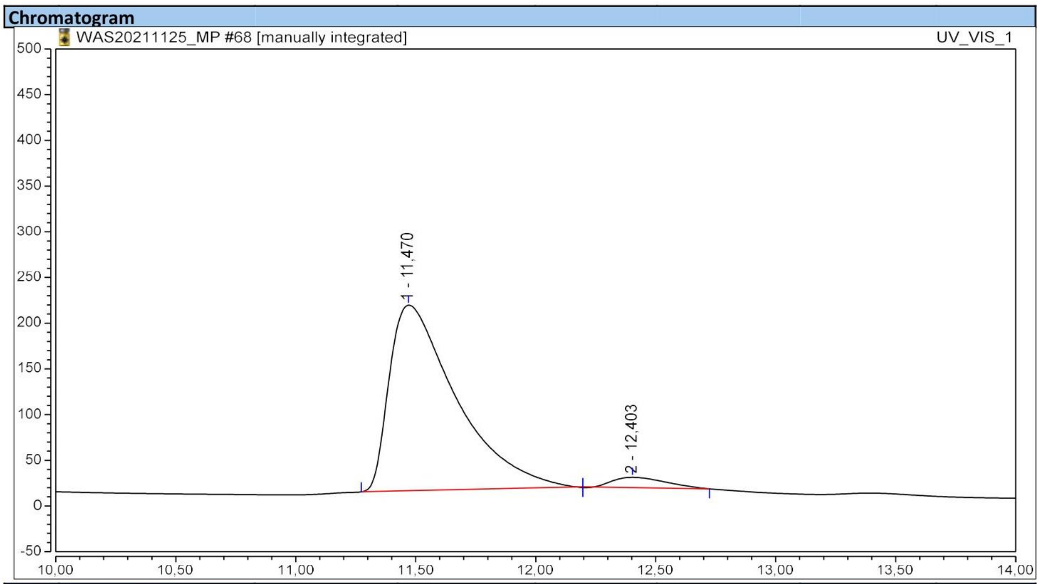

Integration Results

No.	Peak Name	Retention Time min	Area mAU*min	Height mAU	Relative Area %	Relative Height %	Amount n.a.
1		11,470	65,563	203,135	95,84	94,78	n.a.
2		12,403	2,846	11,181	4,16	5,22	n.a.
**Total:**			**68,408**	**214,317**	**100,00**	**100,00**	
